# Effectiveness of Chitosan and Its Nanoparticles Against ampC- and ESBL-Producing Pan-Drug-Resistant *Proteus mirabilis* in Egyptian Livestock

**DOI:** 10.3390/pathogens14111176

**Published:** 2025-11-18

**Authors:** Ibtisam Faeq Hasona, Amal Awad, Gamal Younis, Wafaa Farouk Mohamed

**Affiliations:** 1 Department of Bacteriology, Immunology, and Mycology, Faculty of Veterinary Medicine, Mansoura University, Mansoura 35516, Egypt; gamalyounis_2006@yahoo.com; 2 Ain-Shams University Specialized Hospital, Cairo 11588, Egypt; wafaa3010@yahoo.com

**Keywords:** chickens, buffalo, *Proteus mirabilis*, antimicrobial resistance, XDR, PDR, chitosan, chitosan nanoparticles

## Abstract

*Proteus mirabilis* (*P. mirabilis*) serves as a multi-host–pathogen regarded as an alarming foodborne infectious disease, causing illnesses of variable severity in both livestock and human beings. The present study aimed to estimate the prevalence, antibiotic susceptibility profiles, and associated antimicrobial resistance genes (ARGs) of *P. mirabilis* isolates obtained from diseased broiler chickens and native Egyptian buffaloes in Kafr El-Sheikh and Dakahlia governorates, Egypt. In addition, this study investigated the antibacterial activity of chitosan (CS) and chitosan nanoparticles (CSNPs), including the estimation of the minimum inhibitory concentration (MIC) and minimum bactericidal concentration (MBC) of CS at concentrations of 1% and 2%, as well as CSNPs. Furthermore, the sub-MIC values were utilized to assess the inhibitory effects of CS and CSNPs on swarming motility. *P. mirabilis* was detected in 68% (34/50) of broiler chickens and 40.74% (11/27) of buffaloes. Interestingly, all *P. mirabilis* isolates were tested against 21 antimicrobial drugs and showed high resistance against either critical, highly important, or important antimicrobial drugs. For chicken-originated *P. mirabilis*, 50% (17/34) of isolates were revealed to be extensively drug-resistant (XDR) and 50% (17/34) of isolates were revealed to be pan-drug-resistant (PDR). Meanwhile, 9.09% (1/11) of buffalo-originated *P. mirabilis* isolates were revealed to be XDR and 90.91% (10/11) of the isolates were revealed to be PDR. Among *P. mirabilis* isolates from broiler chickens, the prevalence of resistance genes was as follows: *int*1 (97.06%), *dfr*A1 (100%), *sul*2 (97.06%), *cat*A1 (44.12%), *aad*A1 (97.06%), *tet*(M) (81.82%), *erm*B (23.53%), *msr*A (0%), *qnr*A (47.06%), *qnr*S (0%), *gyr*A (0%), *mcr-*1 (11.76%), *bla*_TEM_ (97.06%), *bla*_CTX-M_ (26.47%), *bla*_OXA-10_ (2.94%), *bla*_CMY-2_ (41.18%), and *bla*_SHV_ (0%). The corresponding detection rates in buffalo-derived isolates were 100%, 100%, 90.91%, 63.64%, 100%, 70.59%, 18.18%, 0%, 9.09%, 0%, 0%, 18.18%, 81.82%, 18.18%, 18.18%, 63.64%, and 0%, respectively. Carbapenemase genes were found in none of the isolates from either species. CSNPs demonstrated superior antibacterial and anti-virulence activity against resistant *P. mirabilis*. CSNPs exhibited significantly lower MIC (0.067–0.081 mg/mL) and MBC (0.167–0.177 mg/mL) values compared with conventional CS formulations (MIC: 3.25–4.5 mg/mL; MBC: 6.67–9.08 mg/mL) in both broiler and buffalo isolates. In inhibition zone assays, the CSNPs + ciprofloxacin (CIP) combination showed the highest efficacy with a 50–58% increase in the inhibition area. Both CSNPs and CS 2% substantially reduced swarming motility by 45–52%, with CSNPs showing the strongest inhibitory effect. These outcomes highlight how *P. mirabilis* carries and disseminates antibiotic resistance, presenting serious threats to health policy and livestock. Also, CS or CSNPs, either alone or enhanced with CIP, are effective in vitro against resistant *P. mirabilis*, which promotes the treatment of *Proteus* infections to guarantee a bactericidal impact.

## 1. Introduction

*Proteus* spp. are extensively dispersed across several circumstances and among host beings; as an opportunistic human pathogen, it affects both human and animal gastrointestinal systems, skin, and oral mucosa, as well as waste products, soil, and plants [1]. Aside from possibly generating embryonic death, yolk sac infections, and mortality in young chickens, turkeys, and ducks, *Proteus* spp. also cause granulomatous inflammation in salt glands in waterfowl, quails, and broilers, along with arthritis, salpingitis, airsaculitis, and septicemia [2]. *Proteus mirabilis* (*P. mirabilis*) was recently isolated from cellulitis lesions of broiler chickens [3]. More specifically, ninety percent of *Proteus* spp. diseases are caused by *P. mirabilis*, which is known as a community-related illness [4]. As a zoonotic organism, *P. mirabilis* is frequently related to food-related illness, being the primary mode of dissemination of both virulent and resistant *P. mirabilis* [5], typically seen throughout livestock, along with farms [6].

*P. mirabilis* includes peritrichous flagella as a flagellum-dependent movement style, which are capable of performing distinctive “bull’s-eye” swarmed motion [7]. In turn, it is correlated with a significantly increased risk of a variety of transmissible illnesses in humans that, in appropriate circumstances, are capable of inciting infections across multiple human systems, including those of the gastrointestinal tract; extraintestinal infections of wounds, eyes, ears, the nose, the skin, the throat, burns, and the respiratory system; neonatal meningoencephalitis; empyema; and osteomyelitis. It is also the third-leading reason for exacerbated urinary tract infections, diarrhea, and has a potential role in infective endocarditis, rheumatoid arthritis, and others [8]. From earlier investigations, *P. mirabilis* was discovered in 5–18% of cases of Gram-negative bacteremia [9]. Additionally, it was previously claimed whether *P. mirabilis* and Crohn’s syndrome are possibly linked [10]. Consequently, it ultimately leads to severe infections among humans, which have a 20 to 50 percent death rate [11]. This swarming behavior is potentially regarded as a crucial virulence factor; it is a multicellular process that typically happens on hard surfaces and requires the differentiation of existing growing cells into a distinctive kind of cell category called swarmer cells [12]. It is related to both the capability for penetration cells and the generation of virulence-associated genes (VAGs) [13].

The increasing incidence of antibiotic resistance across pathogenic microbes has become another international concern, which is intimately linked to mitigating levels of serious illness and death within individuals and livestock [14]. According to the Organization for Economic Cooperation and Development’s (OECD) estimates, nearly two million individuals in Europe, North America, and Australia might die from now until 2050 if available developments in resistant bacteria remain [15]. This worrisome result emphasizes the urgency along with the worldwide scope of the resistant bacteria crisis. *P. mirabilis* exhibits strong antimicrobial resistance (AMR) and pathogenicity. This is mostly owing to *P. mirabilis* strains that have inherent resistance to nitrofurans, polymyxins (colistin), and tetracycline [16], along with having many gained antibiotic resistances to a variety of antimicrobial classes, including trimethoprim/sulfamethoxazole, aminoglycosides, carbapenems, fluoroquinolones, β-lactams, and others [17]. The fact that *P. mirabilis* is missing a chromosome-encoded beta-lactamase resulted in its naturally occurring phenotypic resistance to all lactams [17]. In turn, Extended-Spectrum Beta-Lactamase (ESBL) producers show co-resistance alongside additional kinds of antibiotics, including quinolones, aminoglycosides, and sulfa drugs [18]. According to Lim et al. [19], *P. mirabilis* was the second predominant Extended-Spectrum Beta-Lactamases-*Enterobacteriaceae* (ESBL-E) species in chickens, following *E. coli.* In contrast to ESBLs, ampC is a different kind of β-lactamase that remains unaffected by cephamycins or β-lactamase inhibitors [20]. Since the ampC processor can obscure the impact of ESBLs, recognizing them and managing their coexistence in identical strain proves extremely challenging [21]. Consequently, strains generating ampC provide a quiet store that harbors ESBLs [22].

When treating severe infections brought on by *Enterobacterales* that produce ESBLs, carbapenems are nevertheless often utilized as “last-resort” medicines [17]. The recent appearance and fast spread of carbapenemase-producing *Enterobacterales* (CPE) is a serious public health concern, as clinical treatment choices are severely restricted [17]. Consequently, carbapenem resistance poses a serious danger to the life of patients infected with multidrug-resistant Gram-negative bacteria (MDR-GNB), with overall death rates reaching 50% [23,24]. MDR-GNB resistance to carbapenems is primarily caused by the development of carbapenemases, *β*-lactamases capable of hydrolyzing carbapenems and other beta-lactam drugs [25]. Carbapenemases, including oxacillinase-48 and New Delhi metallo-beta-lactamase 1, are encoded by carbapenem resistance-determining genes (CRDGs): *bla*_VIM_, *bla*_IMP_, *bla*_KPC_, *bla*_OXA-48_, and *bla*_NDM_ [26]. Carbapenemase-producing *P. mirabilis* is particularly concerning due to its inherent resistance to nitrofurans, polymyxins, tetracycline, and tigecycline.

One of the several ways that bacteria might acquire AMR occurs via the antimicrobial resistance gene (ARG) [27]. Additionally, *P. mirabilis* had been believed to represent an appropriate host for ARG storage [28]. Given the nature of these resistant genes, they frequently appear on mobile genetic components including integrons and plasmids via mutational shifts or horizontal transfer, ending with issues concerning unsuccessful therapy along with quick dissemination [29]. The abuse of colistin and the resulting increase in plasmid-borne mobile colistin resistance genes pose an urgent risk to its application as a last-resort antimicrobial [30], expanding quickly through the transmission of horizontally located genes [31]. Especially where *mcr-*1 genes interact alongside additional resistance genes, which include ESBL, the danger of pan-drug resistance arises [32].

Infectious illness handling has become crucial for human wellness, particularly in light of the ongoing rise in MDR and the appearance of XDR or possibly PDR [33]. Thus, new antimicrobial biologics that are ecologically green and safe for overall livestock employment, along with a focus on requirements, require urgent development to manage *P. mirabilis* outbreaks instead of using antibiotics [34]. As a natural cationic non-toxic biopolymer (a linear polysaccharide comprising 1–4 linked 2-amino-deoxy β-D-glucan), chitosan is a white, hard, inelastic, and nitrogenous polysaccharide derived by the partial deacetylation of chitin [35]. It is easily accessible and possesses special qualities, including being biodegradable, biocompatible, bio-renewable, non-toxic, non-allergenic, and bio-adhesive; having no antigenic elements; and having benefits for the environment [36]. It has been demonstrated to have strong antibacterial properties, which results from it adhering to the negatively charged bacterial cell wall and then subsequently disrupting and altering membrane permeability. Additionally, chitosan is linked to bacterial genomes, which inhibit replicating DNA and ultimately cause cell death [37]. Chitosan possesses strong antibacterial effects versus a wide spectrum of foodborne illness and dangerous microbes with concentrations between 1% and 2% [38]. At present, little research is being undertaken to disclose the zoonotic potential of broiler chicken/buffalo-originated *P. mirabilis*. Thus, the main goals of this current research were to identify the prevalence of broiler chicken/buffalo-originated *P. mirabilis* in Kafr El-Sheikh and Dakahlia governorates, Egypt. Additionally, another goal was to assess the phenotypic and genotypic resistance of different classes of antimicrobial agents of the isolates, which are the main causes of high pathogenicity in *P. mirabilis* isolates. The escalating global issue of antibiotic resistance intensifies the difficulty in effectively treating infections caused by *P. mirabilis*; this, in turn, underscores the critical relevance of alternative techniques towards combating *P. mirabilis* resistance to safeguard public health worldwide. Given this, it is important to better understand how the antibacterial activities, minimum inhibitory concentrations (MICs), and minimum bactericidal concentrations (MBCs) of CS 1%, CS 2%, and chitosan nanoparticles (CSNPs) impact resistant *P. mirabilis*; investigate how they influence swarming motility (a sign of virulence); and plan how chitosans can work together with the important antibiotic ciprofloxacin (CIP) to find new treatment options for resistant infections.

## 2. Materials and Methods

Ethical approval: The bacterial isolates used in this study were collected between December 2023 and August 2024, before the institutional requirement for research ethics approval was formally enforced. When the study framework was later expanded to include additional experimental analyses, ethical approval was obtained in 2025 from the Research Ethics Committee of the Faculty of Veterinary Medicine, Mansoura University, Egypt (Approval code: MU-ACUC (VM.PhD.25.02.53)). All subsequent experiments and analytical procedures were conducted in accordance with institutional guidelines and the ARRIVE guidelines (https://arriveguidelines.org), accessed on 22 February 2025.

### 2.1. Sample Collection and Clinical Examination

A total of 720 samples were collected from diseased broiler chickens (aged 1 day to 42 days) and Egyptian native buffaloes (aged 1.5 to 2.5 years) across various localities in Kafr El-Sheikh and Dakahlia governorates in northern Egypt, between December 2023 and August 2024. Samples were obtained from both clinically diseased broiler chickens and apparently healthy buffaloes. Diseased chickens exhibited clinical signs such as diarrhea, reduced feed intake, poor growth performance, ruffled feathers, and general weakness. Post-mortem examination revealed congested and dilated intestines with watery to mucoid contents, hepatomegaly with scattered petechial hemorrhages, mild splenomegaly, and congested kidneys. Emaciation and atrophied breast muscles were also observed. Chicken samples were collected from private veterinary clinics supervising large-scale commercial farms to ensure representative sampling from the commercial production system. In contrast, buffalo samples were freshly collected after slaughter at official abattoirs from apparently healthy animals showing no clinical abnormalities before slaughter. Approximately 25 g of each tissue sample were aseptically collected from each bird or buffalo. The collected samples comprised 50 cloacal swabs and 400 internal organ samples from broiler chickens, including the liver, spleen, kidney, gall bladder, intestine, lung, meat, and gizzard (40 samples per organ). Additionally, 270 samples were obtained from buffaloes, including the liver, muscle, gall bladder, small intestine, abomasum, omasum, reticulum, rumen, and fecal matter (30 samples per type). Each sample was identified and promptly placed in an icebox and transported to the Laboratory of Bacteriology, Immunology, and Mycology, Faculty of Veterinary Medicine, Mansoura University without delay for bacteriological analysis.

### 2.2. Isolation and Identification Procedures

The samples were initially enriched in nutrient broth (HiMedia, Mumbai, India), and a loopful from the broth was streaked onto Columbia blood agar (HiMedia, Mumbai, India). The cultures were then sub-cultured onto Xylose Lysine Deoxycholate (XLD) agar (HiMedia, Mumbai, India) and incubated overnight at 37 °C, following the procedure described by Ishaq et al. [39]. Suspected *Proteus*-like colonies (red with or without a black center) were selected, purified on nutrient agar (NA) (Oxoid, Basingstoke, UK), and then transferred to 5% blood agar (Oxoid, Basingstoke, UK) and NA to check for swarming activity. The bacterial colonies exhibiting characteristics of *Proteus* spp. were subjected to staining and confirmed as *Proteus* spp. using a series of biochemical tests outlined by Markey et al. [40]. These tests included gas production, the triple sugar iron (TSI) test, motility, urease production, the IMViC tests (Indole, Methyl Red, Voges–Proskauer, and Citrate utilization), hydrogen sulfide production, catalase, oxidase, and a swarming test. All tests were incubated at 36 ± 1 °C. The identified *Proteus* spp. isolates were kept at −80 °C in nutrient broth, including 30% sterile glycerol, to enable additional verification.

### 2.3. Molecular Detection of Proteus spp. and P. mirabilis

All confirmed *Proteus* spp. isolates were sub-cultured on Luria–Bertani broth medium (Merck, Darmstadt, Germany). After 18–24 h of incubation at 37 °C, genomic DNA was extracted using the boiling method, as described previously [41]. The quantity and purity of extracted DNA were measured using a Nanodrop 1000 spectrophotometer (Thermo Scientific, Waltham, MA, USA) at 260 nm. All isolates were examined by conventional PCR for *Proteus* spp. using a specific set of primers for amplification of the 16S rRNA gene forward (5′-CACGCAGGCGGTCAATTAAG-3′) and reverse (5′-TCTTTTGCAACCCACTCCCAT-3′) primer sequence sets, with an amplified band size of 857 bp [42]. An additional set of primers targeting the *Proteus* spp.-specific 16S rRNA gene was employed to confirm the identification of the isolates as *P. mirabilis*, including 27F: 5′-AGAGTTTGATCCTGGCTCAG-3′ and 1495R: 5′-CTACGGCTACCTTGTTACGA-3′ with an amplified band size of 1496 bp [43]. The amplification was carried out on a thermal cycler (Mastercycler, Eppendorf, Hamburg, Germany). In the PCR tube, the reaction was performed on a total volume of 25 μL of mixture, which contained 12.5 μL of 2X ABT Red Mix (Applied Biotechnology Co., Ltd., Ismailia, Egypt), 1 μL of each primer, and 5 μL of DNA template. The remaining volume was filled with sterile nuclease-free water, then mixed well by vortex. The suitable PCR process for specific 16S rRNA gene amplification of *Proteus* spp. and *P. mirabilis* amplification was initial denaturation, denaturation, annealing, and extension at 95 °C for 5 min, 35 cycles at 95 °C for 30 s, 58 °C for 30 s, and 72 °C for 1 min, respectively, where the final extension was conducted at 72 °C for 10 min and then held at 4 °C. To allow standardization, 100 bp DNA ladders (Applied Biotechnology Co., Ltd., Ismailia, Egypt) were utilized as molecular markers. The test DNA was replaced with 5 μL of nuclease-free water, providing the negative control. PCR-amplified products were electrophoresed in 1% (*w*/*v*) agarose gels and stained with ethidium bromide. After that, the gel was then visualized and photographed under a UV transilluminator.

### 2.4. Antimicrobial Susceptibility Testing

The phenotypic antimicrobial susceptibility of *P. mirabilis* isolates was analyzed against 21 antimicrobial agents (Oxoid, Basingstoke, Hampshire, UK) belonging to fourteen antibiotic classes using the Kirby–Bauer disk diffusion assay. The results were categorized as sensitive and resistant. Depending upon the diameters of the zone of inhibition, the thin veil of swarming growth was ignored as described by the standards and interpretive criteria of CLSI [44]. If an isolate was intermediate or resistant to a particular antibiotic, it was classified as non-susceptible [45]. A 100 µL aliquot of a bacterial suspension, standardized to a 0.5 McFarland turbidity standard, was aseptically inoculated onto Mueller–Hinton agar (MHA; HiMedia, Mumbai, India) plates and spread uniformly. The inoculated plates were subsequently air-dried for 10–15 min. Antimicrobial disks were then aseptically placed onto the agar surface, ensuring optimal antibiotic diffusion. Finally, all plates were incubated at 37 °C for 24 h. Based on the importance of antimicrobials in human and veterinary medicine, the World Health Organization [46] categorized the following antimicrobial classes that were used into critically important antimicrobials, highly important antimicrobials, and important antimicrobials. The antimicrobials tested are summarized in Table 1, along with their drug class, abbreviations, and disk content. As a quality control, the *E. coli* reference strain ATCC 25922 was used. Based on their phenotypic antimicrobial resistance profiles, *P. mirabilis* isolates were divided into three categories: multidrug-resistant (MDR), which demonstrated resistance to three or more antibiotic groups; extensively drug-resistant (XDR), which demonstrated resistance to all tested antibiotic classes except one or two; and pan-drug-resistant (PDR), which demonstrated resistance to all antibiotics in all antibiotic classes examined [14].

### 2.5. Detection of Antimicrobial Resistance Genes (ARGs), β-Lactamase-Encoding Genes, ampC-Encoding Gene, Colistin Resistance Gene, and Integron Integrase Gene

We analyzed twenty-four antimicrobial resistance targets (ARTs) from twelve classes. These included both acquired resistance genes and chromosomal mutation targets: aminoglycosides (*aad*A1), chloramphenicol (*cat*A1), plasmid-mediated quinolone resistance (PMQR) (*qnr*A and *qnr*S), fluoroquinolone resistance target (*gyr*A), sulfonamide (*sul*2), tetracyclines (*tet*(M)), macrolides (*erm*B and *msr*A), trimethoprim (*dfr*A1), and Fosfomycin (*fos*A). The analyzed β-lactamase genes encompassed narrow-spectrum (*bla*_TEM_ and *bla*_SHV_), oxacillinase (*bla*_OXA-10_), ESBL (*bla*_CTX-M_), ampC (*bla*_CMY-2_), and carbapenemase types *bla*_KPC_, *bla*_OXA-48_, *bla*_VIM_, *bla*_IMP_, *bla*_GES_, and *bla*_NDM-1_). Furthermore, the presence of the colistin resistance gene *mcr-*1 and the class 1 integron integrase gene *int*1 was also investigated. The primer sequences, target genes, amplicon size of the used genes, annealing temperature, and the relevant references are summarized in Table 2. Initial optimization focused on multiplex panels for the simultaneous detection of multiple targets. Compatible primer pairs that co-amplified efficiently were then consolidated into duplex assays. Any target that demonstrated inconsistent amplification in these formats was successfully re-amplified in a uniplex PCR. Accordingly, all strains were analyzed using various PCR amplification techniques: duplex PCR for the detection of *bla*_TEM_ and *bla*_CTX-M_ genes and multiplex PCR targeting the *dfr*A1, *qnr*A, and *sul*2 genes. The remaining genes were examined using conventional uniplex PCR assays. For all these reactions, the total volume was 25 μL comprising 12.5 μL of 2X ABT Red Mix, 5 μL of the template DNA, 1 μL of each oligonucleotide primer, and 5.5 μL of nuclease-free water, which were then mixed well by vortex. All PCR amplification conditions were an initial denaturation for 5 min at 94 °C, 35 cycles of denaturation at 95 °C for 30 s, and extension at 72 °C for 60 s—annealing temperatures are shown in Table 2. The final extension was carried out at 72 °C for 10 min. Amplicons were electrophoresed as described above.

### 2.6. Chemicals Used and Preparation of Chitosan and Chitosan Nanoparticles Solution

Low-molecular-weight (LMW) extra-pure edible chitosan (CS) (≥95% degree of deacetylation (DA)), acetic acid, sodium hydroxide (NaOH), and sodium triphosphate (TPP) were purchased from Sigma-Aldrich (St. Louis, MO, USA).

CSNPs were created by linking TPP to CSNPs using the ionotropic gelation process, which depends upon an electrostatic connection between positively charged chitosan and negatively charged TPP. A 1% *w*/*v* aqueous acetic acid solution containing 0.25 mg/mL of chitosan was prepared, magnetically stirred overnight at room temperature to create a clear solution, and then the pH of the solution was raised from approximately 3.7–3.9 to 5.0 via titration with 1 M NaOH. The CS in the resulting solution was cross-linked with 1% TPP before being filtered through a 0.45 m syringe filter and sonicated at 1.5 kW for 30 min using Ultrasonic Homogenizers HD 2070. Then, the suspension was centrifuged for 10 min at 12,000× *g*. The precipitate (CSNPs) was washed twice with distilled water before being centrifuged again and freeze-dried. The freeze-dried CSNPs may be suspended in water for characterization or utilized directly in other investigations. Chitosan solutions of 1% (10 mg/mL) and 2% (20 mg/mL) were prepared by dispersing 1 g and 2 g of CS in 1% and 2% acetic acid solutions, respectively. The chitosan solution was freshly prepared for each day’s assays.

### 2.7. Characterizations of CSNPs

#### 2.7.1. Particle Size Analyzer (PSA) and Zeta Potential (ZP)

A particle size analyzer (PSA) was utilized. The electrophoretic mobility (zeta potential) (ZP) of nanoparticles of freshly prepared CSNPs were studied using a Zetasizer-ZS Ver. 7.01 (Malvern Instruments Limited, Malvern, UK) as described by Muller et al. [68].

#### 2.7.2. UV–Visible Spectroscopy

The biological development of nanoparticles has been proven through observing how they formed using UV–visible (UV–Vis) spectroscopy for determining their absorption band. The manufactured CSNPs’ UV–Vis absorption spectra were analyzed within a range of 190–800 nm via a spectrophotometer (Edinburgh Instruments Ltd. DS5 Dual Beam UV–Vis spectrophotometer, Livingston, Scotland) operating at a resolution of 1 nm.

#### 2.7.3. Fourier-Transform Infrared (FTIR) Spectroscopy

FTIR spectra of CSNPs were captured using an FTIR spectrophotometer (Bruker-Tensor 27, Bremen, Germany) in order to identify particular chemical groups within the analyzed materials. The spectral range of 4000–400 cm^−1^ was detected using the preceding technique [69].

#### 2.7.4. Transmission Electron Microscopic Observation of CSNPs

The morphology of CSNPs was investigated using a transmission electron microscope (TEM) (JEOL, JEM-2100, JEOL Ltd., Tokyo, Japan). Briefly, the dried CSNPs were dispersed in ethanol by brief sonication, and a drop of the suspension (200 µL) was placed onto a 400-mesh carbon-coated copper grid covered with a nitrocellulose film. After drying, the samples were examined under TEM to observe particle size and morphology, as described by Ali et al. [70].

### 2.8. Evaluation of Antibacterial Assay of CSNPs, CS 1%, and CS 2%

The CSNPs, CS 1%, and CS 2% were assessed for their antibacterial effectiveness versus resistant *P. mirabilis* by agar well diffusion assay, as described by EUCAST [71]. Briefly, 100 µL of *P. mirabilis* suspension containing 10^8^ CFUs/mL was swabbed uniformly utilizing a sterilized L-shaped rod on the MHA surface to obtain uniform bacterial growth. Using a sterile cork-borer with a diameter of 6 mm, a hole was punched from agar, and the bottoms of the wells were sealed by pouring a drop of molten MHA in. Then 100 µL of the CS 1% (10 mg/mL), CS 2% (20 mg/mL), and CSNPs 0.25 mg/mL, and a combination of CIP 5 µg separately with 100 µL of the CS 1%, with 100 µL of the CS 2%, and with 100 µL of the CSNPs (0.25 mg/mL) were introduced into different wells. Deionized water was used as a negative control, whereas 100 µL of CIP 5 µg/mL was used as a positive control. The plates were refrigerated for 30 min to ensure adequate dispersion of the substances being studied alongside the control sample before being moved to an incubator at 37 °C for 24 h. Upon completion of the incubation period, the growth-inhibitory impact of the antimicrobial agents was evaluated using the diameter of the zone of inhibition, defined in millimeters (mm). The enhancing interaction of commercial antibiotic CIP alone was compared to CIP separately with CS (1%), CS (2%), and CSNPs quantified by the fold increase in the zone of inhibition. This was calculated by dividing the inhibition area of the combination disk by that of the CIP-only disk, where the area (mm^2^) was defined as π × (radius)^2^. For both broiler chicken and buffalo isolate groups, the median inhibition area was determined from all samples within each cohort. The resulting fold increase was then expressed as the group median. All measurements were conducted in triplicate.

### 2.9. Evaluation of Minimum Inhibitory Concentration (MIC) and Minimum Bactericidal Concentration (MBC)

The MIC and MBC of CS 1%, CS 2%, and CSNPs were determined against resistant *P. mirabilis*, applying the procedure outlined in the CLSI criteria [44]. The MIC of antibacterial agents was determined in a 96-well round bottom microtiter plate (Lab Systems, Helsinki, Finland) using standard broth microdilution methods. For this test, 100 μL of Mueller–Hinton broth (MHB) was added to the 1st to 12th wells; after that, 100 μL of antibacterial agents (CS 1%, CS 2%, and CSNPs) was introduced to the first well, and two-fold serial dilutions with concentrations that range from 10 to 0.020 mg/mL, 20 to 0.039 mg/mL, and 0.25 to 0.00049 mg/mL were performed in wells 1 to 10, respectively. An overnight culture of *P. mirabilis* was harvested from nutrient agar plates and standardized to 0.5 McFarland turbidity using sterile saline. This suspension was subsequently diluted in MHB to achieve the target inoculum concentration of 10^6^ CFU/mL for broth microdilution assays. Both visual assessment and spectrophotometric verification at 600 nm ensured standardized inoculum preparation across all experimental replicates. Then 100 μL of bacterial inoculum was added to all the wells except the negative control well. Well 1 of the microtiter plates contained the highest concentration of antibacterial agents, while well 10 contained the lowest concentration. Well 11 served as a positive control (medium and bacterial inoculum), and well 12 served as a negative control (only medium). Following a 24 h incubation period at 37 °C, resazurin solution (0.015% *w*/*v*) was aliquoted (30 μL/well) into 96-well plates as a cell viability indicator. The plates were further incubated for 1–4 h to allow for metabolic color conversion. Resazurin was prepared at a 0.015% concentration by dissolving 0.015 g of resazurin, then vortexing and filtering with a 0.22 µm filter paper, and storing at 4 °C at a maximum for two weeks after preparation [72]. In response to the activity of the reductase enzyme, blue resazurin was reduced to pink resorufin, reflecting the vitality of microbial cells. The well with a blue/purple tint (no coloring change) after the incubation period of 4 h showed a minimal quantity of antimicrobial substances that prevent microbial growth without causing any observable growth and was thus assigned an MIC value [73]. The capacity of CS 1%, CS 2%, and CSNPs to have a bactericidal impact was then tested, with MBC values recorded against resistant *P. mirabilis*. For MBC determination, 50 µL aliquots were aseptically collected from non-turbid wells demonstrating complete growth inhibition prior to resazurin introduction. These wells, initially identified as the MIC endpoints through visual assessment, were subsequently verified using the resazurin viability assay. The aliquots were sub-cultured on resazurin-free Mueller–Hinton agar plates and incubated at 37 °C for 24 h. The MBC was defined as the lowest concentration resulting in no colony formation. Three duplicates of every experiment were conducted. In addition to MIC, MBC is thought to be the lowest concentration of antibacterial drugs, killing 99.9% of a bacterial culture without producing discernible growth on the MHA plate [74]. Bacterial growth ought to indicate that those microbes were present within this original well. Conversely, if no growth was seen as well as the original well lacking viable microbes, the antibacterial agent might be considered bactericidal at the exact dose [75]. The bactericidal or bacteriostatic impact of the investigated substances was determined by calculating the MBC/MIC ratio to determine their efficacy. According to French [76], if the MBC/MIC ratio is less than four times the MIC, the investigated substances have bactericidal properties.

### 2.10. Scanning Electron Microscopic Observation of the Antibacterial Efficacy of CS and CSNPs on Resistant P. mirabilis

The antibacterial activity of CS and CSNPs against *P. mirabilis* was examined independently using a JEOL JSM-IT100 scanning electron microscope (SEM) (JEOL Ltd., Tokyo, Japan). *P. mirabilis* was cultivated in MHB with a sub-MIC conc. of CS and CSNPs and incubated for 24 h at 37 °C and was subsequently collected through centrifuging gently (5000 r/5 min). After that, the pellets were rinsed three times using 0.1 M phosphate-buffered saline (PBS) (pH 7.4) and preserved in 2.5% glutaraldehyde overnight at 4 °C. The pellets had to be washed three times for 20 min each with 0.1 M PBS (pH 7.4) before undergoing dehydration utilizing an ethanol gradient (30, 50, 70, 80, 95, 100, 100%) for 15 min at each stage. According to Jeong et al. [77], each sample was dried using the critical-point dryness procedure, sputter-coated with gold, and inspected using SEM.

### 2.11. Investigation of the Effect of CS and CSNPs as Anti-Swarming Agents

In Eppendorf tubes, 50 μL of the *P. mirabilis* cultured overnight was adjusted to a concentration of 0.5 McFarland in a tube. It was subsequently thoroughly combined with the sub-MIC of CS and CSNPs separately and incubated for 24 h at 37 °C. In the subsequent incubation, 3 μL of the aforementioned culture was placed in the middle of the blood agar plates and allowed to grow under the same conditions as previously mentioned. Swarm diameter waves were observed emanating through the inoculation center, measured in millimeters (mm), and compared to the control (bacterial plate swarming without CS administration or CSNPs). To determine the median diameter, the test was repeated three times.

### 2.12. Statistical Analysis

All statistical analyses were performed using jamovi software (version 2.7.6; The jamovi Project, Sydney, Australia). The Shapiro–Wilk test revealed that most continuous variables deviated from normality, warranting the use of non-parametric tests throughout the study. Data are presented as median with interquartile range (IQR) for continuous variables and as frequencies (percentages) for categorical variables. Within-species comparisons of antibiotic effects on inhibition zones, MIC, and MBC were analyzed using the Friedman test with Durbin–Conover’s post hoc analysis for pairwise comparisons. Between-species differences in these parameters were assessed using the Mann–Whitney U test. Categorical data associations were evaluated with Chi-square or Fisher’s exact test, as appropriate. Statistical significance was set at *p* < 0.05 for all analyses. Antimicrobial resistance profiles, resistance types, and resistance genes were presented in a binary heatmap with annotations through Python version 3.10 using Google Colaboratory, leveraging libraries including Seaborn version 0.13.2, Pandas version 2.2.2, NumPy version 2.0.2, Matplotlib version 3.10.0. Furthermore, analysis of coexisting antimicrobial resistance genes was illustrated using a correlation heatmap with hierarchical clustering through Python version 3.10 using Google Colaboratory, leveraging libraries including Seaborn version 0.13.2, Pandas version 2.2.2, NumPy version 2.0.2, and Matplotlib version 3.10.0, and SciPy version 1.16.3.

## 3. Results

### 3.1. Prevalence of P. mirabilis Among Proteus spp.

**Figure 1:**
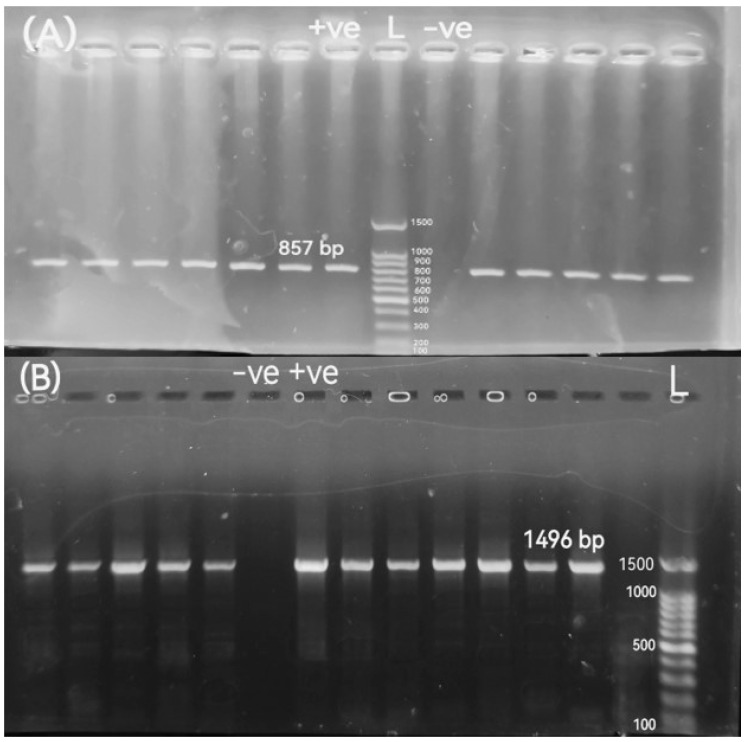
Agarose profile for the detection of *Proteus* spp.-specific 16S rRNA is shown at 857 bp (**A**), and *P. mirabilis*-specific 16S rRNA is shown at 1496 bp (**B**). Lane L: 100 bp ladder as a molecular size DNA marker. Lane +ve: control positive. −ve: control negative.

Out of 720 samples, *Proteus* spp. isolates were recorded in 11.11% (50/450) and 10% (27/270) of diseased broiler chickens and native Egyptian buffaloes, respectively. All *Proteus* spp. isolates were examined for the *P. mirabilis* 16S rRNA-specific gene by conventional PCR (Figure 1) (Figures S1 and S2 at Supplementary File S1). As a result of the molecular screening, 34 (68%) isolates and 11 (40.74%) isolates carrying the *P. mirabilis* 16S rRNA-specific gene were detected in diseased broiler chickens and buffaloes, respectively. Out of 50 *Proteus* isolates, the percentage positivity of *P. mirabilis* in diseased broiler chickens was meat 80% (8/10); liver 60% (3/5); gall bladder 71.43% (5/7); gizzard 50% (4/8); intestine 57.14% (4/7); spleen 85.71% (6/7); and cloacal swabs 66.67% (4/6). The occurrence of *P. mirabilis* isolates in native Egyptian buffalo samples was meat 40% (2/5); liver 33.33% (1/3); rumen 75% (3/4); abomasum 75% (3/4); and fecal matter 50% (2/4). The distribution of *Proteus* spp. and *P. mirabilis* isolates among various sample sources is shown in Table 3.

### 3.2. Phenotypic Antimicrobial Resistance Profile of P. mirabilis Isolates

Interestingly, all *P. mirabilis* isolates originated from the broiler chickens recovered (100%, 34/34) and demonstrated complete resistance against furazolidone, doxycycline, clarithromycin, tetracycline, sulfamethoxazole/trimethoprim, amoxicillin, erythromycin, gentamicin, streptomycin, chloramphenicol, metronidazole, rifampin, ampicillin/sulbactam, amoxicillin/clavulanic acid, and cefadroxil. Additionally, 94.12%, 91.18%, 91.18%, 88.24%, 85.29%, and 79.41% of the isolates showed resistance against cefaclor, ciprofloxacin, levofloxacin, aztreonam, cefixime, and meropenem, respectively. On the other hand, complete resistance was observed in buffalo-originated *P. mirabilis* isolates against furazolidone, doxycycline, tetracycline, sulfamethoxazole/trimethoprim, clarithromycin, meropenem, amoxicillin, erythromycin, gentamicin, streptomycin, metronidazole, rifampin, chloramphenicol, ampicillin/sulbactam, amoxicillin/clavulanic acid, aztreonam, cefadroxil, cefaclor, and cefixime, and high resistance (90.91%) was observed against ciprofloxacin, and levofloxacin. In the present investigation, broiler chicken-originated *P. mirabilis* isolates (97.06%, 33/34) showed beta-lactamase properties according to their antibiotic resistance profiles. On the other hand, the *P. mirabilis* 50% (17/34) isolates showed resistance to 11 (20.59%, 7/34) or 12 (29.41%, 10/34) out of the 13 antibiotic classes revealed as XDR with a MAR index of 0.846 and 0.923, respectively, and 50% (17/34) of isolates resistant to 13 out of the 13 antibiotic classes were revealed as PDR with a MAR index of 1. Buffalo-originated *P. mirabilis* isolates showed 100% beta-lactamase properties. Furthermore, 9.09% (1/11) of *P. mirabilis* isolates showed resistance to 11 out of the 13 antibiotic classes revealed as XDR with a MAR index 0.846, and 90.91% (10/11) of isolates were resistant to 13 out of the 13 antibiotic classes revealed as PDR with an MAR index of 1, which is an interesting finding that indicates the excessive use of antibiotics in veterinary farms in Egypt. The multiple antibiotic resistance (MAR) indices of *P. mirabilis* isolates recovered from broiler chickens and buffaloes are detailed in Table 4.

### 3.3. Detection of Antibiotic Resistance Genes, β-Lactamase-Encoding Genes, ampC-Encoding Genes, Carbapenemase Genes, Colistin Resistance Genes, and Integron Integrase Class 1

**Figure 2:**
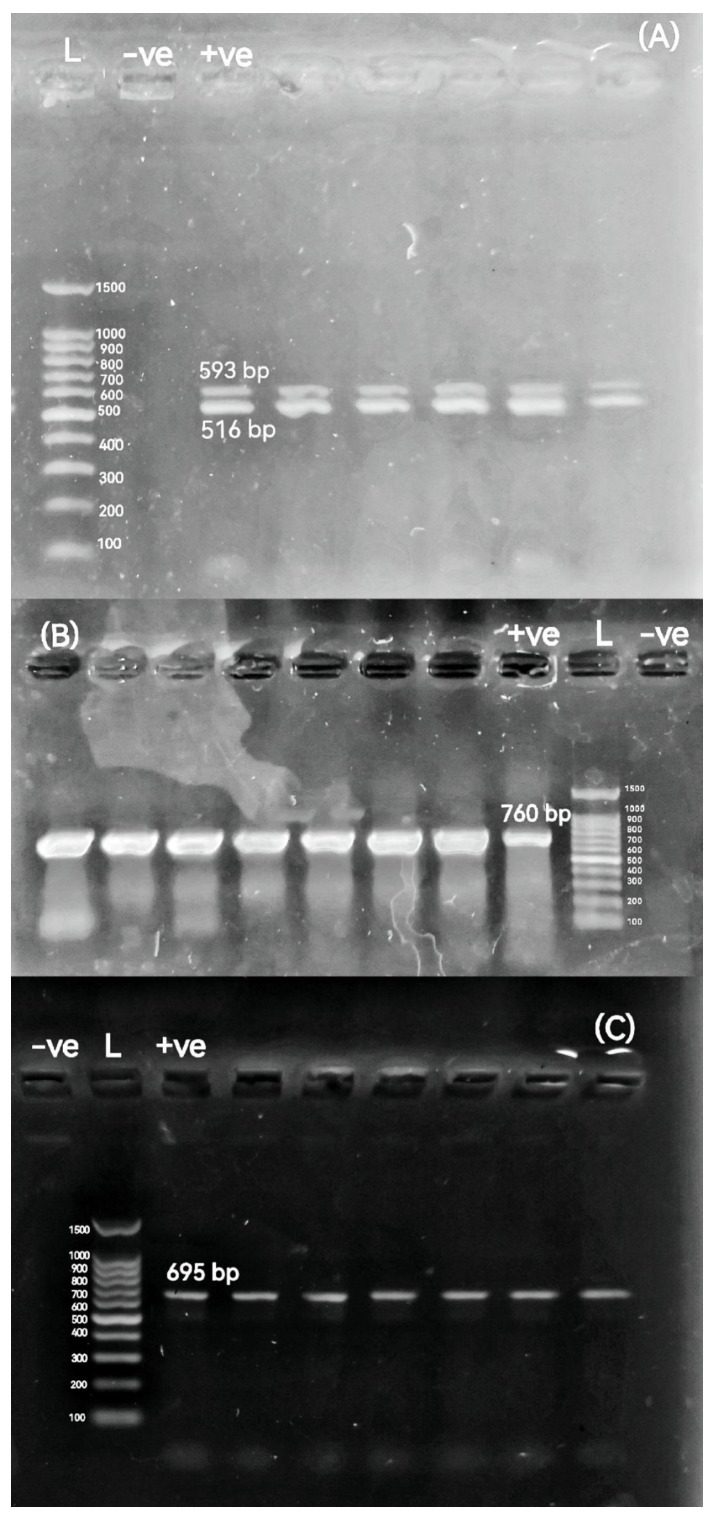
Agarose profile for the detection of resistance gene (**A**) duplex PCR of *bla*_CTX-M_ (593 bp); *bla*_TEM_ (516 bp); (**B**) *bla*_OXA-10_ (760 bp); (**C**) *bla*_CMY-2_ (695 bp). Lane L: 100 bp ladder as a molecular size DNA marker. Lane +ve: control positive. −ve: control negative.

**Figure 3:**
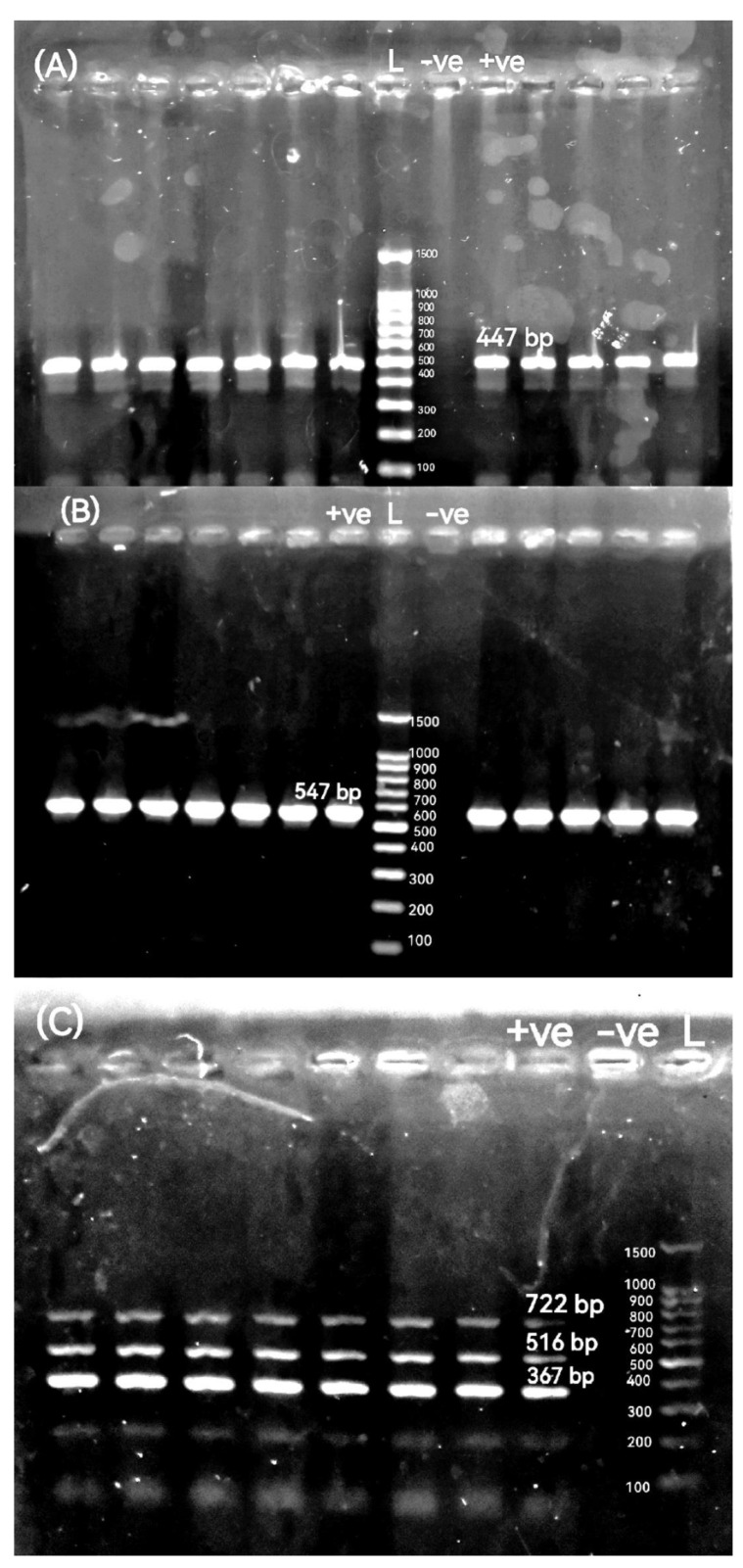
Agarose profile for the detection of resistance gene (**A**) *aad*A1 (447 bp); (**B**) *cat*A1 (547 bp); (**C**) multiplex PCR *dfr*A1 (367 bp), *qnr*A (516 bp), and *sul*2 (722 bp). Lane L: 100 bp ladder as a molecular size DNA marker. Lane +ve: control positive. −ve: control negative.

**Figure 4:**
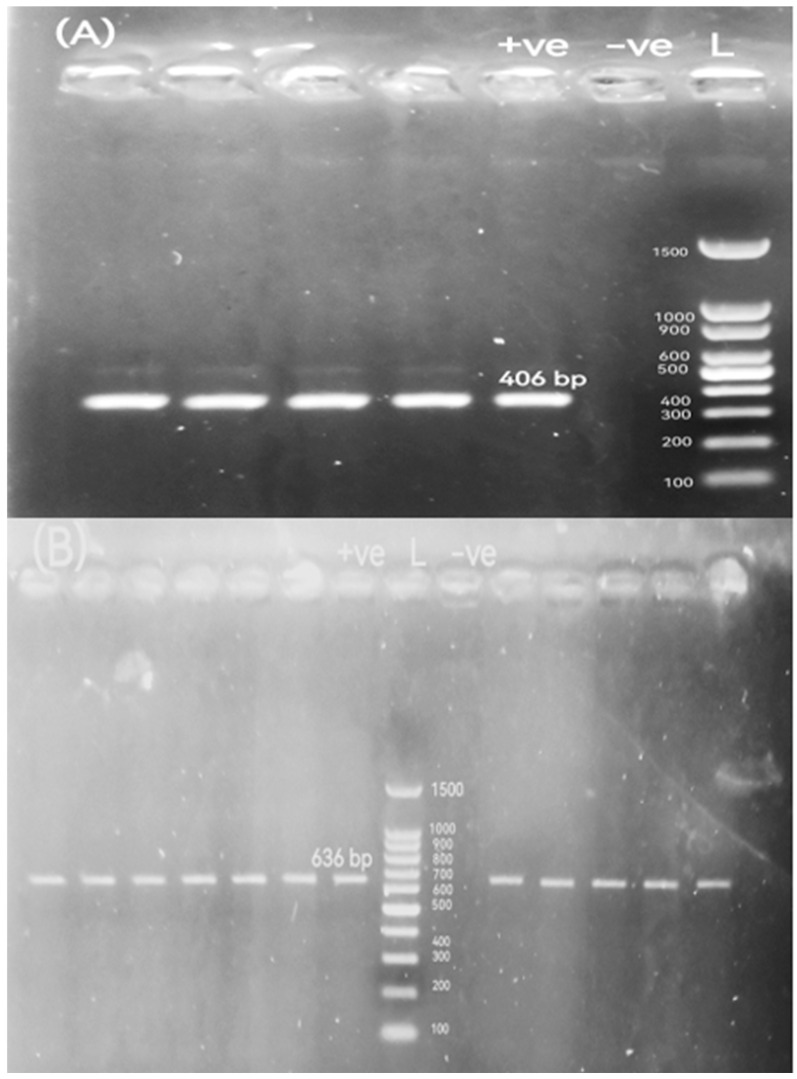
Agarose profile for the detection of resistance gene (**A**) *tet*(M) (406 bp) and (**B**) *erm*B (636 bp). Lane L: 100 bp ladder as a molecular size DNA marker. Lane +ve: control positive. −ve: control negative.

**Figure 5:**
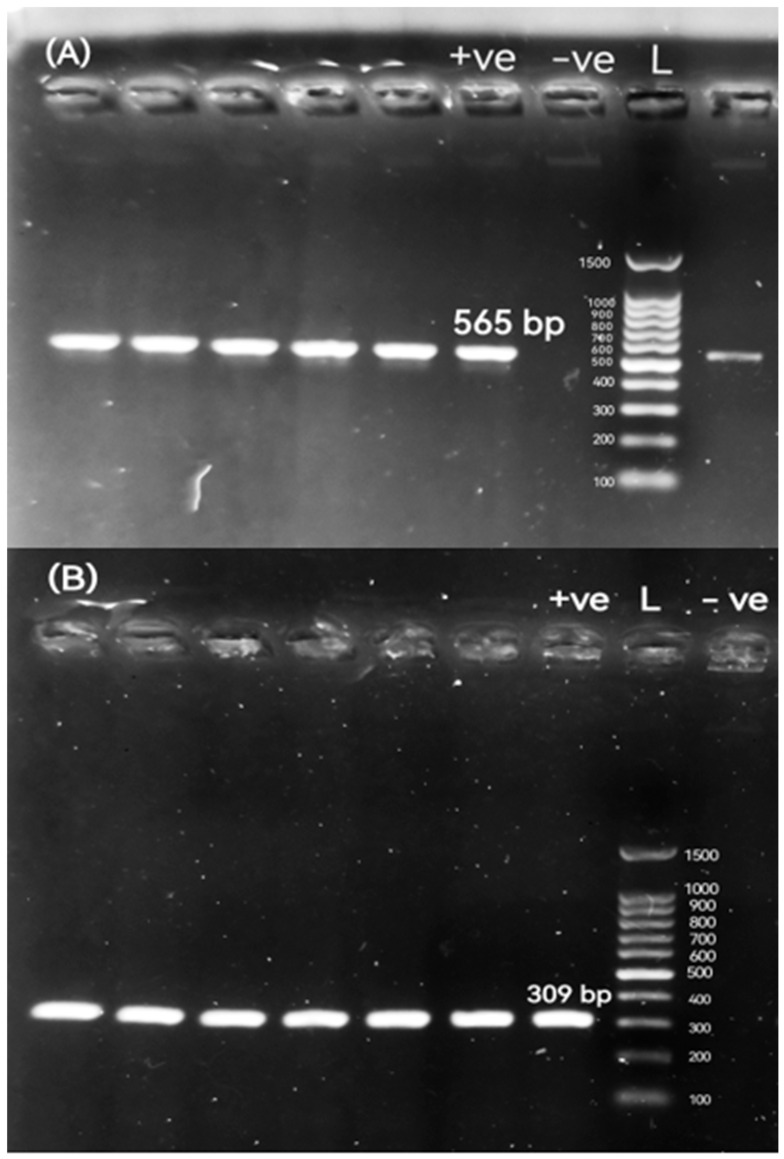
Agarose profile for the detection of resistance gene (**A**) *int*1 (565 bp) and (**B**) *mcr-*1 (309 bp). Lane L: 100 bp ladder as a molecular size DNA marker. Lane +ve: control positive. −ve: control negative.

**Figure 6:**
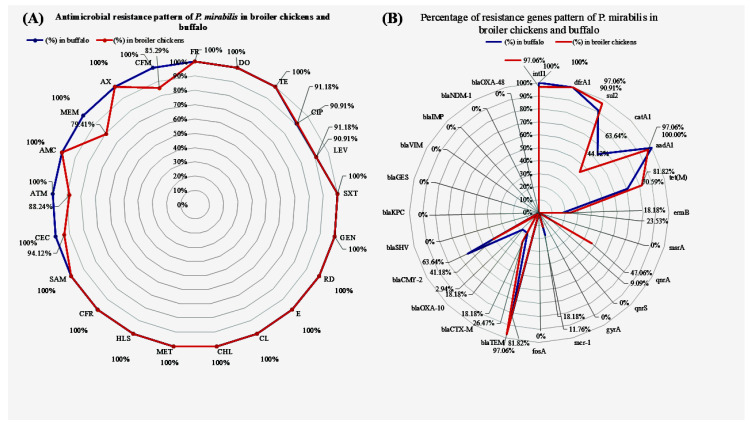
Overall antibiotic resistance pattern (**A**) and antibiotic resistance genes (**B**) of *P. mirabilis* isolates from broiler chickens and buffaloes.

**Figure 7:**
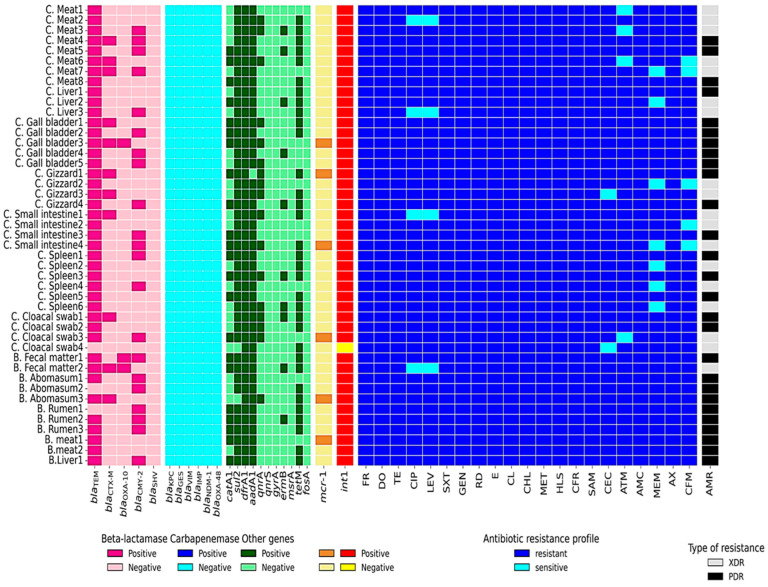
A heatmap representing beta-lactamase genes (*bla*_TEM_, *bla*_CTX-M_, *bla*_OXA-10_, ampC type (*bla*_CMY-2_), and *bla*_SHV_), carbapenemase genes (*bla*_KPC_, *bla*_GES_, *bla*_VIM_, *bla*_IMP_, *bla*_NDM-1_, and *bla*_OXA-48_), other genes (*cat*A1, *sul*2, *dfr*A1, *aad*A1, *qnr*A, *qnr*S, *gyr*A, *erm*B, *msr*A, *tet*(M), and *fos*A), colistin resistance gene (*mcr-*1), integron integrase gene (*int*1), and resistance profiles of each isolate to different antibiotics (furazolidone (FR), doxycycline (DO), tetracycline (TE), ciprofloxacin (CIP), levofloxacin (LEV), sulfamethoxazole/trimethoprim (SXT), gentamicin (GEN), rifampin (RD), erythromycin (E), clarithromycin (CL), chloramphenicol (CHL), metronidazole (MET), streptomycin (HLS), cefadroxil (CFR), ampicillin/sulbactam (SAM), cefaclor (CEC), aztreonam (ATM), amoxicillin/clavulanic acid (AMC), meropenem (MEM), amoxicillin (AX), cefixime, (CFM)), and type of resistance.

The percentages of resistance genes were observed in broiler chickens-originated *P. mirabilis* isolates as 97.06% *int*1, 100% *dfr*A1, 97.06% *sul*2, 44.12% *cat*A1, 97.06% *aad*A1, 81.82% *tet*(M), 23.53% *erm*B, 0% *msr*A, 47.06% *qnr*A, 0% *qnr*S, 0% *gyr*A, 11.76% *mcr-*1, 0% *fos*A, 97.06% *bla*_TEM_, 26.47% *bla*_CTX-M_, 2.94% *bla*_OXA-10_, 41.18% *bla*_CMY-2_, 0% *bla*_SHV_, 0% *bla*_KPC_, 0% *bla*_GES_, 0% *bla*_VIM_, 0% *bla*_IMP_, 0% *bla*_NDM-1_, and 0% *bla*_OXA-48_. All of the above resistance genes were observed in buffalo-originated *P. mirabilis* isolates as 100%, 100%, 90.91%, 63.64%, 100%, 70.59%, 18.18%, 0%, 9.09%, 0%, 0%, 18.18%, 0%, 81.82%, 18.18%, 18.18%, 63.64%, 0%, 0%, 0%, 0%, 0%, 0%, and 0%, respectively. Agarose profiles for the detection of resistance genes are shown in Figure 2, Figure 3, Figure 4 and Figure 5, and Supplementary File S1 (Figures S3–S12). Also, Figure 6 illustrates the frequency of antibiotic resistance phenotypes and genotypes of *P. mirabilis* isolated from broiler chickens and buffalo. A comparison of the prevalence of β-lactamase genes among β-lactamase-producing *P. mirabilis* isolates revealed that 97.06% (33/34) of isolates from broiler chickens and 81.82% (9/11) of those from buffalo carried these genes. The phenotypic and genotypic resistance profiles of XDR and PDR *P. mirabilis* isolates are illustrated in Figure 7.

### 3.4. Detection of Association Between ESBL/ampC and qnrA-, mcr-1-, dfrA1-, aadA1-, sul2-, catA1-, tet(M)-, and ermB-Encoding Resistance Genes

**Figure 8:**
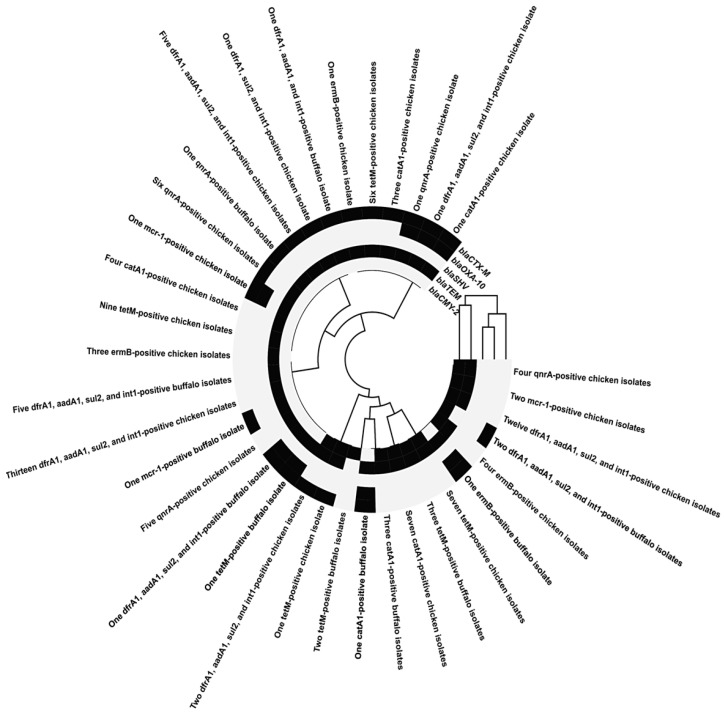
A circular heatmap that represents the co-existence of beta-lactamase genes (*bla*_TEM_, *bla*_CTX-M_, *bla*_OXA-10_, ampC type (*bla*_CMY-2_), and *bla*_SHV_) alongside other genes (*cat*A1, *sul*2, *dfr*A1, *aad*A1, *qnr*A, *erm*B, *tet*(M), *mcr-*1, and *int*1) found within isolates. Black color represents positive, and white represents negative.

**Figure 9:**
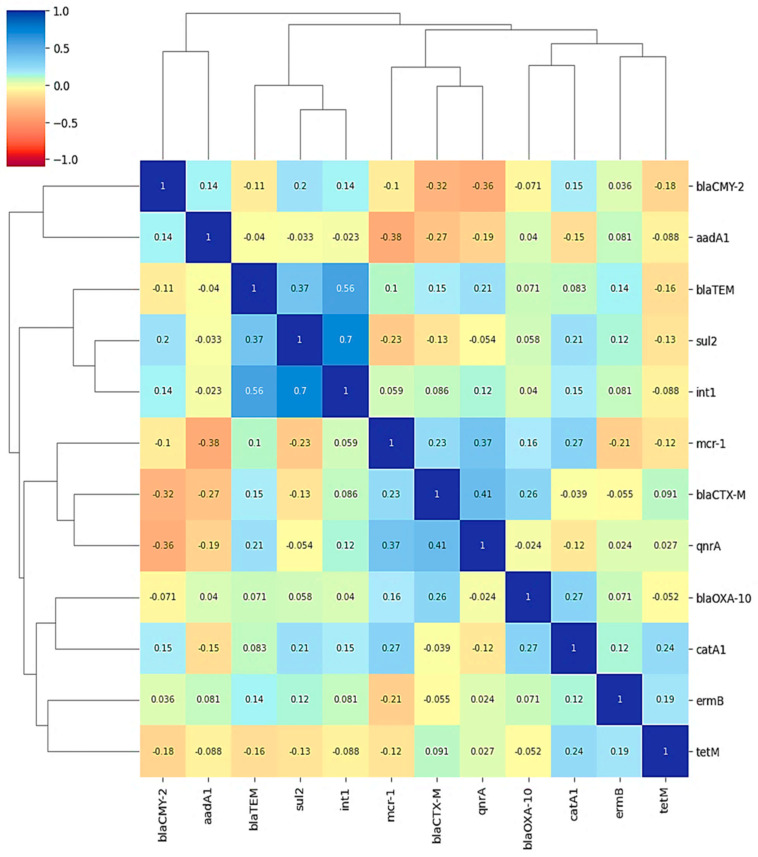
A correlation heatmap depicting the co-occurrence patterns of discovered antimicrobial resistance genes. The technique uses hierarchical clustering to group genes based on the similarity of their correlation profiles, and the generated dendrograms are shown on the axes. The correlation coefficient is color-coded, with dark blue indicating a strong positive correlation (approaching +1.0), red indicating a strong negative correlation (approaching −1.0), and pale yellow representing a weak or no association (around 0.0). Strong positive relationships were seen, particularly within the cluster that included *sul*2, *int*1, and *bla*_TEM_. Genes that were either absent or present in all samples were removed from this study since their correlation coefficients could not be determined variably.

To analyze the links among the identified resistance genes, a correlation matrix was created and shown as a heatmap with hierarchical clustering. Figure 8 depicts all co-occurrences of beta-lactamase genes with other genes across isolates. The analysis found multiple clusters of positively co-occurring genes, including a substantial relationship between *sul*2, *int*1, and *bla*_TEM_ (Figure 9).

The frequency of *qnr*A positivity in broiler chicken-originated *P. mirabilis* isolates was significant among six *bla*_TEM_- and *bla*_CTX-M_-producing isolates, five *bla*_TEM_-producing isolates, and four *bla*_TEM_- and *bla*_CMY-2_-producing isolates; also, one isolate possessed *qnr*A and produced *bla*_TEM_, *bla*_CTX-M_, and *bla*_OXA-10_. Meanwhile, the prevalence of *qnr*A positivity in buffalo-originated *P. mirabilis* isolates was significantly observed in one *bla*_TEM_- and *bla*_CMY-2_-producing isolate.

The frequency of *mcr-*1 positivity in broiler chicken-originated *P. mirabilis* isolates was significant among two *bla*_TEM_- and *bla*_CMY-2_-producing isolates and one *bla*_TEM_- and *bla*_CTX-M_-producing isolate; also, one isolate possessed *mcr-*1 and produced *bla*_TEM_, *bla*_CTX-M_, and *bla*_OXA-10_. Meanwhile, the prevalence of *mcr-*1 positivity in buffalo-originated *P. mirabilis* isolates was significantly detected in one *bla*_TEM_- and *bla*_CTX-M_-producing isolate; additionally, one isolate produced *mcr-*1 and possessed *bla*_TEM_.

The prevalence of *dfr*A1, *aad*A1, *sul*2, and *int*1 positivity in broiler chicken-derived *P. mirabilis* isolates among 13 *bla*_TEM_-producing isolates, 12 *bla*_TEM_- and *bla*_CMY-2_-producing isolates, 5 *bla*_TEM_- and *bla*_CTX-M_-producing isolates, 2 *bla*_TEM_-, *bla*_CTX-M_-, and *bla*_CMY-2_-producing isolates, 1 *bla*_TEM_-, *bla*_CTX-M_-, and *bla*_OXA-10_-producing isolate, and 1 *bla*_TEM_- and *bla*_CTX-M_-producing isolate was observed. But among buffalo-originated *P. mirabilis* isolates, the prevalence of *dfr*A1, *aad*A1, *sul*2, and *int*1 positivity was significant in five *bla*_TEM_-producing isolates, two *bla*_TEM_- and *bla*_CMY-2_-producing isolates, two *bla*_CTX-M_- and *bla*_CMY-2_-producing isolates, one *bla*_TEM_-, *bla*_OXA-10_-, and *bla*_CMY-2_-producing isolate, and one *bla*_TEM_-, *bla*_CTX-M_-, and *bla*_OXA-10_-producing isolate. Furthermore, the prevalence of *dfr*A1, *aad*A1, and *int*1 positivity in buffalo isolates was significant in one *bla*_TEM_- and *bla*_CTX-M_-producing isolate.

Furthermore, the occurrence of *erm*B positivity in broiler chicken-originated *P. mirabilis* isolates was valuable within four *bla*_TEM_- and *bla*_CMY-2_-producing isolates, three *bla*_TEM_-producing isolates, and one *bla*_TEM_- and *bla*_CTX-M_-producing isolate. Although the incidence of *erm*B positivity in buffalo-derived *P. mirabilis* isolates varied considerably, it was noted across one *bla*_TEM_-, *bla*_CTX-M_-, and *bla*_OXA-10_-producing isolate, and one *bla*_TEM_- and *bla*_CMY-2_-producing isolate.

In *P. mirabilis* isolates from broiler chickens, *tet*(M) positivity was shown to be prevalent in nine isolates that generated *bla*_TEM_, seven isolates that produced both *bla*_TEM_ and *bla*_CMY-2_, six isolates that produced *bla*_TEM_ and *bla*_CTX-M_, and one isolate that produced *bla*_TEM_, *bla*_CTX-M_, and *bla*_CMY-2_. However, a substantial rate of *tet*(M) positivity was observed in three *bla*_TEM_- and *bla*_CMY-2_-producing strains, two *bla*_CMY-2_-producing strains, one *bla*_TEM_-, *bla*_OXA-10_-, and *bla*_CMY-2_- producing strain, one *bla*_TEM_-, *bla*_CTX-M_-, and *bla*_OXA-10_-producing isolate, one *bla*_TEM_- and *bla*_CTX-M_-producing isolate, and one *bla*_TEM_-producing isolate in buffalo-originated *P. mirabilis* isolates.

Seven of the *P. mirabilis* isolates from broiler chickens had a high incidence of *cat*A1 positivity, which generated *bla*_TEM_ and *bla*_CMY-2_, four isolates produced *bla*_TEM_, three isolates produced *bla*_TEM_ and *bla*_CTX-M_, and one isolate produced *bla*_TEM_, *bla*_CTX-M_, and *bla*_OXA-10_. However, among the isolates of *P. mirabilis* isolated from buffalo, three isolates produced *bla*_TEM_ and *bla*_CMY-2_, one isolate produced *bla*_TEM_, one isolate produced *bla*_CMY-2_, one isolate produced *bla*_TEM_, *bla*_OXA-10_, and *bla*_CMY-2_, and one isolate produced *bla*_TEM_, *bla*_CTX-M_, and *bla*_OXA-10_.

### 3.5. Correlation and Concordance Between Genetic Determinants and Phenotypic Resistance Profiles

Dual analytical methodologies were employed to elucidate the relationship between genetic markers and observable resistance patterns. A correlation assessment (Figure 9) identified several significant gene–gene associations, with strong positive correlations between *sul*2 and *int*1 (r = 0.70) and *sul*2 and *bla*_TEM_ (r = 0.37). The *bla*_CMY-2_ gene demonstrated moderate correlations with multiple genes, including *bla*_CTX-M_ (r = 0.36) and *qnr*A (r = 0.36). Notably, *tet*(M) showed weak correlations with other resistance markers (r = 0.13–0.24), while *erm*B and *cat*A1 displayed limited associations with most genes in the network.

A concordance evaluation (Table 5) revealed substantial variation in the predictive accuracy across resistance determinants. High concordance rates were observed for *dfr*A1 (100% in both species), *aad*A1 (97.06–100%), and *sul*2 (90.91–97.06%). The *bla*_TEM_ gene showed strong but imperfect concordance (81.82–97.06%) with β-lactam resistance. Notably lower concordance was detected for *erm*B (18.18–23.53%) and *qnr*A, which exhibited striking host-specific disparity (47.06% in broilers vs. 9.09% in buffaloes).

Critical discordances emerged from our analysis: despite universal phenotypic resistance to several antibiotic classes, corresponding resistance genes showed variable detection rates. Particularly noteworthy was the complete absence of detectable carbapenemase genes despite observed meropenem resistance (79.41–100%). These findings suggest that while established genetic markers provide valuable predictive insights for certain resistance phenotypes, substantial gaps remain in our understanding of the full genetic basis of antimicrobial resistance in these populations.

### 3.6. Identification and Characterization of the Prepared CSNPs

**Figure 10:**
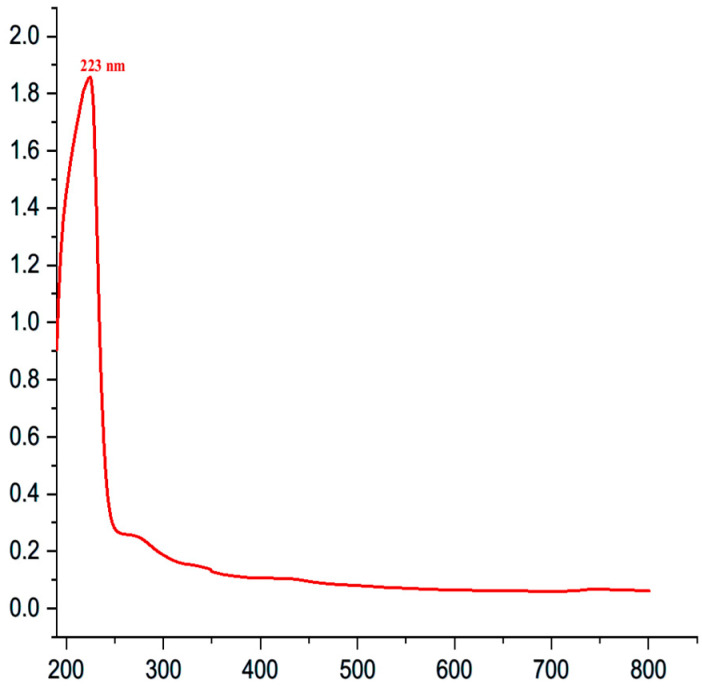
UV–Vis analysis of the synthesized CSNPs.

The UV–Vis spectrum of CSNPs is shown in Figure 10. In the present investigation, a peak of broad absorption band was observed at 223 nm, indicating that chitosan nanoparticles have been involved during the process. This revealed the presence of nanoparticle surface plasmon resonance (SPR), with a single SPR band indicating that the nanoparticles were spheres.

**Figure 11:**
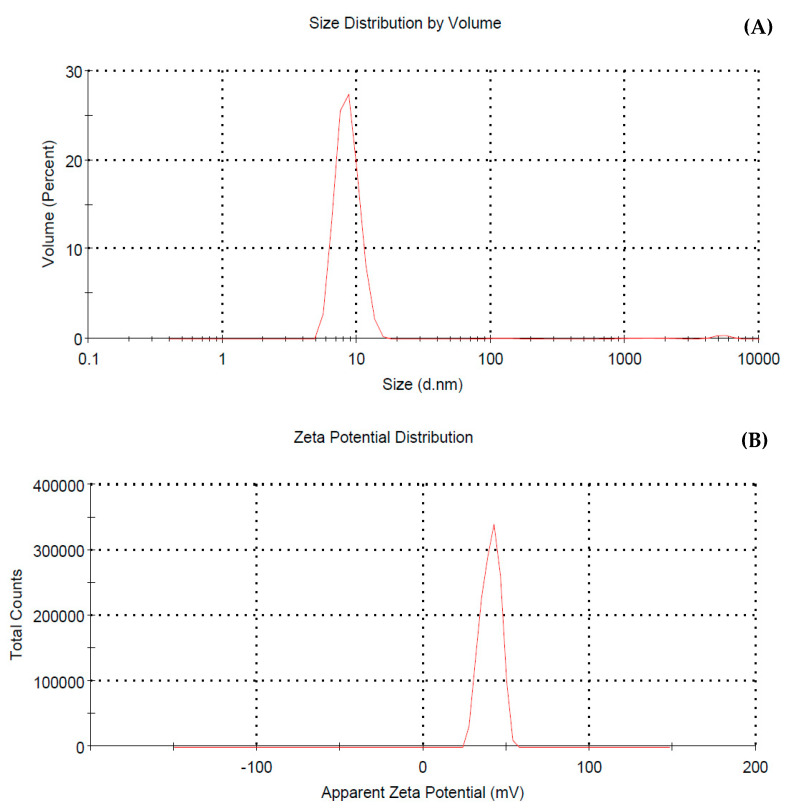
Size distribution (**A**) and zeta potential of the synthesized CSNPs (**B**).

The CSNP size was determined based on PSA readings, as the greatest intensity of Z-Average (d. nm) was 194.8 nm (Figure 11A) (Figure S13 at Supplementary File S2). The PDI was 0.973, and the volume-based distribution revealed three populations with the dominant peak (98%) at 8.66 nm. This size of CSNPs indicates that the nanoparticles were formed. Compared with the electron microscopy’s estimate, this size is larger. The mean zeta potential of the synthesized CSNPs was 40.3 mV with a standard deviation of 5.72 mV at 25 °C and 1.1 mS/cm conductivity, indicating a highly stable dispersion as the standard deviation remained low, reflecting minimal variation in particle surface charge across the analyzed samples (Figure 11B).

**Figure 12:**
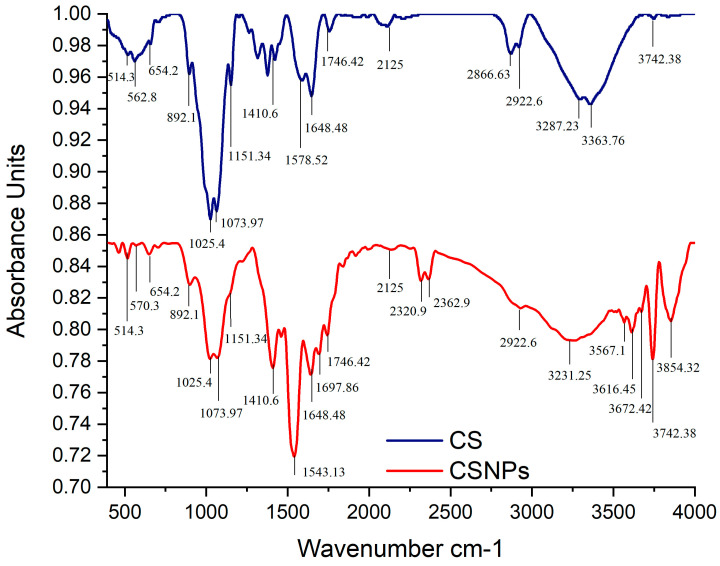
FTIR spectra of CS and CSNPs.

The FTIR spectra of pure CS and chitosan-TPP nanoparticles are compared in Figure 12 to confirm successful cross-linking via ionic gelation. The spectrum for CSNPs shows distinct changes compared with pure CS. The broad peak spanning 3854.32–3231.25 cm^−1^ in both spectra indicates strong hydrogen bonding from hydroxyl and amine groups. The signal at 2922.6 cm^−1^, present in both, is attributed to C-H stretching from the chitosan backbone. Minor peaks at 2362.9 and 2125 cm^−1^ might be due to absorbed ambient CO_2_ or leftover reagents. The peak at 1410.6 cm^−1^ showed the existence of chitosan by representing -CH_2_ bending and O-H deformation vibrations. Crucially, new prominent peaks appeared in the CSNPs spectrum at 1151.34 cm^−1^ and 1073.97 cm^−1^, which are assigned to P=O and P-O-C stretching vibrations, respectively. Peaks at 1025.4 and 892.1 cm^−1^ corresponded to C-O-C and C-O-H vibrations seen in polysaccharides, including chitosan. These peaks, which are absent in the pure CS spectrum, provide direct evidence for the successful incorporation of the TPP cross-linker. Furthermore, shifts and changes in the amide I (1648.48 cm^−1^) and amide II (1543.13 cm^−1^) bands in CSNPs, compared with CS, indicate enhanced polyelectrolyte complex formation. Additional evidence for the TPP interaction is seen in the P-O bending vibrations at 654.2, 562.8, and 514.3 cm^−1^ in the CSNP spectrum. This comparative analysis conclusively demonstrates functional group interactions between chitosan and TPP, confirming the successful synthesis of CSNPs.

**Figure 13:**
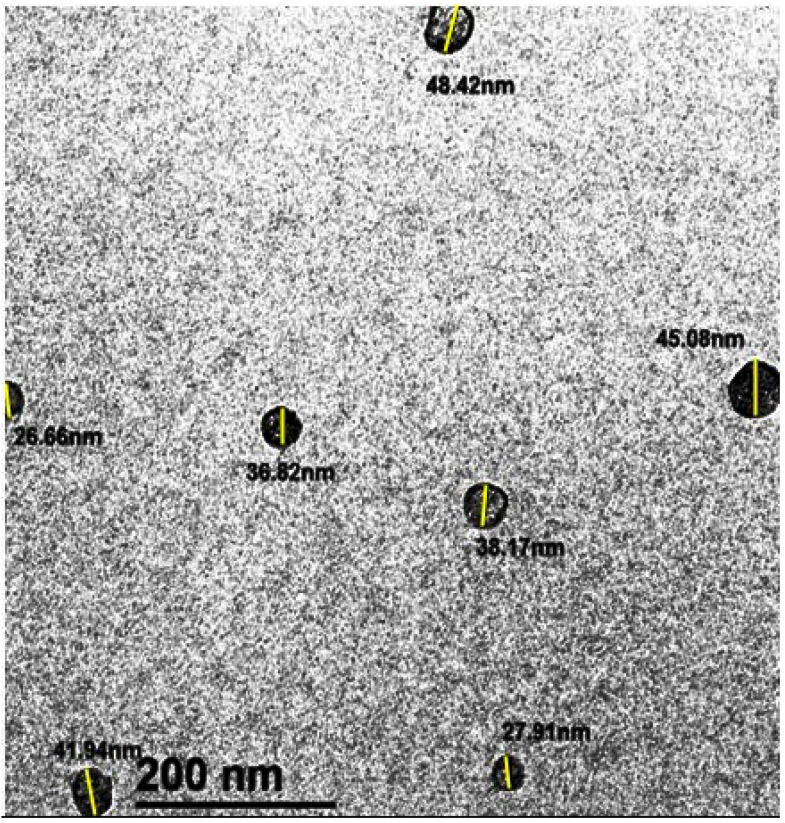
TEM image of CSNPs.

The surface appearance and size arrangement of the CSNPs were examined using TEM. The TEM image showed an essentially spherical morphology, a smooth surface, a tiny homogenous size distribution, and uniformity in appearance with a mean diameter of 37.18 ± 8.03 nm. After scaling the TEM picture to the determined scale bar value, a manual technique of measuring particle diameter (length) was used on the particles shown in Figure 13.

### 3.7. Antibacterial Efficacy of CS and CSNPs on P. mirabilis

The agar well diffusion test revealed significant antibacterial activity against *P. mirabilis* isolates from both broiler chickens (N = 34) and buffaloes (N = 11). The median inhibition zones for broiler isolates were 1.52 mm for CS 1%; 1.68 mm for CS 2%; 1.88 mm for CSNPs; and 2.0 mm for CIP. For buffalo isolates, the median zones were 1.6 mm, 1.73 mm, 1.9 mm, and 2.07 mm for the same treatments, respectively (Table 6). The combinations showed enhanced activity with median zones of 2.1 mm for CS 1% + CIP; 2.37 mm for CS 2% + CIP; and 2.45 mm for CSNPs + CIP in broiler isolates. In buffalo isolates, the combinations yielded 2.1 mm, 2.4 mm, and 2.6 mm for the same treatments, respectively. The enhancement was more evident when calculating the fold change in inhibition area relative to the CIP control (Table 6) (Table S1 at Supplementary File S3). In broiler isolates, the combinations showed a 1.1-fold (10% increase), 1.4-fold (40% increase), and 1.5-fold (50% increase) for CS 1%, CS 2%, and CSNPs combined with CIP, respectively. In buffalo isolates, the combinations demonstrated a 1.02-fold (2% increase), 1.4-fold (40% increase), and 1.58-fold (58% increase) enhancement for the same treatments. Statistical analysis using the Friedman test showed significant differences among treatments for both broiler (χ^2^ (6) = 61.9, *p* < 0.001) and buffalo (χ^2^ (6) = 61.9, *p* < 0.001) isolates. Post hoc Durbin–Conover’s test confirmed significant differences between all treatments and CIP control (all *p* < 0.05) (Table 7). The Mann–Whitney U test revealed no significant differences between broiler and buffalo isolates across all treatments (all *p* > 0.05). The CIP-CSNPs combination demonstrated the strongest antibacterial effect among all tested formulations, showing 50–58% enhancement in inhibition area compared with CIP alone.

### 3.8. Determination of MIC and MBC of CS 1%, CS 2%, and CSNPs Against Resistant P. mirabilis

**Figure 14:**
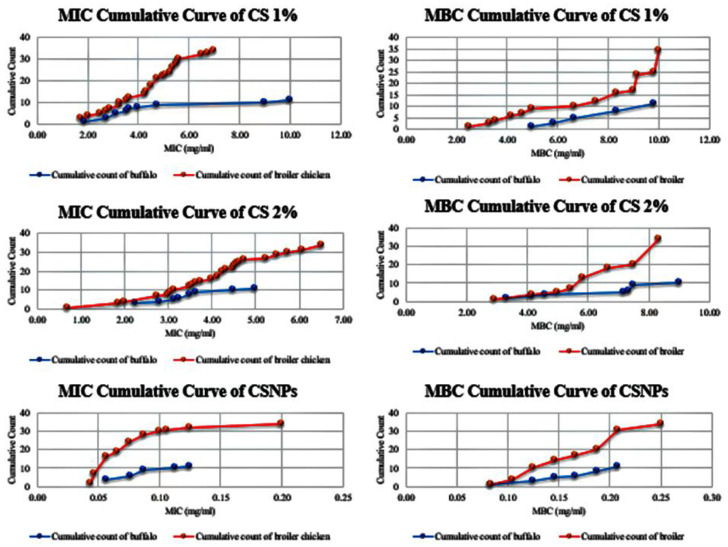
Cumulative curves of MIC and MBC values of CS 1%, CS 2%, and CSNPs showed a varying degree of inhibition with different concentrations against *P. mirabilis* strains collected from broiler chickens and buffaloes.

**Figure 15:**
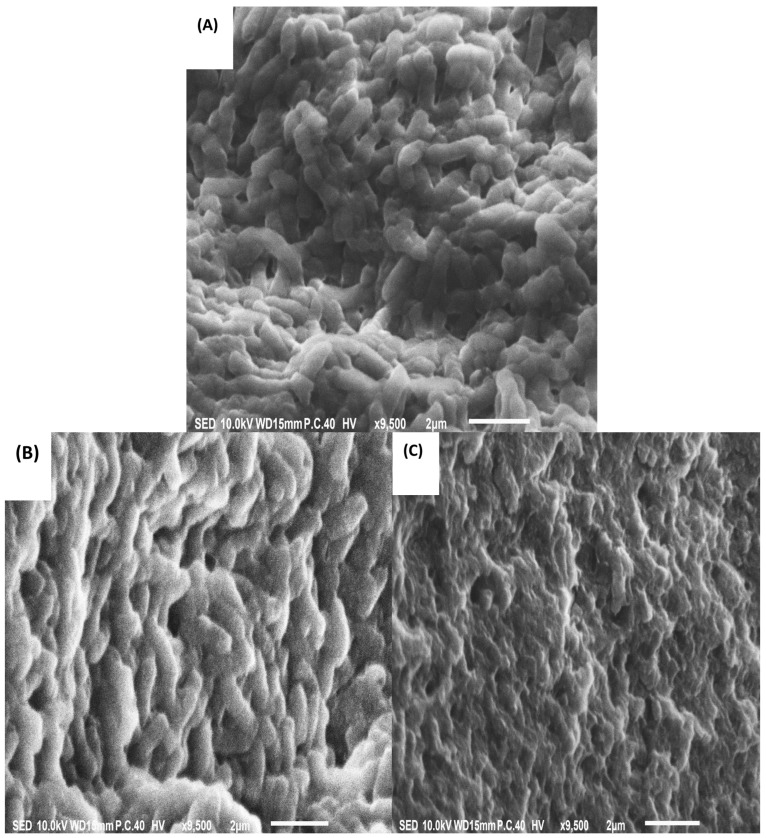
SEM micrographs of *P. mirabilis* cytomorphology before (control) (**A**) and after treatment with 0.156 mg/mL of CS (**B**) and with 0.0078 mg/mL CSNPs (**C**) after 18 h incubation.

The MIC and MBC values of CS formulations against *P. mirabilis* isolates revealed significant differences in their antibacterial efficacy. The median MIC values for broiler isolates were 4.5 mg/mL for CS 1%, 4.19 mg/mL for CS 2%, and 0.0672 mg/mL for CSNPs. For buffalo isolates, the median MIC values were 3.56 mg/mL, 3.25 mg/mL, and 0.0813 mg/mL for CS 1%, CS 2%, and CSNPs, respectively (Tables S2–S5 at Supplementary File S4). Similarly, the median MBC values for broiler isolates were 9.08 mg/mL for CS 1%, 6.67 mg/mL for CS 2%, and 0.177 mg/mL for CSNPs. For buffalo isolates, the median MBC values were 8.33 mg/mL, 7.33 mg/mL, and 0.167 mg/mL for CS 1%, CS 2%, and CSNPs, respectively (Tables S6–S9 at Supplementary File S5). A Mann–Whitney U test showed no significant differences in MIC or MBC values between broiler and buffalo isolates for any treatment (all *p* > 0.05) (Table 8). The determined MIC and MBC values displayed by CS and CSNPs against *P. mirabilis* are summarized in Table 9 and are illustrated in Figure 14. Post hoc pairwise comparisons revealed that CSNPs had significantly lower MIC and MBC values compared with both CS 1% and CS 2% (*p* < 0.001). While CS 2% showed lower MIC and MBC values than CS 1% in buffalo isolates (*p* = 0.001), this difference was not statistically significant in broiler isolates for MIC (*p* = 0.468), though it was significant for MBC (*p* = 0.003) (Table 9). Statistical analysis using the Friedman test showed significant differences among the treatments for both MIC and MBC in broiler and buffalo isolates (*p* < 0.001) (Table 10). Based on the investigation of the SEM micrographs (Figure 15), the reason CSNPs with a low conc. (0.0078 mg/mL) had more significant antibacterial activity than CS with a higher conc. (0.156 mg/mL) was presumably because of the tiny size and uniform appearance of the CSNPs. There were variations in how much antibacterial-inhibiting activity the CS and CSNPs had at the MIC and MBC against the tested bacterial strains at varied doses. Throughout the trial, the CSNPs showed powerful, consistent, and effective bactericidal effects against resistant *P. mirabilis*. The MBC/MIC index was greater than 1.5 for all formulations, indicating bactericidal properties. CSNPs demonstrated superior antibacterial activity at significantly lower concentrations compared with conventional chitosan, likely due to their nano-scale size and enhanced surface area. These findings suggest that CSNPs represent a promising biological alternative for preventing and treating *P. mirabilis* infections.

### 3.9. Scanning Electron Microscopy of P. mirabilis

As a consequence of the bactericidal activity of the CS and CSNPs, the shape of the examined bacterial cells had been distinguished from a control group after administering the bacterial cells with 0.156 mg/mL of CS and 0.0078 mg/mL of CSNPs for 18 h of incubation utilizing SEM. The cytomorphology of *P. mirabilis* control showed what seemed to be typical rods with a smooth, well-defined rigid surface (Figure 15A). While exposing *P. mirabilis* cells to CS (0.156 mg/mL) resulted in the most deteriorated cells, including deformed, expanded, and dented cell surfaces on the majority of the bacterial population, a few viable cells with a firm shape and smooth surface persisted (Figure 15B). The injected cells with CSNPs (0.0078 mg/mL) showed significant deformation with multiple dents and holes on the surfaces of the cells; also, practically all cell populations comprised deteriorated cells that seemed battered and wrinkly as well as shrunken (Figure 15C).

### 3.10. The Impact of CS and CSNPs on Swarming Motility

The findings revealed variations in swarming behavior among the isolates. Treatment with the sub-MICs of CS 2% and CSNPs significantly reduced swarming motility compared with the control. In broiler isolates, the median swarming diameter decreased from 4.27 cm (control) to 2.33 cm with CS 2% (45.4% reduction) and to 2.20 cm with CSNPs (48.4% reduction). Similarly, in buffalo isolates, the median diameter decreased from 4.27 cm (control) to 2.20 cm with CS 2% (48.4% reduction) and to 2.07 cm with CSNPs (51.5% reduction) (Table 11) (Table S10 at Supplementary File S6). Statistical analysis using the Friedman test confirmed a significant overall treatment effect (*p* < 0.001). Post hoc pairwise comparisons (Durbin–Conover’s test) revealed that both CS 2% and CSNPs significantly reduced swarming motility compared with the control (*p* < 0.001). Furthermore, CSNPs demonstrated a statistically superior inhibitory effect compared with CS 2% (*p* < 0.001) (Table 12). A Mann–Whitney U test showed no significant differences in swarming motility between broiler and buffalo isolates for any treatment group (all *p* > 0.05), indicating a consistent response across host species. These results suggest that the sub-MICs of CS and CSNPs may interfere with flagellar synthesis or rotation, thereby reducing the cellular motility activity of *P. mirabilis*.

## 4. Discussion

From a zoonotic point of view, *Proteus* spp. have been empirically associated with the bacterial contamination of chicken products, animal infections, and human illnesses [78]. The microbiological culture-based technique and molecular screenings have been utilized to efficiently detect 50 *Proteus* spp. isolates out of 450 samples at an incidence of 11.11% pooled from broiler chickens. El-Demerdash et al. [79] isolated *Proteus* spp. in 10% of broiler chicken samples, which is lower than our results. *P. mirabilis* isolates were observed in 34/50 (68%) broiler chickens, which is higher than previous results [39,80]. Additionally, 27 out of 270 (10%) pooled from native Egyptian buffalo samples were *Proteus* spp. strains. The recovery rate from buffalo samples was lower than the 39% achieved by Koirala et al. [81] and lower than the 17.64% achieved by Mansour et al. [82]. Our results revealed a higher incidence of *P. mirabilis* isolates from diseased broiler chickens (68%) than Egyptian native buffalo (40.74%). The incidence of recovered *P. mirabilis* from buffalo 11/27 (40.74%) was higher than the earlier result of Mansour et al. [82] (13.72%). This study’s *P. mirabilis* frequency varies from that of other studies, which could be explained by variations in collecting source, the type of samples, environmental issues, hygiene precautions performed, and sanitation factors among various communities’ chicken and buffalo farms, including inadequate airflow as well as overcrowding. As a result, a favorable environment for bacterial growth and colonization has been created.

The bulk of *P. mirabilis* isolates were previously sensitive to prevalent types of antibiotics; however, there have emerged numerous instances recently of antibiotic resistance rising [83], making infections more difficult to treat and posing a public health concern. Data from the present study indicated that chicken-originated *P. mirabilis* isolates had resistance against amoxicillin–clavulanic acid (100%), ampicillin–sulbactam (100%), trimethoprim–sulfamethoxazole (100%), gentamicin (100%), levofloxacin (91.18%), aztreonam (88.24%), chloramphenicol (100%), ciprofloxacin (91.18%), and meropenem (79.41%). In comparison, Li et al. (2022) [84] exhibited resistance rates of *P. mirabilis* to the above antibiotics of 34%, 40%, 98%, 75%, 96%, 0%, 98%, 98%, and 12%, respectively. While Ramatla et al. [85] reported resistances to amoxicillin–clavulanic acid, gentamicin, levofloxacin, ciprofloxacin, meropenem, and aztreonam as 46.2%, 34.6%, 53.8%, 61.5%, 11.5%, and 11.5%, respectively. The resistance to tetracycline and cefaclor was 100% and 94.12%, respectively. These results disagree with El-Saeed et al. [86], who reported 92.3% and 100%, respectively. Furthermore, a considerable resistance of 100% was detected against doxycycline and streptomycin. Contrary to Ma et al. [87], the resistance to doxycycline and streptomycin was 100% and 93%, respectively. In the present investigation, *P. mirabilis* isolates’ resistance to cefixime was 85.29%; this is in contrast to Yu et al. [88], who found 0%.

In the present study, buffalo-originated *P. mirabilis* isolates had resistance to amoxicillin–clavulanic acid, trimethoprim–sulfamethoxazole, gentamicin, aztreonam, chloramphenicol, ciprofloxacin, and meropenem as 100%. These results disagree with Sanches et al. [89], who reported 0%, 14%, 2%, 0%, 0%, and 6%, respectively. Furthermore, a considerable resistance of 100%, 100%, 100%, 100%, 100%, 90.91%, and 90.91% was detected against resistance to doxycycline, tetracycline, streptomycin, gentamicin, meropenem, ciprofloxacin, and levofloxacin, respectively. Contrary to Sun et al. [90], the resistance rates to the above antibiotics were 63.64%, 57.39%, 55.68%, 34.09%, 25%, 55.12%, and 25.57%, respectively. Also, Ma et al. [91] found 22.47%, 42.70%, 93.26%, 49.44%, 86.52%, and 94.38% resistance against amoxicillin– clavulanic acid, ciprofloxacin, streptomycin, gentamicin, chloramphenicol, and trimethoprim–sulfamethoxazole, respectively. Buffalo-derived *P. mirabilis* isolates were highly resistant to erythromycin (100%), in contrast to Ram et al. [92], who found 71.42%. Based on the findings, it is clear that the high antimicrobial resistance in broiler chickens and buffalo-originated *P. mirabilis* poses a public health concern.

ESBLs and ampC beta-lactamases are produced by members of the *Enterobacteriaceae* in food-producing animals [93]. As for ESBL, ampC, and resistance genes to other antibiotic families, including quinolones and aminoglycosides, they are more and more frequently identified in *P. mirabilis* [17]. The current study found that 33 out of 34 (97.06%) *P. mirabilis* samples from broiler chickens and 9 out of 11 (81.82%) samples from buffalo were identified as beta-lactamase-positive through genotyping, while all samples from both broiler chickens (100%) and buffalo (100%) were also classified as beta-lactamase producers based on their characteristics. In contrast to Hu et al. [94], the ESBL phenotype was confirmed in 47 chicken isolates (23.5%) and 3 in beef (3%). Also, Ramatla et al. (2024) [85] investigated eight (30.8%) broiler chicken isolates that were phenotypically classified as ESBL-producing, while 22 (84%) were classified genotypically. This agrees with the high prevalence detected for *bla*_TEM_ (97.06%) in broiler chickens and 81.82% in buffalo. The *bla*_TEM_ gene is responsible for more than 80% of the resistance in enteric pathogens [95]. Contrary to the percentage of *bla*_TEM_ in broiler chicken isolates of the present study, Almeida et al. [96] detected *bla*_TEM_ as 100%. In contrast to the high prevalence of *bla*_TEM_, we detected no positive isolates in both species for *bla*_SHV_. Similarly, Zhu et al. [80] did not detect *bla*_SHV_ in their isolates, although Li et al. [84] detected *bla*_SHV_ in 4% of broiler-originated *P. mirabilis* isolates. It was observed that the *bla*_CTX-M_ gene was 26.47% in broiler chickens’ isolates and 18.18% in buffalo isolates. In contrast, Zhu et al. [80] did not detect *bla*_CTX-M_ in their isolates. This is contrary to Sarwar et al. [97], who detected *bla*_CTX-M_ (49%) and *bla*_TEM_ (25.67%), and Ramatla et al. [85], who detected *bla*_CTX-M_ (19.2%) and *bla*_TEM_ (15.4%) in poultry. In contrast with what was discovered in this specific investigation, Ejaz et al. [98] detected the prevalence of *bla*_CTX-M_, *bla*_TEM_, and *bla*_SHV_ in both broiler chickens and buffalo *P. mirabilis* isolates, which were 66.7%, 16.7%, and 16.7%, respectively. AmpC β-lactamase-producing strains have become the leading source of nosocomial disease epidemics along with therapeutic failures [99]. In this study, 41.18% and 63.64% of isolates harbored the (ampC) *bla*_CMY-2_ gene in broiler chickens and buffalo, respectively. According to Sanches et al. [89], *bla*_CMY-2_ had been identified in 13% and 0% of the chicken isolates and the cattle isolates, respectively. The present study detected 41.18% *bla*_CMY-2_ and 2.94% *bla*_OXA-10_ genes in chicken-originated *P. mirabilis*, which is higher than Ma et al. [91], who reported 3% and 0.5%, respectively.

Detecting CPE is especially problematic in *Proteus* spp., which have little carbapenem resistance while manufacturing a carbapenemase [100]. This result is consistent with our findings, which showed that the prevalence of the *bla*_KPC_, *bla*_GES_, *bla*_VIM_, *bla*_IMP_, *bla*_NDM-1_, and *bla*_OXA-48_ genes was 0. Mushi et al. [101] found no *bla*_KPC_, *bla*_GES_, *bla*_VIM_, *bla*_NDM-1_, or *bla*_OXA-48_ in their isolates. In addition, Zhu et al. [80] found no carbapenem resistance genes in broiler chicken isolates from China, including *bla*_KPC_, *bla*_IMP_, *bla*_VIM_, and *bla*_OXA-48_. Furthermore, Eltaweel et al. [102] identified *bla*_OXA-48_ (10.6%) and *bla*_NDM-1_ (7.6%), but not *bla*_KPC_, *bla*_VIM-1_, or *bla*_IMP_ genes. The results of this investigation show that *P. mirabilis* isolates differ significantly in their carbapenem resistance between genotype and phenotype. Even though all isolates showed high levels of resistance to the carbapenems meropenem (100% and 79.41%) and aztreonam (100% and 88.24%), none of them tested positive for the common carbapenemase genes (*bla*_KPC_, *bla*_OXA-48_, *bla*_VIM_, *bla*_IMP_, *bla*_GES_, *bla*_NDM-1_, and *bla*_SHV_). However, genetic investigation indicated a significant frequency of ESBL and ampC genes such as *bla*_TEM_, *bla*_CTX-M_, *bla*_OXA-10_, and *bla*_CMY-2_. This surprising observation clearly demonstrates that non-carbapenemase pathways, in combination with ESBLs/ampC, transmit carbapenem resistance in these isolates. The most probable reason is the combination of ESBL/ampC overproduction and decreased outer membrane permeability caused by porin depletion or mutations. This combinatorial effect greatly restricts carbapenem entrance into the bacterial cell, permitting existing ESBLs/ampC to inactivate the limited antibiotic concentration that does enter, resulting in the observed high level of phenotypic resistance. These findings emphasize the complexities of antimicrobial resistance and the crucial need for extensive research beyond carbapenemase gene screening to completely understand resistance mechanisms.

A very intriguing finding in our study was the detection of *int*1 in 97.06% of broiler chicken isolates and 100% of buffalo isolates. This is in comparison to Ejaz et al. [98], who detected 84.5% *int*1 of the ESBL producers in both broiler and buffalo isolates. Also, Ramatla et al. [85] detected (*int*1) in 42% of broiler chicken isolates. The incidence rate of *mcr-*1 was 11.76% (4/34) and 18.18% (2/11) from broiler isolates and buffalo isolates, respectively. In comparison, Ma et al. [87] and Almeida et al. [96] reported that the prevalence of *mcr-*1 was found in 34.76% and 71.4% of *P. mirabilis* isolated from chickens, respectively. The misuse of colistin in food-producing animals has caused increased levels of resistance to colistin in enteric bacteria [103]. Finding the underlying etiology, mode of distribution, and progression of colistin-resistant diseases is therefore becoming more and more important on a worldwide scale.

The quinolone *qnr*A gene is exceedingly uncommon in *P. mirabilis* [17]. However, in the present study, the percentages of quinolone-resistant gene *qnr*A and *qnr*S were 47.06% (16/34) and 0% (0/34) in broiler chicken-originated *P. mirabilis* isolates and 9.09% (1/11) and 0% (0/11) in buffalo-originated *P. mirabilis* isolates. The proportion of these outcomes is lower than that of Ramatla et al. [85], who found 50% of isolates containing *qnr*A in poultry. The prevalence of *dfr*A1, *sul*2, *aad*A1, *cat*A1, *msr*A, *gyr*A, and *fos*A was 100%, 97.06%, 97.06%, 44.12%, 0%, 0%, and 0%, respectively, detected in chicken-originated *P. mirabilis* isolates. Meanwhile, Hu et al. [94] exhibited resistance rates of chicken-originated *P. mirabilis* to *dfr*A1, *sul*2, and *aad*A1 as 27.5%, 78.3%, and 27.5%, respectively, whereas the prevalence of *sul*2 detected in buffalo isolates was 90.91%. Also, Sanches et al. [89] reported the prevalence of *sul*2 (62%) in broiler chickens and (13%) in cattle. Fosfomycin has been receiving renewed attention as a therapy for severe systemic infections brought on by *Enterobacteriaceae* that are resistant to many drugs [104]. Given its low cost and effectiveness as a carbapenem-avoiding strategy, the WHO has categorized fosfomycin as a “critically important” antibiotic that has high potential for treating MDR Gram-negative infections globally [105]. The frequency of *fos*A was 0% in both species’ isolates, which is consistent with the findings of Lalezadeh et al. [106], who did not detect the *fos*A gene. The present study detected 81.82% *tet*(M) and 23.53% *erm*B genes in chicken-originated *P. mirabilis*, which is lower than the findings of Almeida et al. [96], which were 100%. It shows that the genotypic method, which uses the precise PCR amplification of resistance genes, is still very accurate and reliable. The significance of molecular technologies in evaluating antibiotic resistance profiles is highlighted by the notable distinction between genotypic and phenotypic approaches. The phenotypic approach has a lesser sensitivity for detecting resistance, and environmental variables influence the occurrence of resistance [107].

According to the findings of the present research, it is worth noting that strains displayed a higher XDR (50%) and PDR (50%) level in broiler chicken-originated *P. mirabilis* isolates and XDR (9.09%) and PDR (90.91%) level in buffalo-originated *P. mirabilis* isolates than in studies performed by Hu et al. [94], which investigated the prevalence of MDR (76.5%) in chicken meat and (6%) in beef. Sun et al. [90] noted that approximately 76.7% of the strains exhibited MDR or XDR. Also, Ma et al. [91] determined that 91.01% displayed an MDR profile. Contrary to one of the earliest reports of poultry-originated *P. mirabilis* in Egypt, this investigation found that 22.8% of isolates were MDR, 31.4% were XDR, and 8.5% were PDR [108]. This high prevalence of MDR and PDR in foodborne pathogens is a cause for concern, especially considering the possibility of cross-contamination throughout the food chain, posing a risk for human and animal health. Antimicrobial agent abuse and overuse on veterinary farms are indicated by a MAR index greater than 0.2 [109]. Interestingly, it was noticed that 100% of *P. mirabilis* isolates have an MAR index over 0.2. The antibiograms produced by the current investigation are distinct from those of other studies, demonstrating how the antibiotic pattern varies depending on the isolate, time, and XDR/PDR generation within *P. mirabilis* isolates.

The drivers underlying the observed resistance profiles and genotype–phenotype discrepancies warrant thorough investigation. The remarkably high resistance rates detected in this study, particularly the universal resistance (100%) to multiple antibiotic classes in both broiler and buffalo isolates, highlight a critical public health concern. While direct comparisons across studies require methodological caution, the consistently elevated resistance patterns suggest region-specific selective pressures. A principal contributing factor is likely the extensive and often unregulated application of antimicrobials in animal husbandry within the study area, creating sustained selective environments that promote the persistence and dissemination of resistant bacteria. Moreover, the diverse resistance gene constellations identified—evidenced by the variable distribution of *bla*_TEM_, *sul*2, *aad*A1, *tet*(M), and the contrasting *qnr*A prevalence between hosts—indicate that resistance dissemination is not attributable to clonal expansion alone. Instead, the data strongly implicate horizontal gene transfer mediated by mobile genetic elements. This is supported by a correlation analysis revealing significant gene–gene associations (e.g., *sul*2-*int*1: r = 0.70; *bla*_TEM_-*int*1: r = 0.56), suggesting potential co-localization on transferable platforms. A key study limitation is the targeted nature of the genetic screening, which did not incorporate molecular typing techniques such as whole-genome sequencing to conclusively differentiate clonal spread from horizontal gene transfer events. Future research employing high-resolution molecular epidemiology is therefore essential to validate these proposed mechanisms and delineate precise resistance transmission routes within this ecosystem.

The analysis further elucidates the complex interplay between detectable resistance genes and observed phenotypic resistance. While high concordance was noted for several markers (e.g., *dfr*A1: 100%; *aad*A1: 97.06–100% with streptomycin resistance), significant and critical discordances emerged. The most notable finding was the detection of widespread phenotypic resistance without corresponding genetic determinants. This pattern of unexplained phenotypic resistance was particularly evident for furazolidone, metronidazole, and rifampin, where universal resistance (100%) was observed despite the absence of targeted genetic screening for corresponding resistance mechanisms. This also includes the universal resistance to gentamicin (100%) in both species, which lacked the detection of corresponding aminoglycoside resistance genes such as *aac(6’)-Ib*. Similarly, the high-level resistance to macrolides was poorly explained by the screened genetic markers, with low detection of the *erm*B gene (18.18–23.53%) and a complete absence of *msr*A, suggesting the potential role of other erm gene variants or efflux systems. Furthermore, the high-level resistance to carbapenems (79.41–100%) occurred in the complete absence of the targeted carbapenemase genes (*bla*_KPC_, *bla*_GES_, *bla*_VIM_, *bla*_IMP_, *bla*_NDM-1_, *bla*_OXA-48_), and resistance to fluoroquinolones showed low concordance with *qnr*A (9.09–47.06%) and no detection of *qnr*S or *gyr*A mutations. Additionally, the fosfomycin resistance gene *fos*A was not detected, though phenotypic testing for fosfomycin was not performed. These discrepancies imply the contribution of alternative resistance mechanisms, such as efflux pumps, undetected or novel resistance genes, or chromosomal mutations. Conversely, the detection of genes like *bla*_CTX-M_ and *bla*_OXA-10_ in isolates susceptible to certain β-lactams may indicate unexpressed or silent genetic reservoirs. These observations underscore the inherent limitations of targeted PCR-based approaches and emphasize the multifactorial basis of antimicrobial resistance in these populations, necessitating integrated methodologies that combine comprehensive genotypic profiling (e.g., whole-genome sequencing) with phenotypic susceptibility testing for accurate resistance characterization.

The outcomes indicated the creation of freshly manufactured CSNPs at the highest peak of 223 nm, indicating nanoparticles in an excited phase from a grounded to an excited state, as illustrated by Little et al. [110]. These results match the study by Vaezifar et al. [111], in which the UV–visible spectra showed an absorption peak at 226 nm. The average hydrodynamic diameter of CSNPs measured by DLS was found to be 194.8 nm. While the Z-average reflects an intensity-weighted mean influenced by larger aggregates, the volume distribution shows the primary nanoparticle population (98%) at 8.66 nm, consistent with TEM observations. This size of CSNPs suggests that the nanoparticles were generated. Notably, this size is larger than that estimated by electron microscopy, due to the high swelling ability of chitosan nanoparticles. Similarly, Essa et al. [112] detected that the size of CSNPs using DLS was 477 nm, while using TEM it was 200–280 nm. DLS measures the particle’s hydrodynamic radius, while TEM estimates the projected area diameter. When a dispersed particle in DLS passes through a liquid medium, a small electric dipole layer of the solvent adheres to its surface, as this layer has an effect on particle movement [112]. Furthermore, CSNP size measurement using zeta size yielded data similar to those reported in a previous study [113] (210 nm). The zeta potential of chitosan nanoparticles was found to be 41.77 mV, and this value aligns with the 40 mV obtained by Loutfy et al. [113]. The ZP is an important element in ensuring the stability of nanoparticles in a suspended state, as a value greater than ±20 mV indicates a strongly positively charged surface on the nanoparticles, giving a high stability and lowering the chances of aggregation [114]. The synthetic chitosan in this work was effectively produced, according to the FTIR spectrum data. Throughout this specific study, the FTIR analysis of CSNPs highlighted the band spectrums of different bonds, which is compatible with Kulig et al. [115] and Kadhum and Zaidan [116]. TEM revealed that CSNPs exist as homogeneous tiny spheres, which agrees with Chandrasekaran et al. [117]. However, Loutfy et al. [113] generated larger CSNPs using TEM than those used in the current research, with a size of 150 nm. The size of CSNPs in the current investigation differed from that in other studies, which might be attributable to diverse sources of CSNP manufacturing. The potent antibacterial activity of the synthesized CSNPs can be attributed to a synergistic combination of their small primary particle size (8.66 nm), highly positive surface charge (+41.77 mV), and the intrinsic polycationic nature of chitosan. While the small size enables enhanced cellular penetration and large surface area for interaction, the strong positive charge promotes effective electrostatic binding to negatively charged bacterial membranes, leading to membrane disruption and improved antibacterial efficacy.

A high MAR index, from our viewpoint, constitutes a significant issue, especially when facing serious zoonotic diseases, including *P. mirabilis*, that have become resistant to the significant majority of critically important antibiotics prescribed for livestock and humans. So, first of all, appropriate antibiotic usage and the creation of scientific monitoring systems are the most effective approaches for reducing the harmful effects of antibiotic overuse while also ensuring the safety of animal-derived food. In light of rising antibiotic resistance concerns in the veterinary business, this highlights the critical necessity of replacing antibiotics with a naturally potent occurring antimicrobial to combat bacterial resistance for successful therapy in livestock and humans. As in this study, CS, CSNPs, and the combination separately with CIP against resistant *P. mirabilis* were investigated. In fact, the World Health Organization identified CIP as a critically essential antibiotic [46]; hence, it was employed in the current investigation and loaded with CS and CSNPs to increase drug delivery and therapeutic effectiveness. By contrasting CIP with CSNPs, the nanoparticles demonstrated enhanced antibacterial activity, with the CSNPs + CIP combination showing the highest median inhibition zones (2.45 mm in broilers and 2.6 mm in buffalo) representing a 50–58% increase in inhibition area compared with CIP alone. The combinations of CS with CIP also showed significant enhancement, with CS 2% + CIP increasing inhibition area by 40% in both animal species, and CS 1%+CIP showing a 10% and 2% increase in broilers and buffaloes, respectively. This significant enhancement in the inhibition zone diameter following nanoformulation is strongly supported by a previous study on trimethoprim, where the mean inhibition zone of the nanoemulsion (41.5 ± 1.2 mm) was more than double that of the pure drug (19.8 ± 1.5 mm) [118]. Furthermore, the enhanced inhibitory effect of nanoformulations is consistent with findings from a well diffusion assay evaluating curcumin-AgNPs. The zone of inhibition for the synergistic curcumin-AgNP composite ranged from 15.03 ± 0.15 mm to 19.10 ± 0.11 mm, which was substantially larger than the zone of inhibition of its individual components (AgNPs: 10.86 ± 0.11 mm to 12.03 ± 0.25 mm; curcumin: 11.13± 0.15 mm) [119]. When contextualizing our CSNPs’ performance against other nanomaterials, a comprehensive comparison reveals their competitive advantage. The largest zone of inhibition for AgNPs and CuNPs against *P. mirabilis* was reported at a concentration of 0.4 μg/μL, with the bacteria showing resistance at 0.1 μg/μL [120]. In contrast, our CSNPs achieved significant antibacterial efficacy, as demonstrated by the large inhibition zones, at concentrations comparable to or lower than those reported for other NPs. Similarly, another study on green-synthesized nanoparticles found that ZnNPs (200 μg/disk) showed an inhibition zone of 12 ± 1.2 mm against *P. mirabilis*, followed by ZnSeNPs (11 ± 1.2 mm), while Se NPs exhibited the weakest activity (9 ± 1.0 mm) [121]. Research on selenium-based nanocomposites further revealed that Se/CS/AMP exhibited an inhibition zone of 10 ± 0.14 mm against *P. mirabilis*, which was superior to Se NPs alone (6 ± 0.22 mm) [122]. This demonstrates that while various nanoparticles show activity against *P. mirabilis*, our CSNPs demonstrate highly competitive potency. This competitive edge is further evidenced by the MIC and MBC results. Furthermore, the synergistic effect observed in our study between CSNPs and CIP finds a parallel in the reported agonistic activity between AgNPs and plant extracts [120], underscoring that the enhanced efficacy of nanocomposites is a reliable phenomenon in combating *P. mirabilis*. As a result, it promotes using CIP-loaded CS or CSNPs in treating enteric bacterial infections. Indeed, nano-sized chitosan promotes medication absorption across the cell membrane. The additional benefit of loading CIP with CSNPs is that it allows for more variable routes of delivery, especially non-invasive routes including the oral, nasal, and ocular mucosa, that are desired.

As determined by the present study, the median MIC and MBC values of CS 1% against isolates of *P. mirabilis* in buffalo were 3.56 mg/mL and 8.33 mg/mL, and in broiler chickens were 4.5 mg/mL and 9.08 mg/mL, respectively. For CS 2%, the median MIC and MBC values were 3.25 mg/mL and 7.33 mg/mL for buffalo, and 4.19 mg/mL and 6.67 mg/mL for broiler chickens, respectively. The median MIC and MBC values of CSNPs against broiler chicken-originated *P. mirabilis* were found to be 0.0672 mg/mL and 0.177 mg/mL, respectively. The median MIC and MBC values of CSNPs against buffalo-originated *P. mirabilis* were found to be 0.0813 mg/mL and 0.167 mg/mL, respectively. Furthermore, when examining the relationship between the degree of antibiotic resistance and the efficacy of our CSNPs, a critical pattern emerges. All tested *P. mirabilis* isolates were classified as either XDR or PDR, representing the highest conceivable level of conventional antibiotic resistance. Despite this extreme resistance profile, our CSNPs exhibited consistently potent and uniform antibacterial activity against all isolates, as evidenced by the remarkably low and stable MIC values. This consistency was observed across isolates from different hosts (broiler chickens and buffaloes) possessing diverse arrays of resistance genes (e.g., *bla*_TEM_, *mcr-*1, *qnr*A). The finding that CSNPs were equally effective against all strains, irrespective of their specific resistance mechanisms or their PDR/XDR status, strongly suggests a lack of direct correlation between conventional antibiotic resistance and susceptibility to CSNPs. This can be attributed to the fundamental, multi-targeted mechanism of action of CSNPs, primarily involving electrostatic attraction and the physical disruption of the bacterial cell membrane, which effectively bypasses the specific enzymatic and target-based resistance pathways that define XDR and PDR strains.

In contrast, Kadhum and Zaidan [116] showed that chitosan alginate nanoparticles caused a 100% inhibition rate to *P. mirabilis* at 0.15 and 0.3 mg/mL, while the study by Hussein and Aldujaili [123] showed that 80 mg/mL of CSNPs exhibited a large inhibition rate to *P. mirabilis* growth and a lower inhibition rate at 20 and 10 mg/mL. The remarkable reduction in MIC and MBC values observed with our CSNPs aligns with findings from other nanoformulations. For instance, a trimethoprim nanoemulsion showed an eight-fold reduction in MIC (0.5 µg/mL compared with 4 µg/mL for the pure drug) and an eight-fold reduction in MBC (2 µg/mL compared with 16 µg/mL for the pure drug) against *P. mirabilis* [118]. The potency of our CSNPs becomes even more notable when compared to selenium-based nanocomposites [122]. While Se/CS/AMP showed enhanced activity with an MIC of 100 μg/mL (0.1 mg/mL) against *P. mirabilis* compared with Se NPs alone (150 μg/mL or 0.15 mg/mL), our CSNPs achieved significantly lower MIC values (0.0672–0.0813 mg/mL). This represents an approximately 1.5- to 2.2-fold improvement in potency over the Se/CS/AMP nanocomposite. More importantly, the superior efficacy of our CSNPs is highlighted when compared to other metallic nanocomposites [120]. The MIC values for our CSNPs are dramatically lower than the effective concentrations reported for combinations of silver or copper NPs with plant extracts (which required inhibitory concentrations as high as 250–500 μL). The exceptional performance of our CSNPs is further solidified when compared to the efficacy of curcumin–silver nanocomposites (Cur-AgNPs) reported in a separate study [119]. While Cur-AgNPs demonstrated enhanced antibacterial activity with MIC values ranging from 0.024 to 0.049 mg/mL, our CSNPs achieved comparable MIC values (0.0672–0.0813 mg/mL) without the need for a secondary antimicrobial agent like curcumin or silver. Furthermore, the MBC values of our CSNPs (0.167–0.177 mg/mL) were lower than those reported for AgNPs alone (0.195–0.780 mg/mL) and significantly lower than those of curcumin (3.125–12.500 mg/mL). This stark contrast not only underscores the exceptional potency of CSNPs but also suggests a more efficient mechanism of action compared with other tested nanocomposites. Based on the findings, it can be concluded that the degree of growth inhibition of CS and CSNPs can effectively combat the development of PDR *P. mirabilis* as concentrations rise. The number of isolates examined, the synthesis process, the synthesis circumstances in the lab, and the properties of the nanoparticles might all have contributed to the discrepancies between this inquiry and the others [124]. The bactericidal action was much more pronounced in the case of *P. mirabilis*. In contrast, Kadhum and Zaidan [116] observed that chitosan alginate nanoparticles were bacteriostatic against *P. mirabilis*. In the current study, it was discovered that CSNPs have superior antibacterial efficacy to chitosan because nano-chitosan is smaller in size, has a larger surface area, and has a stronger attraction to bacterial cells, which is certainly responsible for its higher antibacterial activity [75]. Also, these findings agree with Chandrasekaran et al. [117], who discovered that CSNPs had stronger antibacterial activity than chitosan and chitin.

The swarming movement was responsible for combining sensory transmission with generalized controlling actions. The significant inhibition of *P. mirabilis* swarming motility observed in this study highlights the potential of chitosan-based formulations as anti-virulence agents. This coordinated movement, crucial for bacterial colonization, was effectively disrupted. Treatment with CSNPs at concentrations of 0.0313 and 0.0625 mg/mL reduced the mean swarming diameter from 4.27 to 2.20 cm (approximately 48.4% reduction) and from 4.27 to 2.07 cm (approximately 51.5% reduction) in broiler and buffalo isolates, respectively. Similarly, CS at 2.5 mg/mL reduced motility from 4.27 to 2.33 cm (approximately 45.4% reduction) in broilers and from 4.27 to 2.2 cm (approximately 48.4% reduction) in buffalo isolates. The notable efficacy of CSNPs, even at lower concentrations, can be attributed to their enhanced permeability and larger surface area, which facilitate greater interaction with bacterial cell membranes and flagellar components. This attenuation of a key virulence mechanism not only impedes biofilm formation and colonization but may also restore susceptibility to conventional antibiotics by disrupting the pathogen’s ability to spread and establish infections. These findings align with emerging anti-virulence strategies that aim to disarm pathogens without exerting direct lethal pressure, thereby potentially reducing the development of resistance. In contrast to Aljobori and Al-Rawi [125], it was revealed that the mean diameter of swarming motility in non-treated cultures was 8.5 cm, whereas in the presence of CSNPs, it was decreased to 0.5 cm, assessed at a concentration of 5 mg/mL, and the rate of swarming motility inhibition was 94%. *P. mirabilis* swarming variations can be influenced by a variety of factors, including strain variation, growth circumstances, their source, and incubating conditions, comprising the medium, pH, temperature, moisture, and the expression of specific swarming genes.

## 5. Conclusions

The highest contamination was recorded in broiler chickens (68%), followed by buffaloes (40.74%), with a possible risk of a potential threat to food safety and cross-contamination between poultry, cattle, and humans. The high MAR index (>0.2) in all isolates unequivocally indicates excessive antibiotic use in these settings. The data highlight the worrying spread of XDR and PDR *P. mirabilis* to the majority of routinely used antimicrobial drugs in Egypt. This study also detected four beta-lactamase genes, *bla*_CTX-M_, *bla*_TEM_, *bla*_OXA-10_, and ampC (*bla*_CMY-2_), alongside other resistant genes (*sul*2, *aad*A1, and *dfr*A1) and the high prevalence of the class 1 integron (*int*1) suggesting horizontal gene transfer. A pivotal finding was the mechanistic explanation for carbapenem resistance despite the absence of carbapenemase genes, attributable to ESBL/ampC overproduction combined with reduced membrane permeability. Therefore, these findings underscore the urgent need for the concerned health authorities to undertake strict measures and enforce management for the dissemination of PDR *P. mirabilis* in food-producing animals and associated environments to safeguard the public from the spread of antimicrobial-resistant bacteria to humans. So, CS and CSNPs with optimal properties (small size ~8.66 nm, positive charge +41.77 mV) were investigated in vitro to aid in the reduction in *P. mirabilis* resistance. The outcomes demonstrated that CSNPs-CIP were more successful against resistant *P. mirabilis* than either native CIP or the enhancement impact of CS-CIP, increasing the inhibition zone area by 50–58%. The key result of the current research is that the CS and CSNPs were appropriate for the treatment of resistant *P. mirabilis* infections in vitro, with the nanoparticles having a powerful impact, especially at low doses of the CSNPs-CIP. Furthermore, CSNPs exhibited significant anti-virulence activity, reducing swarming motility by approximately 48–51%. When treating harmful *Proteus* infections, we advise using these natural remedies either by themselves or in combination with ciprofloxacin to ensure bactericidal/bacteriostatic efficiency. In light of these critical findings, we strongly recommend an integrated dual strategy to address the escalating public health threat. First, the immediate implementation of rigorous antimicrobial surveillance and stewardship programs in veterinary practices is essential to curb the dissemination of PDR *P. mirabilis*. Second, we advocate for the clinical adoption of chitosan-based nanoformulations, particularly CSNPs either alone or in synergistic combination with conventional antibiotics, as innovative and sustainable therapeutic interventions. This comprehensive One Health approach—bridging veterinary and human medicine—is crucial to mitigate the transmission of resistant pathogens across the human–animal–environment interface and safeguard the efficacy of existing antimicrobials.

## 6. Study Limitations and Future Directions

This study, while providing insights into antimicrobial resistance patterns and the efficacy of chitosan-based combinations, has certain limitations that should be acknowledged. The principal constraint lies in the observed discordance between phenotypic resistance and the genotypic profiles identified through targeted PCR. Widespread resistance to multiple antimicrobial classes—including carbapenems, fluoroquinolones, aminoglycosides, furazolidone, metronidazole, and rifampin—lacked corresponding genetic determinants, suggesting the involvement of alternative mechanisms such as efflux pumps, undetected genes, or chromosomal mutations. Furthermore, the scope of this work did not encompass essential assessments such as quantitative synergy measurements (e.g., checkerboard FIC index), time–kill kinetics, cytotoxicity evaluations, or in vivo models, which are crucial for fully characterizing the therapeutic potential and safety of the investigated antibacterial agents.

To address these gaps, future research should adopt more integrated methodologies. Whole-genome sequencing is recommended to comprehensively elucidate the genetic basis of resistance and uncover novel mechanisms. Additionally, expanded phenotypic susceptibility testing, including for agents such as colistin and fosfomycin, is warranted. Most importantly, subsequent studies should incorporate checkerboard assays, time–kill kinetics, cytotoxicity assessments, and in vivo experiments to validate interactions, dose–response relationships, and biosafety profiles, thereby providing a more robust foundation for translational applications.

## Figures and Tables

**Table 1:** Antimicrobial agents used for susceptibility testing according to WHO classification.

WHO Classification	Antimicrobial Group	Antimicrobial (Full Name)	Abbreviation	Disk Content (µg)
Critically important	Penicillins	Amoxicillin	AMX	10
	Carbapenem	Meropenem	MEM	10
	Macrolides	Clarithromycin	CL	15
		Erythromycin	E	15
	Aminoglycosides	Gentamicin	GEN	10
		Streptomycin	HLS	300
	Fluoroquinolones	Levofloxacin	LEV	5
		Ciprofloxacin	CIP	5
	Rifampicin	Rifampin	RD	5
	Cephalosporins 3rd generation	Cefixime	CFM	5
	Monobactam	Aztreonam	ATM	30
	Beta lactamase inhibitor	Ampicillin/sulbactam	SAM	10/10
		Amoxicillin/clavulanic acid	AMC	20/10
Highly important	Tetracyclines	Tetracycline	TE	30
		Doxycycline	DO	30
	Phenicol	Chloramphenicol	CHL	30
	Folate pathway antagonists	Sulfamethoxazole/trimethoprim	SXT	23.75/1.25
	Cephalosporins, 1st generation	Cefadroxil	CFR	30
	Cephalosporins, 2nd generation	Cefaclor	CEC	30
Important	Nitroimidazoles	Metronidazole	MET	5
	Nitrofurans	Furazolidone	FR	50

**Table 2:** Primers, primer sequences, target genes, annealing temperature, and amplicon size of the used genes.

Antimicrobial Class	Target Gene	Sequence (5′ to 3′)	Size (bp)	Annealing Temperature	References
Beta-lactams	*bla*_CTX-M_ (ESBL)	F: ATGTGCAGYACCAGTAARGTKATGG R: TGGGTRAARTARGTSACCAGAAYCAGCGG	593	duplex PCR by 57 °C	[47]
	*bla*_TEM_	F: ATCAGCAATAAACCAGC R: CCCCGAAGAACGTTTTC	516		[48]
	*bla*_OXA-10_	F: TATCGCGTGTCTTTCGAGTA R: TTAGCCACCAATGATGCCC	760	57 °C	[49]
	*bla*_SHV_	F: AGGATTGACTGCCTTTTTG R: ATTTGCTGATTTCGCTCG	392	57 °C	[48]
ampC (class C)	*bla*_CMY-2_	F: AGCGATCCGGTCACGAAATA R: CCCGTTTTATGCACCCATGA	695	61 °C	[50]
Streptomycin	*aad*A1	F: TATCCAGCTAAGCGCGAACT R: ATTTGCCGACTACCTTGGTC	447	55 °C	[51]
Phenicols	*cat*A1	F: AGTTGCTCAATGTACCTATAACC R: TTGTAATTCATTAAGCATTCTGCG	547	55 °C	[52]
Quinolones	*qnr*A	F: ATTTCTCACGCCAGGATTTG R: GATCGGCAAAGGTTAGGTCA	516	Multiplex PCR by 55 °C	[53]
	*qnr*S	F: AGTGATCTCACCTTCACCGC R: CAGGCTGCAATTTTGATACC	552		[54]
Fluoroquinolones	*gyr*A	F: AGTGTAATTGTTGCCCG R: ATATCGCCATCAACCGA	470		[55]
Sulfonamides	*sul*2	F: CGGCATCGTCAACATAAACC R: GTGTGCGGATGAAGTCAG	722		[56]
Trimethoprim	*dfr*A1	F: GGAGTGCCAAAGGTGAACAGC R: GAGGCGAAGTCTTGGGTAAAAAC	367		[57]
Tetracyclines	*tet*(M)	F: GTGGACAAAGGTACAACGAG R: CGGTAAAGTTCGTCACACAC	406	55 °C	[58]
Macrolide	*erm*B	F: GAAAAGGTACTCAACCAAATA R: AGTAACGGTACTTAAATTTGTTTTAC	636	57 °C	[59]
	*msr*A	F: GCAAATGGTGTAGGTAAGACAACT R: ATCATGTGATGTAAACAAAAT	401	55 °C	[60]
Integron integrase gene class 1	*int*1	F: GCCTTGCTGTTCTTCTACGG R: GATGCCTGCTTGTTCTACGG	565	57 °C	[61]
Fosfomycin	*fos*A	F: ATCTGTGGGTCTGCCTGTCGT R: ATGCCCGCATAGGGCTTCT	271	55.6 °C	[62]
Polymyxins (colistin)	*mcr-*1	F: CGGTCAGTCCGTTTGTTC R: CTTGGTCGGTCTGTAGGG	309	57 °C	[63]
Beta-lactam (Carbapenemase)	*bla*_OXA-48_ (class D)	F: GCTTGACCCTCGATT R: GATTTGCTCCGTGGCCGAAA	281	60 °C	[64]
	*bla*_IMP_ (MBLs, class B)	F: TTGACACTCCATTTACDG R: GATYGAGAATTAAGCCACYCT	139	55 °C	
	*bla*_KPC_ (class A)	F: CATTCAAGGGCTTTCTTGCTGC R: ACGACGGCATAGTCATTTGC	538	55 °C	
	*bla*_GES_ (class A)	F: AGTCGGCTAGACCGGAAAG R: TTTGTCCGTGCTCAGGAT	399	57 °C	[65]
	*bla*_VIM_ (MBLs, class B)	F: GATGGTGTTTGGTCGCATA R: CGAATGCGCAGCACCAG	390	60 °C	[66]
	*bla*_NDM-1_ (MBLs, class B)	F: GGCGGAATGGCTCATCACGA R: CGCAACACAGCCTGACTTTC	287	55 °C	[67]

**Table 3:** Distribution of *Proteus* spp. and *P. mirabilis* isolates among various samples.

Sample Type	No. of *Proteus* spp. Isolates (%)	No. of *Proteus mirabilis* Isolates (%)
Broiler chickens		
	Total no. 50/450 (11.11%)	Total no. 34/50 (68)
Meat	10/50 (20)	8/10 (80)
Liver	5/50 (10)	3/5 (60)
Lung	1/50 (2)	0 (0)
Gall bladder	8/50 (16)	5/7 (71.43)
Kidney	2/50 (4)	0 (0)
Gizzard	8/50 (16)	4/8 (50)
Intestine	7/50 (14)	4/7 (57.14)
Spleen	7/50 (14)	6/7 (85.71)
Cloacal swabs	6/50 (12)	4/6 (66.67)
Buffaloes		
	Total no. 27/270 (10)	Total no. 11/27 (40.74)
Muscle	5/27 (18.52)	2/5 (40)
Liver	3/27 (11.11)	1/3 (33.33)
Gall bladder	1/27 (3.7)	0 (0)
Reticulum	3/27 (11.11)	0(0)
Rumen	4/27 (14.81)	3/4 (75)
Omasum	2/27 (7.41)	0(0)
Abomasum	4/27 (14.81)	3/4 (75)
Small intestine	2/27 (7.41)	0 (75)
Fecal matter	4/27 (14.81)	2/4 (50)

**Table 4:** Summary of MAR indices among XDR and PDR *P. mirabilis* isolates derived from broiler chickens and buffaloes.

Source	Resistance Type	No. of Resistant Isolates	% of Isolates	Antibiotic Classes Resistant	MAR Index Range
Broiler Chicken	XDR	17	50	11–12	0.846–0.923
Broiler Chicken	PDR	17	50	13	1
Buffalo	XDR	1	9.09	11	0.846
Buffalo	PDR	10	90.91	13	1

**Table 5:** Concordance between antimicrobial resistance genotypes and phenotypes in broiler chicken and buffalo isolates.

Antimicrobial Resistance Gene	Antibiotic Disk	Resistant Broiler Isolates No. 34	Resistant Buffalo Isolates No. 11	Gene-Resistant Broiler Chickens’ Isolates	Gene-Resistant Buffalo Isolates	Broiler Concordance %	Buffalo Chickens’ Concordance %
*bla*_TEM_	CFR CEC SAM AX CFM AMC ATM	34 (100%) 32 (94.12%) 34 (100%) 34 (100%) 29 (85.29%) 34 (100%) 30 (88.24%) (85.29–100%)	11 (100%) 11 (100%) 11 (100%) 11 (100%) 11 (100%) 11 (100%) 11 (100%) (100%)	(33/34) 97.06%	(9/11) 81.82%	97.06	81.82
*bla*_CTX-M_				(9/34) 26.47%	(2/11) 18.18%	26.47	18.18
*bla*_OXA-10_				(1/34) 2.94%	(2/11) 18.18%	2.94	18.18
*bla*_CMY-2_				(14/34) 41.18%	(7/11) 63.64%	41.18	63.64
*bla*_SHV_				0	0	0	0
*bla*_KPC_, *bla*_GES_, *bla*_VIM_, *bla*_IMP_, *bla*_NDM-1_, *bla*_OXA-48_	MEM	27 (79.41%)	11 (100%)	0	0	0	0
*sul*2	STX	34 (100%)	11 (100%)	(33/34) 97.06	(10/11) 90.91	97.06	90.91
*dfr*A1	STX	34 (100%)	11 (100%)	(34/34) 100	(11/11) 100	100	100
*qnr*A	CIP LEV	31 (91.18%) 31 (91.18%)	10 (90.91%) 10 (90.91%)	(16/34) 47.06	(1/11)9.09%	47.06	9.09
*qnr*S				0	0	0	0
*gyr*A				0	0	0	0
*erm*B	CL E	34 (100%) 34 (100%)	11 (100%) 11 (100%)	(8/34) 23.53	(2/11) 18.18	23.53	18.18
*msrA*				0	0	0	0
*tet*(M)	TE DO	34 (100%) 34 (100%)	11 (100%) 11 (100%)	(24/34) 70.79	(9/11) 81.82	70.79	81.82
*aad*A1	HLS	34 (100%)	11 (100%)	(33/34) 97.06	(11/11) 100	97.06	100
*cat*A1	CHL	34 (100%)	11 (100%)	(15/34) 44.12	(7/11) 63.64	44.12	63.64

**Table 6:** Enhanced antibacterial effect of CIP in combination with CS and its nanoparticles against resistant *P. mirabilis*.

Inhibition Zone	Broiler (N = 34)						Buffalo (N = 11)						*p*-Value ^2^
	Median	IQR	Zone Area = (πr^2^) (mm^2^)	Fold-Change (Area)	%	*p*-Value ^1^	Median	IQR	Zone Area = (πr^2^) (mm^2^)	Fold-Change (Area)	%	*p*-Value ^1^	
CIP (control)	1.52	0.725	3.14	1	-		1.6	0.233	3.36	1	-		0.6
CS 1%	1.68	0.5	1.81	-	-	<0.001 *	1.73	0.167	2	-	-	0.017 *	0.5
CS 2%	1.88	0.383	2.21	-	-	<0.001 *	1.9	0.183	2.34	-	-	<0.001 *	0.4
CSNPs	2	0.3	2.77	-	-	<0.001 *	2.07	0.1	2.80	-	-	<0.001 *	0.3
CS 1% + CIP	2.1	0.467	3.46	1.1	10	<0.001 *	2.1	0.2	3.46	1.02	2	<0.001 *	0.7
CS 2% + CIP	2.37	0.567	4.40	1.4	40	<0.001 *	2.4	0.4	4.50	1.4	40	<0.001 *	0.9
CSNPs + CIP	2.45	0.658	4.71	1.5	50	<0.001 *	2.6	0.383	5.30	1.58	58	<0.001 *	0.9

The antibacterial activity was determined using the disk diffusion method and expressed as the median inhibition zone diameter (mm) and the calculated zone area (mm^2^). The enhancement effect of chitosans with CIP is presented as the fold change in zone area and the percentage increase relative to the CIP control. *p*-value ^1^ indicates the statistical significance of the difference between each treatment and the CIP control group within each animal source, as calculated by the pairwise comparisons (Durbin–Conover) test. *p*-value ^2^ represents the statistical significance of the difference between the two animal sources (broiler vs. buffalo) for each treatment, as determined by the Mann–Whitney U test. An asterisk (*) denotes a statistically significant difference (*p* < 0.05). Abbreviations: CS, chitosan; CSNPs, chitosan nanoparticles; CIP, ciprofloxacin; IQR, interquartile range.

**Table 7:** Pairwise comparisons of antibacterial activity between different treatment regimens against resistant *P. mirabilis*.

Pairwise Comparisons of Inhibition Zone Measurements		Broiler (N = 34)		Buffalo (N = 11)	
		Median Difference	*p*-Value	Median Difference	*p*-Value
CSNPs + CIP	CS 2% + CIP	+0.08	<0.001 *	+0.2	<0.001 *
CSNPs + CIP	CS 1% + CIP	+0.35	<0.001 *	+0.5	<0.001 *
CSNPs + CIP	CSNPs	+0.57	<0.001 *	+0.7	<0.001 *
CSNPs + CIP	CS 2%	+0.77	<0.001 *	+0.87	<0.001 *
CSNPs + CIP	CS 1%	+0.93	<0.001 *	+1	<0.001 *
CSNPs + CIP	CIP	+0.45	<0.001 *	+0.53	<0.001 *
CS 2% + CIP	CS 1% + CIP	+0.27	<0.001 *	+0.3	<0.001 *
CS 2% + CIP	CSNPs	+0.49	<0.001 *	+0.33	<0.001 *
CS 2% + CIP	CS 2%	+0.69	<0.001 *	+0.67	<0.001 *
CS 2% + CIP	CS 1%	+0.85	<0.001 *	+0.8	<0.001 *
CS 2% + CIP	CIP	+0.37	<0.001 *	+0.33	<0.001 *
CS 1% + CIP	CSNPs	+0.22	<0.001 *	+0.2	0.094
CS 1% + CIP	CS 2%	+0.42	<0.001 *	+0.37	<0.001 *
CS 1% + CIP	CS 1%	+0.58	<0.001 *	+0.5	<0.001 *
CS 1% + CIP	CIP	+0.10	<0.001 *	+0.03	<0.001 *
CSNPs	CS 2%	+0.20	<0.001 *	+0.17	<0.001 *
CSNPs	CS 1%	+0.36	<0.001 *	+0.30	<0.001 *
CSNPs	CIP	−0.12	<0.001 *	−0.17	<0.001 *
CS 2%	CS 1%	+0.16	<0.001 *	0.13	<0.001 *
CS 2%	CIP	−0.32	<0.001 *	−0.34	<0.001 *
CS 1%	CIP	−0.48	<0.001 *	−0.47	0.017 *

*p*-values were calculated using the Durbin–Conover test for post hoc pairwise comparisons. An asterisk (*) denotes a statistically significant difference at *p* < 0.05.

**Table 8:** MIC and MBC values of CS formulations against resistant *P. mirabilis* isolates.

Results	Broiler (N = 34)		Buffalo (N = 11)		*p*-Value
	Median	IQR	Median	IQR	
CSNPs MIC	0.0672	0.0312	0.0813	0.025	0.283
CSNPs MBC	0.177	0.0833	0.167	0.0625	0.61
MBC/MIC Ratio	2.63	-	2.05	-	-
CS 2% MIC	4.19	1.64	3.25	0.906	0.086
CS 2% MBC	6.67	2.5	7.33	3.13	0.74
MBC/MIC Ratio	1.59	-	2.26	-	-
CS 1% MIC	4.5	2.12	3.56	1.44	0.328
CS 1% MBC	9.08	4.54	8.33	2.83	0.59
MBC/MIC Ratio	2.2	-	2.34	-	-

The *p*-value represents the statistical significance of differences between broiler and buffalo isolates for each formulation, as determined by the Mann–Whitney U test.

**Table 9:** Analysis of MIC and MBC of CS and its nanoparticles against *P. mirabilis* isolates.

Source	Agents (mg/mL)	Minimum	Q1	MIC50	Q3	MIC90	IQR	Maximum
MIC of buffalo	CS 1%	1.88	2.75	3.56	4.75	9	2	10
	CS 2%	2.25	2.25	3.25	3.63	4.5	1.38	5
	CSNPs	0.06	0.06	0.08	0.09	0.11	0.03	0.13
MIC of broiler chicken	CS 1%	1.75	3.25	4.5	5.38	6.5	2.13	7
	CS 2%	0.69	3.06	4.13	4.75	6.06	1.69	6.5
	CSNPs	0.04	0.06	0.07	0.09	0.11	0.03	0.2
		Minimum	Q1	MBC50	Q3	MBC90	IQR	Maximum
MBC of buffalo	CS 1%	5	5.83	8.33	9.83	9.83	4	9.83
	CS 2%	3.33	4.17	7.33	7.50	9	3.33	9
	CSNPs	0.08	0.13	0.17	0.21	0.21	0.08	0.21
MBC of broiler chicken	CS 1%	2.5	5	9	10	10	5	10
	CS 2%	2.92	5.83	6.67	8.33	8.33	2.5	8.33
	CSNPs	0.08	0.13	0.17	0.21	0.21	0.08	0.25

**Table 10:** Pairwise comparisons of MIC and MBC values among CS formulations in broiler and buffalo isolates of resistant *P. mirabilis*.

Pairwise Comparisons	Median Difference	*p*-Value
MIC comparisons in broiler		
CSNPs vs. CS 2%	−4.1203	<0.001 *
CSNPs vs. CS 1%	−4.4328	<0.001 *
CS 2% vs. CS 1%	−0.3125	0.468
MIC Comparisons in buffalo		
CSNPs vs. CS 2%	−3.1687	<0.001 *
CSNPs vs. CS 1%	−3.4812	<0.001 *
CS 2% vs. CS 1%	−0.3125	0.001 *
MBC Comparisons in broiler		
CSNPs vs. CS 2%	−6.5	<0.001 *
CSNPs vs. CS 1%	−8.9	<0.001 *
CS 2% vs. CS 1%	−2.4	0.003 *
MBC Comparisons in buffalo		
CSNPs vs. CS 2%	−7.16	<0.001 *
CSNPs vs. CS 1%	−8.16	<0.001 *
CS 2% vs. CS 1%	−1	0.001 *

Pairwise comparisons of MIC and MBC values (mg/mL) among different CS formulations within broiler and buffalo isolates. Values represent median differences calculated as (first formulation–second formulation). A negative value indicates that the first formulation has a lower MIC or MBC value than the second formulation. *p*-values were derived from Durbin–Conover’s post hoc tests. * Statistically significant at *p* < 0.05.

**Table 11:** Inhibitory effect of sub-MIC of CS and CSNPs on swarming motility of broiler chickens and buffalo-originated *P. mirabilis*.

Swarming Motility Results	Broiler (N = 34)		Buffalo (N = 11)		*p*-Value
	Median	IQR	Median	IQR	
Control	4.27	1.64	4.27	1.4	0.46
CS 2%	2.33	0.69	2.2	0.38	0.74
CSNPs	2.20	0.55	2.07	0.35	0.67

The *p*-value represents the statistical significance of differences between broiler and buffalo isolates for each treatment, as determined by the Mann–Whitney U test.

**Table 12:** Pairwise comparisons of swarming motility inhibition by sub-MIC chitosan formulations in *P. mirabilis* isolates.

Pairwise Comparisons		Median Difference	Reduction %	*p*-Value
Pairwise comparisons of swarming motility in broiler				
Control	CS 2٪	+1.94	45.4	<0.001 *
Control	CSNPs	+2.07	48.4	<0.001 *
CSNPs	CS 2٪	+0.13		<0.001 *
Pairwise comparisons of swarming motility in buffalo				
Control	CS 2٪	+2.07	48.4	<0.001 *
Control	CSNPs	+2.2	51.5	<0.001 *
CSNPs	CS 2٪	+0.13		<0.001 *

Reduction percentage was calculated as: [(Control − Treatment)/Control] × 100%. Pairwise comparisons were analyzed using the Durbin–Conover test, with asterisk (*) indicating statistical significance at *p* < 0.05.

## Data Availability

The current study includes all information that was gathered throughout this manuscript. The data supporting the study conclusions are available from the authors upon request.

## Supplementary Materials

The following supporting information can be downloaded at: https://www.mdpi.com/article/10.3390/pathogens14111176/s1, Figure S1: Agarose gel electrophoresis profile for the detection of *Proteus* spp. Specific 16S rRNA gene (857 bp); Figure S2: Agarose gel electrophoresis profile for the detection of *P. mirabilis* specific 16S rRNA gene (1496 bp); Figure S3: Agarose gel electrophoresis profile for the duplex PCR detection of resistance genes *bla*_CTX-M_ (593 bp) and *bla*_TEM_ (516 bp); Figure S4: Agarose gel electrophoresis profile for the detection of resistance gene *bla*_OXA-10_ (760 bp); Figure S5: Agarose gel electrophoresis profile for the detection of resistance gene *bla*_CMY-2_ (695 bp); Figure S6: Agarose gel electrophoresis profile for the detection of resistance gene *aad*A1 (447 bp); Figure S7: Agarose gel electrophoresis profile for the detection of resistance gene *cat*A1 (547 bp); Figure S8: Agarose gel electrophoresis profile for the multiplex PCR detection of resistance genes *dfr*A1 (367 bp), *qnr*A (516 bp), and *sul*2 (722 bp); Figure S9: Agarose gel electrophoresis profile for the detection of resistance gene *tet*(M) (406 bp); Figure S10: Agarose gel electrophoresis profile for the detection of resistance gene *erm*B (636 bp); Figure S11: Agarose gel electrophoresis profile for the detection of resistance gene *int*1 (565 bp); Figure S12: Agarose gel electrophoresis profile for the detection of resistance gene *mcr-*1 (309 bp); Figure S13: Size distribution by volume of the synthesized CSNPs as determined by DLS; Table S1: The inhibitory zone diameters (in cm) of CS (1% and 2%), CSNPs, CIP, and their combinations against *P. mirabilis* isolates from broiler chickens and buffalo; Table S2: MIC (mg/mL) values of CS (1% and 2%) and CSNPs against P. mirabilis isolates; Table S3: MIC distribution analysis for *P. mirabilis* isolates from broiler chickens; Table S4: MIC distribution analysis for *P. mirabilis* isolates from buffalo; Table S5: Cumulative MIC distribution analysis for *P. mirabilis* isolates from both broiler chickens and buffalo; Table S6: MBC (mg/mL) values of CS (1% and 2%) and CSNPs against *P. mirabilis* isolates; Table S7: MBC distribution analysis for *P. mirabilis* isolates from buffalo; Table S8: MBC distribution analysis for *P. mirabilis* isolates from broiler chickens; Table S9: Cumulative MBC distribution analysis for *P. mirabilis* isolates from both broiler chickens and buffalo; Table S10: Swarming motility diameter (in cm) of *P. mirabilis* isolates under control conditions, and after treatment with CS 2% and CSNPs.

## References

[1] Najim H.T., Farhan A.A., Athab A.M. (2018). Bacteriological Study of the Bacteria Cause Urinary Tract Infection of Patients Admitted to Cardiac Care Unite a Baqubah General Teaching Hospital. Diyala J. Med..

[2] Saif Y.M. (2008). Diseases of Poultry. Other Bacterial Diseases.

[3] Sanches M.S., Baptista A.A.S., de Souza M., Menck-Costa M.F., Justino L., Nishio E.K., Rocha S.P.D. (2020). *Proteus mirabilis* causing cellulitis in broiler chickens. Braz. J. Microbiol..

[4] Schaffer J.N., Pearson M.M. (2017). Proteus mirabilis and urinary tract infections. Urinary Tract Infections: Molecular Pathogenesis and Clinical Management.

[5] Sanches M.S., Baptista A.A.S., de Souza M., Menck-Costa M.F., Koga V.L., Kobayashi R.K.T., Rocha S.P.D. (2019). Genotypic and phenotypic profiles of virulence factors and antimicrobial resistance of *Proteus mirabilis* isolated from chicken carcasses: Potential zoonotic risk. Braz. J. Microbiol..

[6] Chen L., Zhang Y., Du J., Zhang X., Li M., Chen H. (2018). Description and plasmid characterization of the qnrD determinant in Proteeae in Wenzhou, Southern China. J. Microbiol. Immunol. Infect..

[7] Aygül A., Öztürk İ., Çilli F.F., Ermertcan Ş. (2019). Quercetin inhibits swarming motility and activates biofilm production of *Proteus mirabilis* possibly by interacting with central regulators, metabolic status or active pump proteins. Phytomedicine.

[8] Bloch J., Lemaire X., Legout L., Ferrby D., Yazdanpanah Y., Senneville E. (2011). Brain abscesses during *Proteus vulgaris* bacteremia. Neurol. Sci..

[9] Chakkour M., Hammoud Z., Farhat S., El Roz A., Ezzeddine Z., Ghssein G. (2024). Overview of *Proteus mirabilis* pathogenicity and virulence. Insights into the role of metals. Front. Microbiol..

[10] Zhang J., Hoedt E.C., Liu Q., Berendsen E., The J.J., Hamilton A. (2021). Elucidation of *Proteus mirabilis* as a key bacterium in Crohn’s disease inflammation. Gastroenterology.

[11] Chen S.L., Kang Y.T., Liang Y.H., Qiu X.T., Li Z.J. (2023). A core genome multilocus sequence typing scheme for *Proteus mirabilis*. Biomed. Environ. Sci..

[12] Kearns D.B., Losick R. (2003). Swarming motility in undomesticated *Bacillus subtilis*. Mol. Microbiol..

[13] Rather P.N. (2005). Swarmer cell differentiation in *Proteus mirabilis*. Environ. Microbiol..

[14] Magiorakos A.P., Srinivasan A., Carey R.B., Carmeli Y., Falagas M.E., Giske C.G., Harbarth S., Hindler J.F., Kahlmeter G., Olsson-Liljequist B. (2012). Multidrug-resistant, extensively drug-resistant and pandrug-resistant bacteria: An international expert proposal for interim standard definitions for acquired resistance. Clin. Microbiol. Infect..

[15] OECD (2018). Stemming the Superbug Tide Just a Few Dollars More.

[16] Al-Saadi N., Alsallami D., Alsultan A., Al-hriahaw H. (2022). Whole-genomic sequence of multidrug resistance burkholderia cepacia associated with acute suppurative thyroiditis. J. Complement. Med. Res..

[17] Girlich D., Bonnin R.A., Dortet L., Naas T. (2020). Genetics of acquired antibiotic resistance genes in *Proteus* spp. Front. Microbiol..

[18] Pitout J.D., Nordmann P., Laupland K.B., Poirel L. (2005). Emergence of Enterobacteriaceae producing extended-spectrum β-lactamases (ESBLs) in the community. J. Antimicrob. Chemother..

[19] Lim E.J., Ho S.X., Cao D.Y., Lau Q.C., Koh T.H., Hsu L.Y. (2016). Extended-Spectrum Beta-Lactamase-Producingin Retail Chicken Meat in Singapore. Ann. Acad. Med. Singap..

[20] Bush K., Jacoby G.A. (2010). Updated functional classification of β-lactamases. Antimicrob. Agents Chemother..

[21] Shaaban M., Elshaer S.L., Abd El-Rahman O.A. (2022). Prevalence of extended-spectrum β-lactamases, AmpC, and carbapenemases in *Proteus mirabilis* clinical isolates. BMC Microbiol..

[22] Rao M.J., Harle S., Padmavathy M. (2018). Prevalence of extended spectrum beta-lactamases and amp-c beta-lactamases in clinical isolates of gram-negative bacilli at a tertiary care hospital. J. Evol. Med. Dent. Sci..

[23] Bratu S., Landman D., Haag R., Recco R., Eramo A., Alam M., Quale J. (2005). Rapid spread of carbapenem-resista Bush nt *Klebsiella pneumoniae* in New York City: A new threat to our antibiotic armamentarium. Arch. Intern. Med..

[24] Brink A.J., Coetzee J., Clay C.G., Sithole S., Richards G.A., Poirel L., Nordmann P. (2012). Emergence of New Delhi metallo-beta-lactamase (NDM-1) and *Klebsiella pneumoniae* carbapenemase (KPC-2) in South Africa. J. Clin. Microbiol..

[25] Queenan A.M., Bush K. (2007). Carbapenemases: The versatile β-lactamases. Clin. Microbiol. Rev..

[26] Nordmann P., Naas T., Poirel L. (2011). Global spread of carbapenemase-producing Enterobacteriaceae. Emerg. Infect. Dis..

[27] Hughes D., Andersson D.I. (2017). Environmental and genetic modulation of the phenotypic expression of antibiotic resistance. FEMS Microbiol. Rev..

[28] Hu R., Wang X., Muhamamd I., Wang Y., Dong W., Zhang H. (2020). Biological characteristics and genetic analysis of a highly pathogenic *Proteus mirabilis* strain isolated from dogs in China. Front. Vet. Sci..

[29] Leverstein-van Hall M.A., MBlok H.E., TDonders A.R., Paauw A., Fluit A.C., Verhoef J. (2003). Multidrug resistance among Enterobacteriaceae is strongly associated with the presence of integrons and is independent of species or isolate origin. J. Infect. Dis..

[30] Liu J.H., Liu Y.Y., Shen Y.B., Yang J., Walsh T.R., Wang Y., Shen J. (2024). Plasmid-mediated colistin-resistance genes: Mcr. Trends Microbiol..

[31] Wang R., Van Dorp L., Shaw L.P., Bradley P., Wang Q., Wang X. (2018). The global distribution and spread of the mobilized colistin resistance gene mcr-1. Nat. Commun..

[32] Al-Kadmy I.M., Ibrahim S.A., Al-Saryi N., Aziz S.N., Besinis A., Hetta H.F. (2020). Prevalence of genes involved in colistin resistance in *Acinetobacter baumannii*: First report from Iraq. Microb. Drug Resist..

[33] Said H.S., Abdelmegeed E.S. (2019). Emergence of multidrug resistance and extensive drug resistance among enterococcal clinical isolates in Egypt. Infect. Drug Resist..

[34] Cai J., Yang T., Chen H., Bai Y. (2021). Advances in the application of bacteriophage in the inhibiting of bacteria. Heilongjiang Anim. Sci. Vet. Med..

[35] Dedloff M.R., Effler C.S., Holban A.M., Gestal M.C. (2019). Use of biopolymers in mucosally-administered vaccinations for respiratory disease. Materials.

[36] Shoueir K.R., El-Desouky N., Rashad M.M., Ahmed M.K., Janowska I., El-Kemary M. (2021). Chitosan based-nanoparticles and nanocapsules: Overview, physicochemical features, applications of a nanofibrous scaffold, and bioprinting. Int. J. Biol. Macromol..

[37] Li J., Zhuang S. (2020). Antibacterial activity of chitosan and its derivatives and their interaction mechanism with bacteria: Current state and perspectives. Eur. Polym. J..

[38] Dutta P.K., Tripathi S., Mehrotra G.K., Dutta J. (2009). Perspectives for chitosan based antimicrobial films in food applications. Food Chem..

[39] Ishaq K., Ahmad A., Rafique A., Aslam R., Ali S., Shahid M.A., Sarwar N., Aslam M.A., Aslam B., Arshad M.I. (2022). Occurrence and antimicrobial susceptibility of *Proteus mirabilis* from chicken carcass. Pak. Vet. J..

[40] Markey B., Leonard F., Archambault M., Cullinane A., Maguire D. (2013). Clinical Veterinary Microbiology.

[41] Zahraei S.M., Rabani K.M., Safarchi A., Peyghambari S.M., Mahzounieh M. (2007). Detection of stx1, stx2, eae, espB and hly genes in avian pathogenic *Escherichia coli* by multiplex polymerase chain reaction. J. Vet. Res..

[42] Kadhim AL-Imam M.J., AL-Rubaii B.A.L. (2016). The influence of some amino acids, vitamins and anti-inflammatory drugs on activity of chondroitinase produced by *Proteus vulgaris* caused urinary tract infection. Iraqi J. Sci..

[43] Al-Mudallal N.H., Khudair A.M., Abdoul Hadi Alsakini A.H., Zidane N.A. (2021). Molecular detection and phylogenetic analysis of 16s RNA gene of *Proteus mirabilis* isolated from different clinical sources in Baghdad hospitals. Biochem. Cell Arch..

[44] Malvern P.A., CLSI (2024). Performance standards for antimicrobial disk susceptibility testing. CLSI Supplement M100.

[45] Decôme M., Cuq B., Fairbrother J.H., Gatel L., Conversy B. (2020). Clinical significance of *Proteus mirabilis* bacteriuria in dogs, risk factors and antimicrobial susceptibility. Can. J. Vet. Res..

[46] World Health Organization (2017). Critically Important Antimicrobials for Human Medicine: Ranking of Antimicrobial Agents for Risk Management of Antimicrobial Resistance Due to Non-Human Use.

[47] Archambault M., Petrov P., Hendriksen R.S., Asseva G., Bangtrakulnonth A., Hasman H., Aarestrup F.M. (2006). Molecular characterization and occurrence of extended-spectrum β-lactamase resistance genes among *Salmonella enterica serovar* Corvallis from Thailand, Bulgaria, and Denmark. Microb. Drug Resist..

[48] Colom K., Pérez J., Alonso R., Fernández-Aranguiz A., Lariño E., Cisterna R. (2003). Simple and reliable multiplex PCR assay for detection of bla TEM, bla SHV and bla OXA–1 genes in Enterobacteriaceae. FEMS Microbiol. Lett..

[49] Mirsalehian A., Feizabadi M., Nakhjavani F.A., Jabalameli F., Goli H., Kalantari N. (2010). Detection of VEB-1, OXA-10 and PER-1 genotypes in extended-spectrum β-lactamase-producing Pseudomonas aeruginosa strains isolated from burn patients. Burns.

[50] Kim J., Jeon S., Rhie H., Lee B., Park M., Lee H. (2009). Rapid detection of extended spectrum β-lactamase (ESBL) for Enterobacteriaceae by use of a multiplex PCR-based method. Infect. Chemother..

[51] Randall L.P., Cooles S.W., Osborn M.K., Piddock L.J.V., Woodward M.J. (2004). Antibiotic resistance genes, integrons and multiple antibiotic resistance in thirty-five serotypes of *Salmonella enterica* isolated from humans and animals in the UK. J. Antimicrob. Chemother..

[52] Van T.T.H., Chin J., Chapman T., Tran L.T., Coloe P.J. (2008). Safety of raw meat and shellfish in Vietnam: An analysis of *Escherichia coli* isolations for antibiotic resistance and virulence genes. Int. J. Food Microbiol..

[53] Robicsek A., Strahilevitz J., Sahm D.F., Jacoby G.A., Hooper D.C. (2006). qnr prevalence in ceftazidime-resistant *Enterobacteriaceae* isolates from the United States. Antimicrob. Agents Chemother..

[54] López M., Tenorio C., Del Campo R., Zarazaga M., Torres C. (2011). Characterization of the mechanisms of fluoroquinolone resistance in vancomycin-resistant enterococci of different origins. J. Chemother..

[55] Godreuil S., Galimand M., Gerbaud G., Jacquet C., Courvalin P. (2003). Efflux pump Lde is associated with fluoroquinolone resistance in *Listeria monocytogenes*. Antimicrob. Agents Chemother..

[56] Poppe C., Martin L., Muckle A., Archambault M., McEwen S., Weir E. (2006). Characterization of antimicrobial resistance of *Salmonella* Newport isolated from animals, the environment, and animal food products in Canada. Can. J. Vet. Res..

[57] Toro C.S., Farfán M., Contreras I., Flores O., Navarro N., Mora G.C., Prado V. (2005). Genetic analysis of antibiotic-resistance determinants in multidrug-resistant Shigella strains isolated from Chilean children. Epidemiol. Infect..

[58] Warsa U.C., Nonoyama M., Ida T., Okamoto R., Okubo T., Shimauchi C., Kuga A., Inoue M. (1996). Detection of tet(K) and tet(M) in *Staphylococcus aureus* of Asian countries by the polymerase chain reaction. J. Antibiot..

[59] Flórez A.B., Alegría Á., Rossi F., Delgado S., Felis G.E., Torriani S., Mayo B. (2014). Molecular identification and quantification of tetracycline and erythromycin resistance genes in Spanish and Italian retail cheeses. Biomed. Res. Int..

[60] Morvan A., Moubareck C., Leclercq A., Hervé-Bazin M., Bremont S., Lecuit M., Courvalin P., Monnier A. (2010). Antimicrobial resistance of *Listeria monocytogenes* strains isolated from humans in France. Antimicrob. Agents Chemother..

[61] Levesque C., Piche L., Larose C., Roy P.H. (1995). PCR mapping of integrons reveals several novel combinations of resistance genes. Antimicrob. Agents Chemother..

[62] Liu P., Chen S., Wu Z.Y., Qi M., Li X.Y., Liu C.X. (2020). Mechanisms of fosfomycin resistance in clinical isolates of carbapenem-resistant *Klebsiella pneumoniae*. J. Glob. Antimicrob. Resist..

[63] Liu Y.-Y., Wang Y., Walsh T.R., Yi L.-X., Zhang R., Spencer J., Doi Y., Tian G., Dong B., Huang X. (2016). Emergence of plasmid-mediated colistin resistance mechanism MCR-1 in animals and human beings in China: A microbiological and molecular biological study. Lancet Infect. Dis..

[64] Dallenne C., Da Costa A., Decre D., Favier C., Arlet G. (2010). Development of a set of multiplex PCR assays for the detection of genes encoding important B-lactmases in Enterobacteriaceae. J. Antimicrobe Chemother..

[65] Hou M., Chen N., Dong L., Fang Y., Pan R., Wang W., Wang L., Ning J., Dong H. (2022). Molecular epidemiology, clinical characteristics and risk factors for bloodstream infection of multidrug-resistant *Klebsiella pneumoniae* infections in pediatric patients from Tianjin, China. Infect. Drug Resist..

[66] Sjölander I., Hansen F., Elmanama A., Khayyat R., Abu-Zant A., Hussein A., Taha A.A., Hammerum A.M., Ciofu O. (2014). Detection of NDM-2-producing *Acinetobacter baumannii* and VIM-producing Pseudomonas aeruginosa in Palestine. J. Glob. Antimicrobe Resist..

[67] Xia C., Yan R., Liu C., Zhai J., Zheng J., Chen W., Cao X. (2024). Epidemiological and genomic characteristics of global bla NDM-carrying *Escherichia coli*. Ann. Clin. Microbiol. Antimicrob..

[68] Muller R., Mader K., Gohla S. (2000). Solid lipid nanoparticles (SLN) for controlled drug delivery—A review of the state of the Art. Eur. J. Pharm. Biopharm..

[69] Fasihnia S.H., Peighambardoust S.H., Peighambardoust S.J., Oromiehie A., Soltanzadeh M., Peressini D. (2020). Migration analysis, antioxidant, and mechanical characterization of polypropylene-based active food packaging films loaded with BHA, BHT, and TBHQ. J. Food Sci..

[70] Ali N.G., Ali T.E.S., Kamel M.F., Saleh R., Sherif A.H., Aboyadak I.M. (2022). Eradication of Livoneca redmanii infestation in cultured *Argycrosomus regius*. Aquaculture.

[71] EUCASTT (2000). European Committee for Antimicrobial Susceptibility Testing of the European Society of Clinical Microbiology and Infectious Diseases (ESCMID). Determination of minimum inhibitory concentrations (MICs) of antibacterial agents by agar dilution. Clin. Microbiol. Infect..

[72] Elshikh M., Ahmed S., Funston S., Dunlop P., McGaw M., Marchant R., Banat I.M. (2016). Resazurin-based 96-well plate microdilution method for the determination of minimum inhibitory concentration of biosurfactants. Biotechnol. Lett..

[73] Diao Y., Yu X., Zhang C., Jing Y. (2020). Quercetin-grafted chitosan prepared by free radical grafting: Characterization and evaluation of antioxidant and antibacterial properties. J. Food Sci. Technol..

[74] Abedian Z., Jenabian N., Moghadamnia A.A., Zabihi E., Tashakorian H., Rajabnia M., Sadighian F., Bijani A. (2019). Antibacterial activity of high-molecular-weight and low-molecular-weight chitosan upon oral pathogens. J. Conserv. Dent..

[75] Qi L., Xu Z., Jiang X., Hu C., Zou X. (2004). Preparation and antibacterial activity of chitosan nanoparticles. Carbohydr. Res..

[76] French G.L. (2006). Bactericidal agents in the treatment of MRSA infections—The potential role of daptomycin. J. Antimicrob. Chemother..

[77] Jeong Y., Lim D.W., Choi J. (2014). Assessment of size-dependent antimicrobial and cytotoxic properties of silver nanoparticles. Adv. Mater. Sci. Eng..

[78] Nahar A., Siddiquee M., Nahar S., Anwar K.S., Islam S. (2014). Multidrug resistant-*Proteus mirabilis* isolated from chicken droppings in commercial poultry farms: Bio-security concern and emerging public health threat in Bangladesh. J. Biosaf. Health Educ..

[79] El-Demerdash A.S., Aggour M.G., El-Azzouny M.M., Abou-Khadra S.H. (2018). Molecular analysis of integron gene cassette arrays associated multi-drug resistant Enterobacteriaceae isolates from poultry. Cell Mol. Biol..

[80] Zhu X., Zhang Y., Shen Z., Xia L., Wang J., Zhao L., Wang K., Wang W., Hao Z., Liu Z. (2021). Characterization of NDM-1-producing carbapenemase in *Proteus mirabilis* among broilers in China. Microorganisms.

[81] Koirala B., Bhattarai R., Maharjan R., Maharjan S., Shrestha S. (2020). Bacterial Assessment of Buffalo Meat in Kathmandu Valley. Nepal J. Sci. Technol..

[82] Mansour S.N., Youssef W., Hana Y., Elias R.S., Nagib H.E., Hakim A.S., Younis E.M., Dapgh A.N. (2023). Multidrug Resistance in Gram Negative Bacteria Isolated from Cases of Mastitis in Buffaloes. Egypt. J. Anim. Health.

[83] Mirzaei A., Habibi M., Bouzari S., Asadi Karam M.R. (2019). Characterization of antibiotic-susceptibility patterns, virulence factor profiles and clonal relatedness in *Proteus mirabilis* isolates from patients with urinary tract infection in Iran. Infect. Drug Resist..

[84] Li Z., Peng C., Zhang G., Shen Y., Zhang Y., Liu C., Liu M., Wang F. (2022). Prevalence and characteristics of multidrug-resistant *Proteus mirabilis* from broiler farms in Shandong Province, China. Poult. Sci..

[85] Ramatla T., Ramaili T., Lekota K., Mileng K., Ndou R., Mphuthi M., Khasapane N., Syakalima M., Thekisoe O. (2024). Antibiotic resistance and virulence profiles of *Proteus mirabilis* isolated from broiler chickens at abattoir in South Africa. Vet. Med. Sci..

[86] El-Saeed B.A., Elshebrawy H.A., Zakaria A.I., Abdelkhalek A., Imre K., Morar A., Herman V., Sallam K.I. (2024). Multidrug-resistant *Proteus mirabilis* and other gram-negative species isolated from native Egyptian chicken carcasses. Trop. Med. Infect. Dis..

[87] Ma S., Shen J., Xu Y., Ding P., Gao X., Pan Y., Wu H., Hu G., He D. (2023). Epidemic characteristics of the SXT/R391 integrated conjugative elements in multidrug-resistant *Proteus mirabilis* isolated from chicken farm. Poult. Sci..

[88] Yu Z., Joossens M., Van den Abeele A.M., Kerkhof P.J., Houf K. (2021). Isolation, characterization and antibiotic resistance of *Proteus mirabilis* from Belgian broiler carcasses at retail and human stool. Food Microbiol..

[89] Sanches M.S., Silva L.C., Silva C.R.D., Montini V.H., Oliva B.H.D.d., Guidone G.H.M., Nogueira M.C.L., Menck-Costa M.F., Kobayashi R.K.T., Vespero E.C. (2023). Prevalence of Antimicrobial Resistance and Clonal Relationship in ESBL/AmpC-Producing *Proteus mirabilis* isolated from Meat products and Community-acquired urinary tract infection (UTI-CA) in Southern Brazil. Antibiotics.

[90] Sun Y., Wen S., Zhao L., Xia Q., Pan Y., Liu H., Wei C., Chen H., Ge J., Wang H. (2020). Association among biofilm formation, virulence gene expression, and antibiotic resistance in *Proteus mirabilis* isolates from diarrhetic animals in Northeast China. BMC Vet. Res..

[91] Ma W.Q., Han Y.Y., Zhou L., Peng W.Q., Mao L.Y., Yang X., Wang Q., Zhang T.J., Wang H.N., Lei C.W. (2022). Contamination of *Proteus mirabilis* harbouring various clinically important antimicrobial resistance genes in retail meat and aquatic products from food markets in China. Front. Microbiol..

[92] Ram V.P., Rao L.V., Rao T.S., Subramanyam K.V., Suresh Y., Srinivas K. (2022). A Study on Antibiogram and Beta-lactam Resistance of *Proteus mirabilis* Isolated from Animals and Humans in Andhra Pradesh, India. Indian J. Anim. Res..

[93] von Tippelskirch P., Gölz G., Projahn M., Daehre K., Friese A., Roesler U., Alter T., Orquera S. (2018). Prevalence and quantitative analysis of ESBL and AmpC beta-lactamase producing *Enterobacteriaceae* in broiler chicken during slaughter in Germany. Int. J. Food Microbiol..

[94] Hu H., Wu K., Zhang T., Mou Y., Liu L., Wang X., Xu W., Chen W., Chen X., Wang H. (2025). Whole Genome Analysis of *Proteus mirabilis* in a Poultry Breeder Farm Reveals the Dissemination of bla NDM and bla CTX-M Mediated by Diverse Mobile Genetic Elements. Agriculture.

[95] Gür D. (2004). General Characteristics of ESBLs and ESBL Types, New and Reemerging Infections (ESBL’lerin Genel Özellikleri ve ESBL Tipleri, Yeni ve Yeniden Gündeme Gelen Infeksiyonlar In Turkish).

[96] Almeida H.F.D., Trindade P.R.C.M., Teixeira C.R.V., Brito C.O., Dolabella S.S., Jain S., Martins M.P., Barbosa A.A.T. (2025). Antibiotic resistance gene occurrence in poultry farms in northeast Brazil. Ciênc. Anim. Bras..

[97] Sarwar A., Aslam B., Mahmood S., Muzammil S., Siddique A.B., Sarwar F., Khurshid M., Rasool M.H., Sasanya J., Aljasir S.F. (2025). Distribution of multidrug-resistant *Proteus mirabilis* in poultry, livestock, fish, and the related environment: One Health heed. Vet. World.

[98] Ejaz H., Younas S., Abosalif K.O., Junaid K., Alzahrani B., Alsrhani A., Abdalla A.E., Ullah M.I., Qamar M.U., Hamam S.S.M. (2021). Molecular analysis of *bla*_SHV_, *bla*_TEM_, and *bla*_CTX-M_ in extended-spectrum β-lactamase producing *Enterobacteriaceae* recovered from fecal specimens of animals. PLoS ONE.

[99] Ogefere H.O., Osikobia J.G., Omoregie R. (2016). Prevalence of AmpC β-lactamase among Gram-negative bacteria recovered from clinical specimens in Benin City, Nigeria. Trop. J. Pharm. Res..

[100] Girlich D., Bonnin R.A., Bogaerts P., De Laveleye M., Huang D.T., Dortet L., Glaser P., Glupczynski Y., Naas T. (2017). Chromosomal amplification of the blaOXA-58 carbapenemase gene in a *Proteus mirabilis* clinical isolate. Antimicrob. Agents Chemother..

[101] Mushi M.F., Mshana S.E., Imirzalioglu C., Bwanga F. (2014). Carbapenemase genes among multidrug resistant gram-negative clinical isolates from a tertiary hospital in Mwanza, Tanzania. Biomed. Res. Int..

[102] ElTaweel M., Said H.S., Barwa R. (2024). Emergence of extensive drug resistance and high prevalence of multidrug resistance among clinical *Proteus mirabilis* isolates in Egypt. Ann. Clin. Microbiol. Antimicrob..

[103] Moawad A.A., Hotzel H., Neubauer H., Ehricht R., Monecke S., Tomaso H., Hafez H.M., Roesler U., El-Adawy H. (2018). Antimicrobial resistance in *Enterobacteriaceae* from healthy broilers in Egypt: Emergence of colistin-resistant and extended-spectrum beta-lactamase-producing *Escherichia coli*. Gut Pathog..

[104] Saiprasad P.V., Krishnaprasad K. (2016). Exploring the hidden potential of fosfomycin for the fight against severe Gram-negative infections. Indian J. Med. Microbiol..

[105] World Health Organization (2012). WHO Advisory Group on Integrated Surveillance of Antimicrobial Resistance (AGISAR). Critically important antimicrobials for human medicine 3rd Revision 2011. WHO Document Production Services, Geneva, Switzerland. Clin. Infect. Dis..

[106] Lalezadeh A., Ghotaslou P., Ghotaslou R. (2023). The Detection of Fosfomycin-Modifying Enzymes (fos) in Uropathogenic Enterobacterale, Azerbaijan, Iran. Can. J. Infect. Dis. Med. Microbiol..

[107] Tewari R., Mitra S., Venugopal N., Das S., Ganaie F., Sen A., Shome R., Rahman H., Shome B.R. (2019). Phenotypic and molecular characterization of extended spectrum β-lactamase, ampc β-lactamase and metallo β-lactamase producing *Klebsiella* spp. from farm animals in India. Indian J. Anim. Res..

[108] Algammal A.M., Hashem H.R., Alfifi K.J., Hetta H.F., Sheraba N.S., Ramadan H., El-Tarabili R.M. (2021). *atp*D gene sequencing, multidrug resistance traits, virulence-determinants, and antimicrobial resistance genes of emerging XDR and MDR-*Proteus mirabilis*. Sci. Rep..

[109] Singh S., Yadav A.S., Singh S.M., Bharti P. (2010). Prevalence of Salmonella in chicken eggs collected from poultry farms and marketing channels and their antimicrobial resistance. Food Res. Int..

[110] Little K., Austerman J., Zheng J., Gibbs K.A. (2019). Cell shape and population migration are distinct steps of *Proteus mirabilis* swarming that are decoupled on high-percentage agar. J. Bacteriol..

[111] Vaezifar S., Razavi S., Golozar M.A., Karbasi S., Morshed M., Kamali M. (2013). Effects of some parameters on particle size distribution of chitosan nanoparticles prepared by ionic gelation method. J. Clust. Sci..

[112] Essa E.E., Hamza D., Khalil M.M., Zaher H., Salah D., Alnemari A.M., Rady M.H., Momen S.A.A. (2022). The antibacterial activity of Egyptian wasp chitosan-based nanoparticles against important antibiotic-resistant pathogens. Molecules.

[113] Loutfy S.A., El-Din H.M.A., Elberry M.H., Allam N.G., Hasanin M.T.M., Abdellah A.M. (2016). Synthesis, characterization and cytotoxic evaluation of chitosan nanoparticles: In vitro liver cancer model. Adv. Natl. Sci. Nanosci. Nanotechnol..

[114] Godoy C.A., Balic I., Moreno A.A., Diaz O., Colarte C.A., Larenas T.B., Gamboa A., Fuentes N.C. (2025). Antimicrobial and Antibiofilm Activity of Chitosan Nanoparticles Against *Staphylococcus aureus* Strains Isolated from Bovine Mastitis Milk. Pharmaceutics.

[115] Kulig D., Zimoch-Korzycka A., Jarmoluk A., Marycz K. (2016). Study on alginate–chitosan complex formed with different polymers ratio. Polymers.

[116] Kadhum W.N., Zaidan I.A. (2020). The synergistic effects of chitosan-alginate nanoparticles loaded with doxycycline antibiotic against multidrug resistant *Proteus mirabilis*, *Escherichia coli* and enterococcus faecalis. Iraqi J. Sci..

[117] Chandrasekaran M., Kim K.D., Chun S.C. (2020). Antibacterial activity of chitosan nanoparticles: A review. Processes.

[118] Hasan S.A. (2025). Evaluation of Trimethoprim Nanoemulsion for Combating Antibiotic-Resistant *Proteus mirabilis* in Urinary Tract Infections. Iran. J. Med. Microbiol..

[119] Nissanka N.M.C., Priyadarshana G., Dilhari K.A.A., Munasinghe J.A., Dilshani M., Weerasekera M.M. (2025). Curcumin-Modified Silver Nanoparticles’ Bioactivities Against Biofilm Forming, Multidrug-Resistant, Uropathogenic *Proteus mirabilis*. Bionanoscience.

[120] Torabi S., Keshavarzi F. (2025). Agonistic and Antagonistic Effects of both Aqueous and Alcoholic Extracts of Plants containing Copper and Silver Nanoparticles on *Escherichia coli* and *Proteus mirabilis*. Curr. Res. Green Sustain. Chem..

[121] Gurkok S., Ozdal M., Cakici T., Kurbanoglu E.B. (2025). Antimicrobial, antibiofilm, and antiurease activities of green-synthesized Zn, Se, and ZnSe nanoparticles against *Streptococcus salivarius* and *Proteus mirabilis*. Bioprocess. Biosyst. Eng..

[122] Elshikiby L.A., Baka Z.A., El-Zahed M.M. (2025). Biological activities of optimized biosynthesized selenium nanoparticles using *Proteus mirabilis* PQ350419 alone or combined with chitosan and ampicillin against common multidrug-resistant bacteria. Microb. Cell Fact..

[123] Hussein A.A., Aldujaili N.H. (2020). Antimicrobial, antibiofilm, and antioxidant activity of chitosan nanoparticles synthesized by *E. coli*. J. Phys. IOP Publ. Conf. Ser..

[124] Parveen A., Yalagatti M.S., Abbaraju V., Deshpande R. (2018). Emphasized mechanistic antimicrobial study of biofunctionalized silver nanoparticles on model *Proteus mirabilis*. J. Drug Deliv..

[125] Aljobori M., Al-Rawi A.M. (2024). Effect of Chitosan Nanoparticles on Growth, Swarming Motility, and Biofilm Formation in *Proteus mirabilis* Isolated from Urinary Tract Infections. Rafidain J. Sci..

