# Supplementary material for: Effectiveness of Chitosan and Its Nanoparticles Against ampC- and ESBL-Producing Pan-Drug-Resistant Proteus mirabilis in Egyptian Livestock
Source: Pathogens. 2025 Nov 18;14(11):1176. doi: 10.3390/pathogens14111176 (PMC12655785; doi:10.3390/pathogens14111176)
Supplement: Supplementary file 1 [file pathogens-14-01176-s001.zip › Supplementary file S1.pdf]

Effectiveness of chitosan and its nanoparticles against ampC- and ESBL-producing pan-drug resistant *Proteus mirabilis* in Egyptian livestock

Ibtisam Faeq Hasona [1,*], Amal Awad [1,*], Gamal Younis [1] and Wafaa Farouk Mohamed [2]

[1] Department of Bacteriology, Immunology, and Mycology, Faculty of Veterinary Medicine, Mansoura University, Mansoura 35516, Egypt

[2] Ain-Shams University specialized hospital, Cairo, Egypt

***** Correspondence and requests for materials should be addressed to Ibtisam Faeq Hasona, Amal Awad. email: ebtisam.vet_0445@vet.kfs.edu.eg; amalabdo@mans.edu.eg.

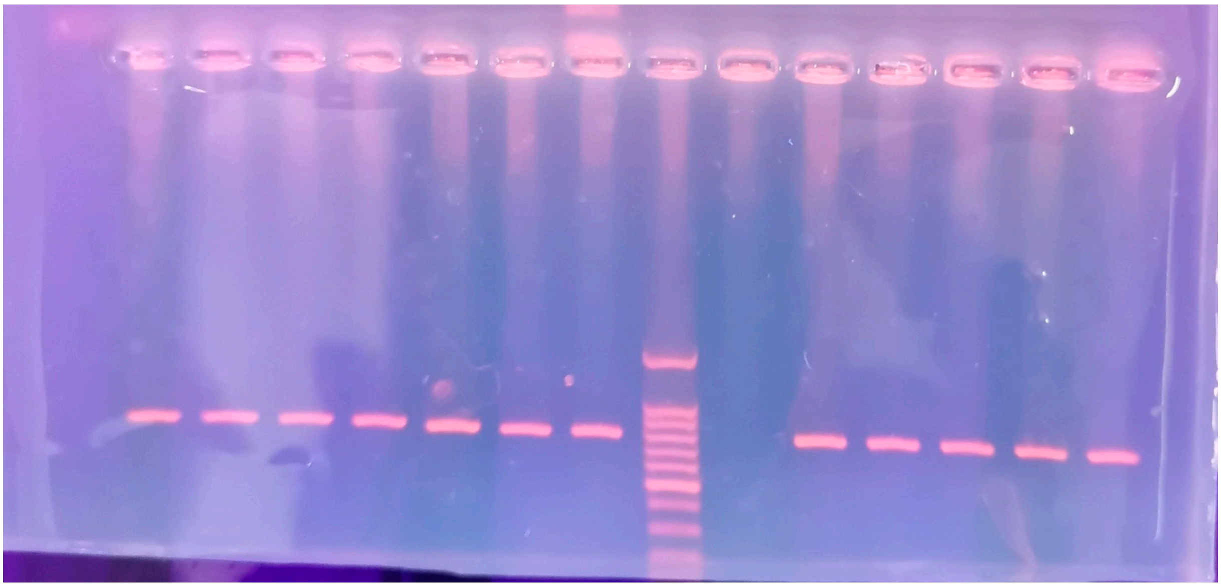

Figure S1: Agarose profile for the detection of *Proteus* spp. specific 16S rRNA is shown at 857 bp

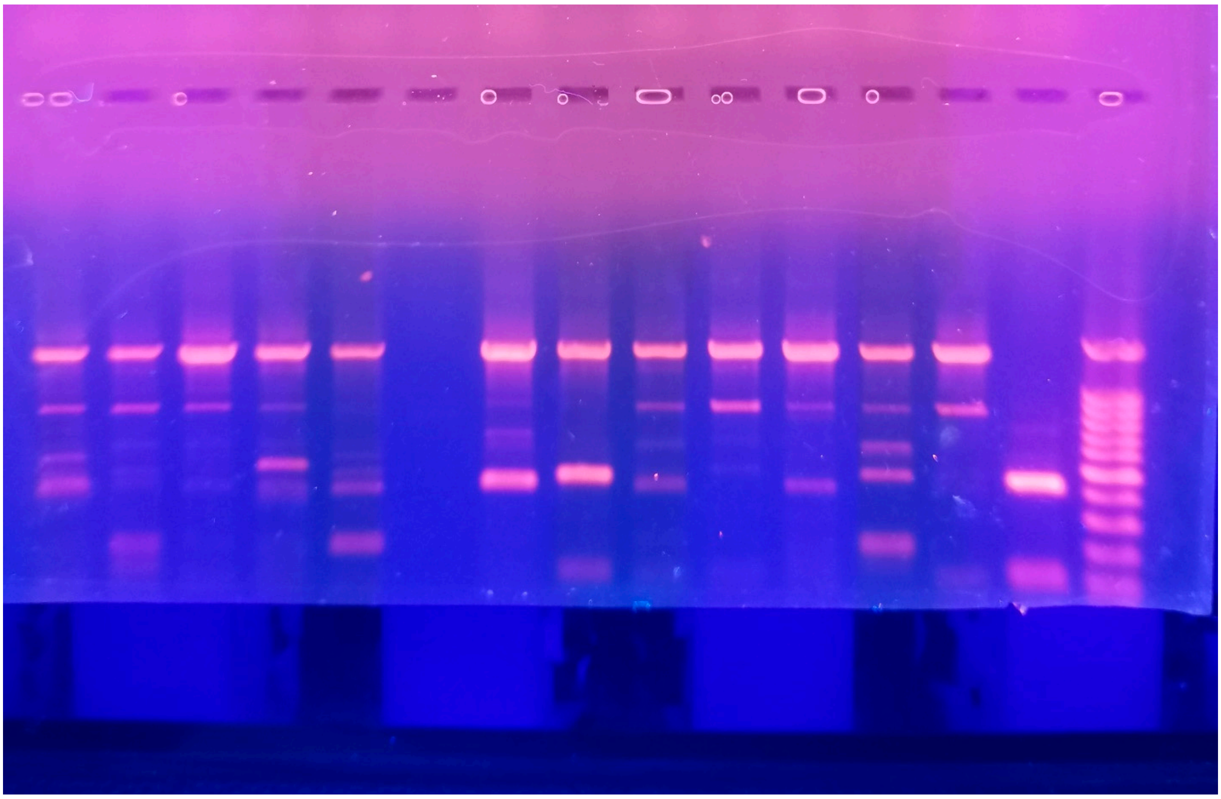

Figure S2: Agarose profile for the detection of *P. mirabilis* specific 16S rRNA is shown at 1496 bp

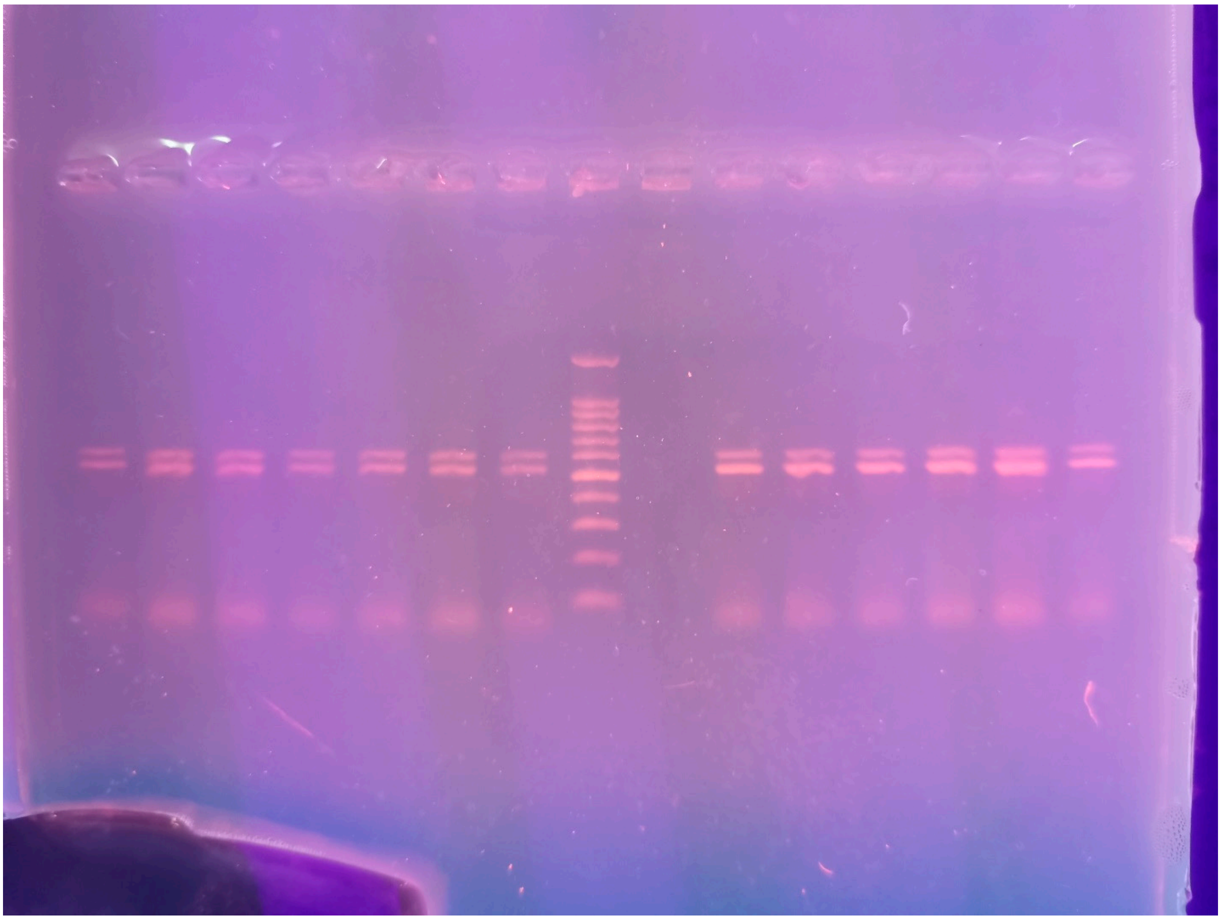

Figure S3: Agarose profile for the detection of resistance genes; *bla*CTX-M (593 bp) and *bla*TEM (516 bp) by duplex PCR

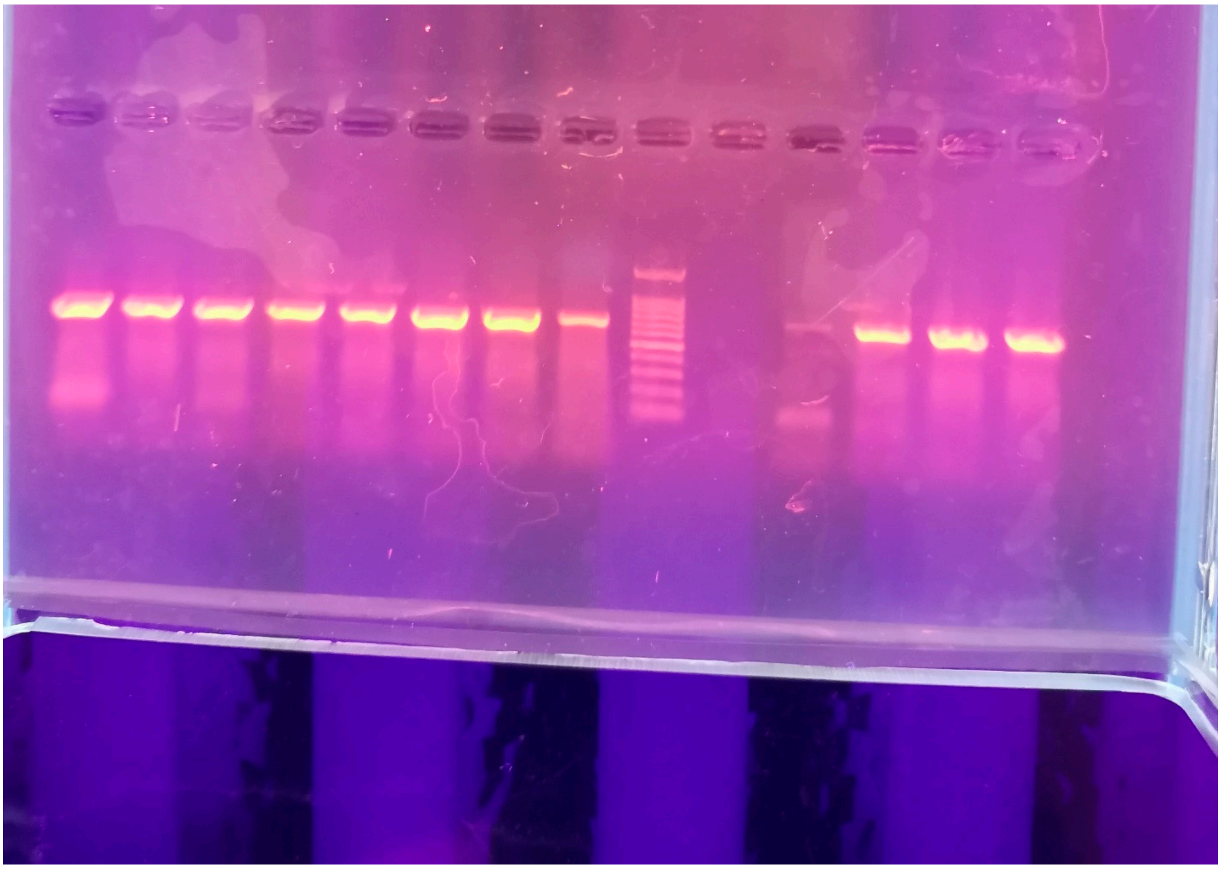

Figure S4: Agarose profile for the detection of *bla*$_{OXA-10}$ (760 bp)

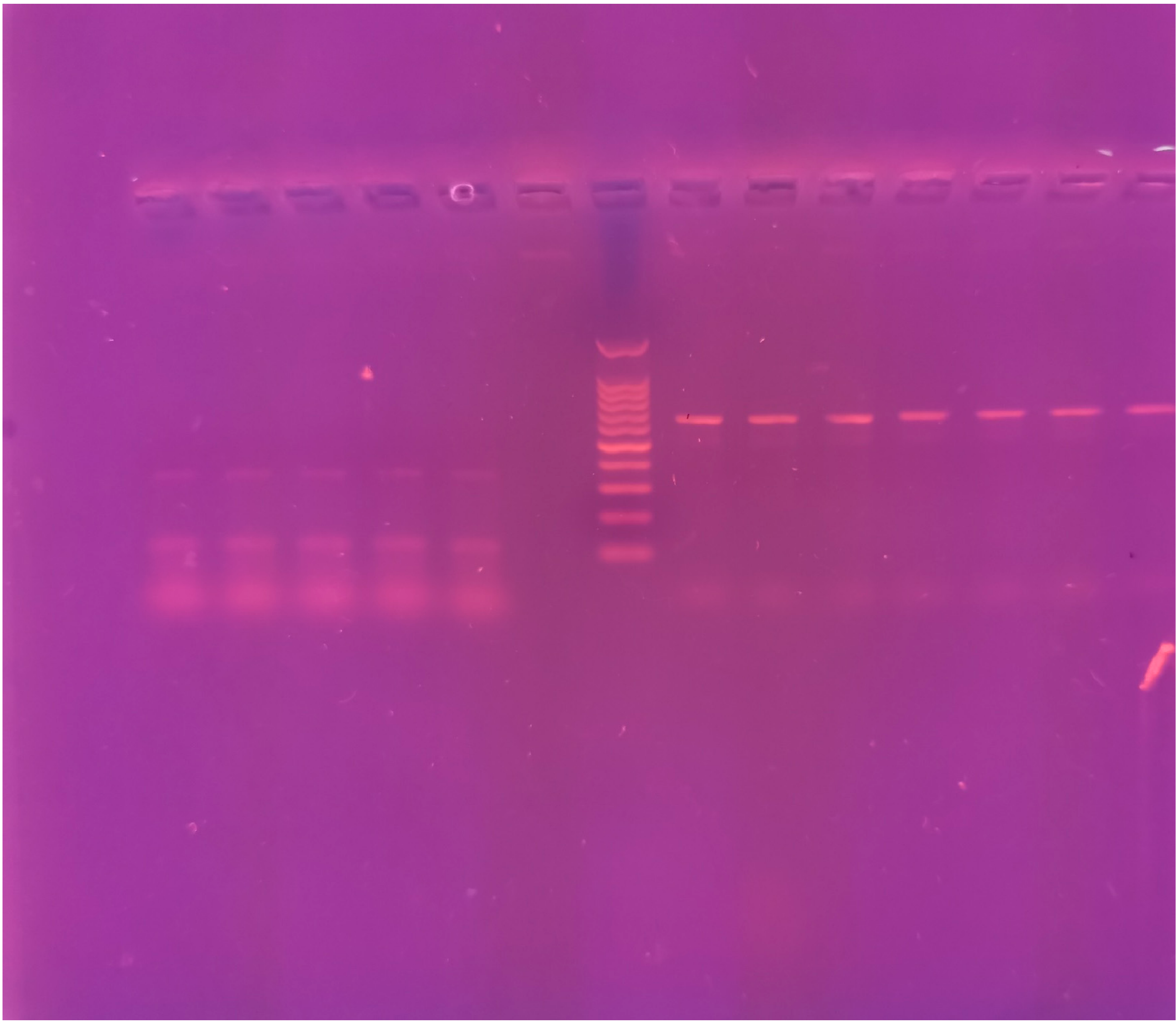

Figure S5: Agarose profile for the detection of resistance gene *bla*$_{CMY-2}$ (695 bp).

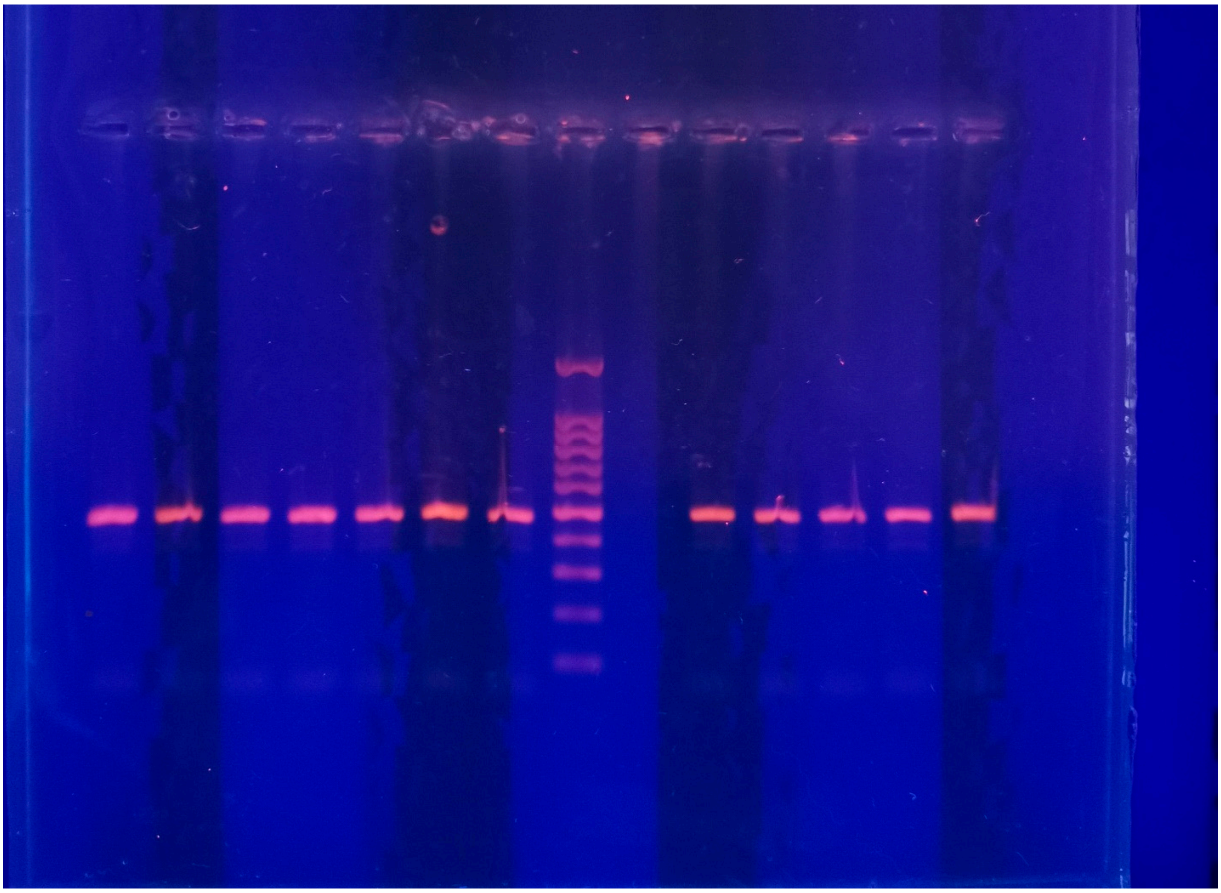

Figure S6: Agarose profile for the detection of resistance gene *aad*A1 (447 bp).

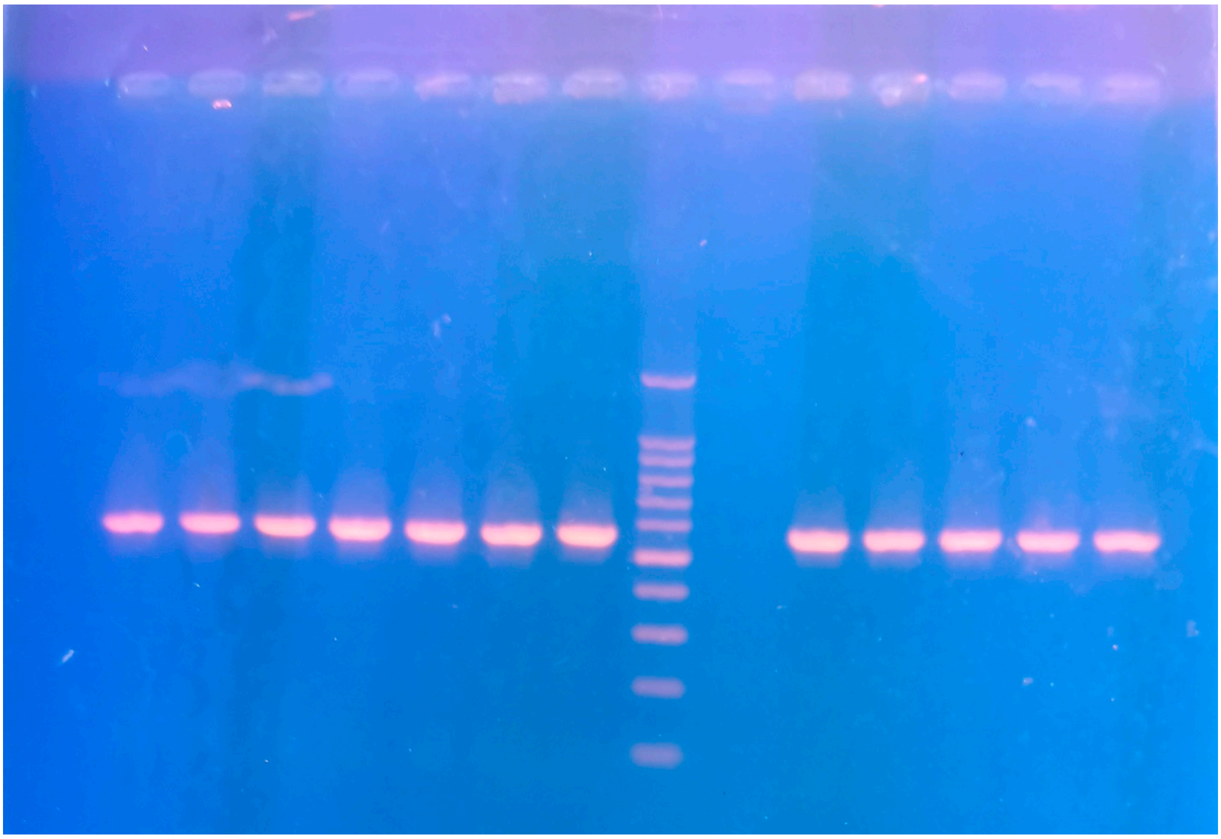

Figure S7: Agarose profile for the detection of resistance gene *cat*A1 (547 bp).

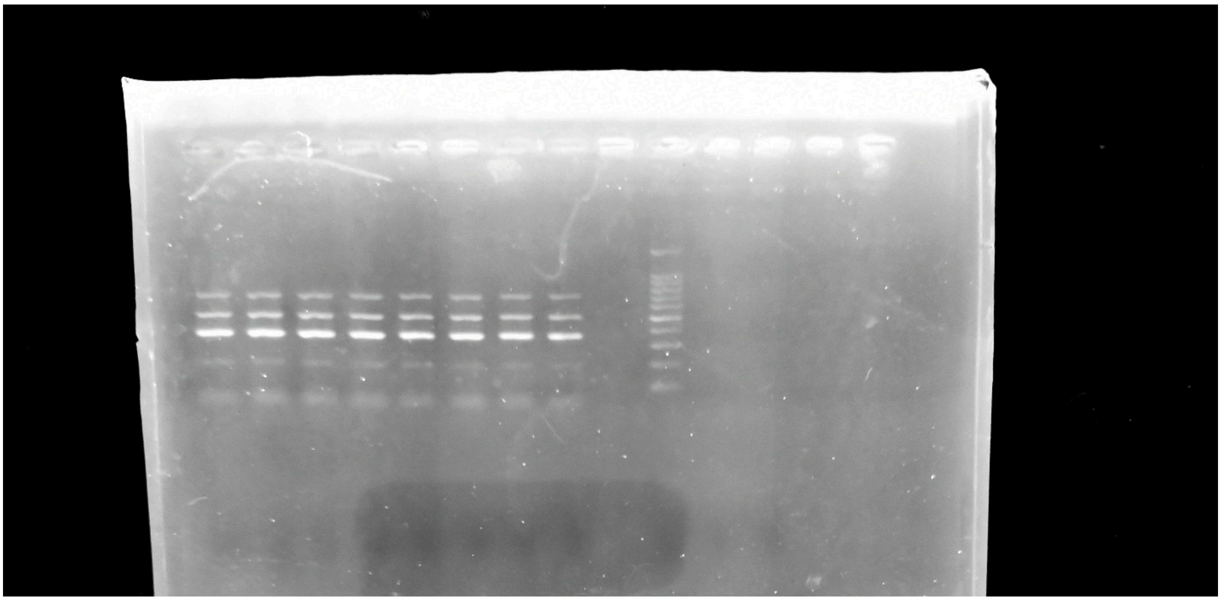

Figure S8: Agarose profile for the detection of resistance genes *dfr*A1 (367 bp), *qnr*A (516 bp), and *sul*2 (722 bp) by multiplex PCR.

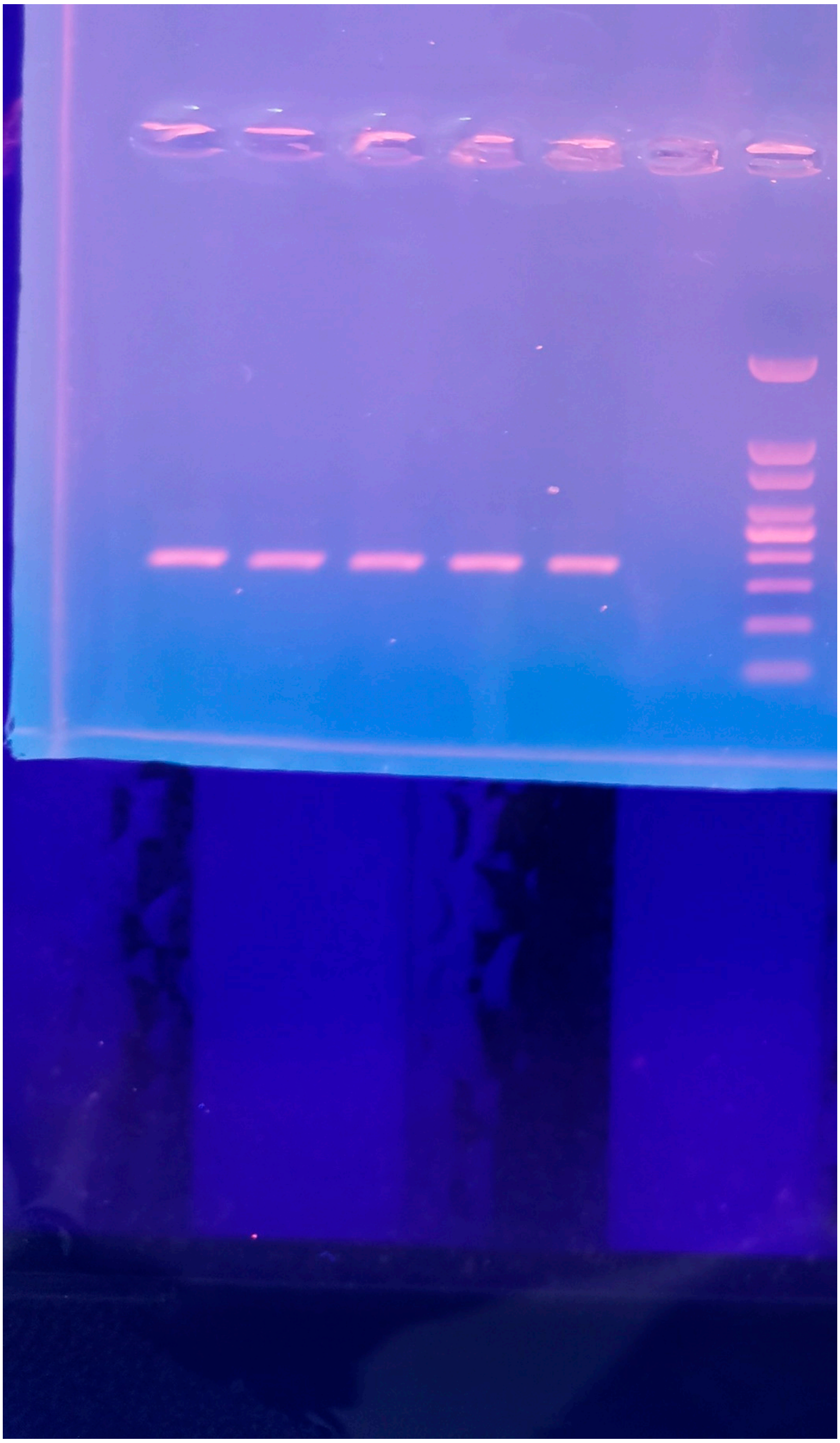

Figure S9: Agarose profile for the detection of resistance gene *tet*M (406 bp).

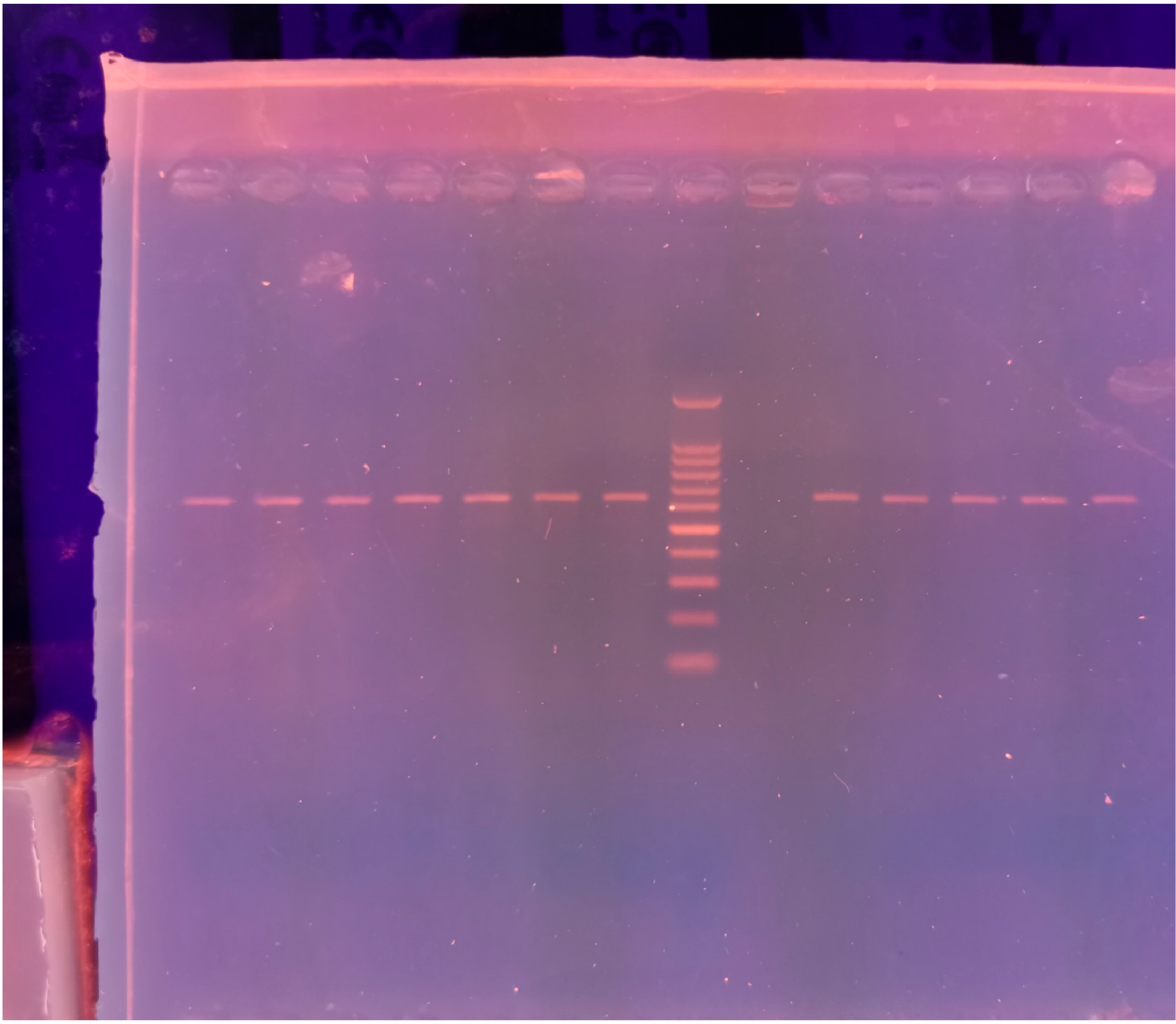

Figure S10: Agarose profile for the detection of resistance gene *erm*B (636 bp).

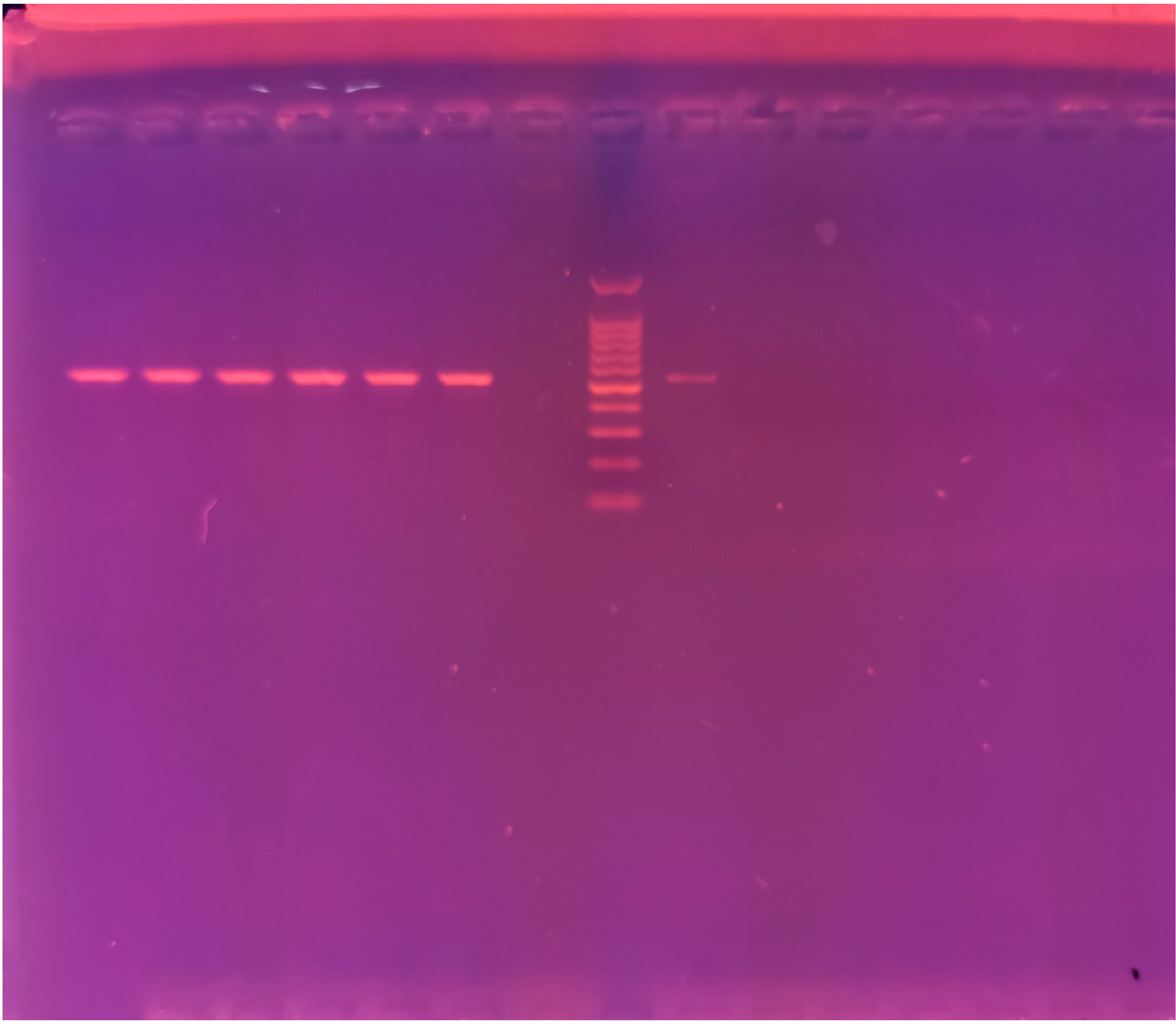

Figure S11: Agarose profile for the detection of resistance gene *int*1 (565 bp)

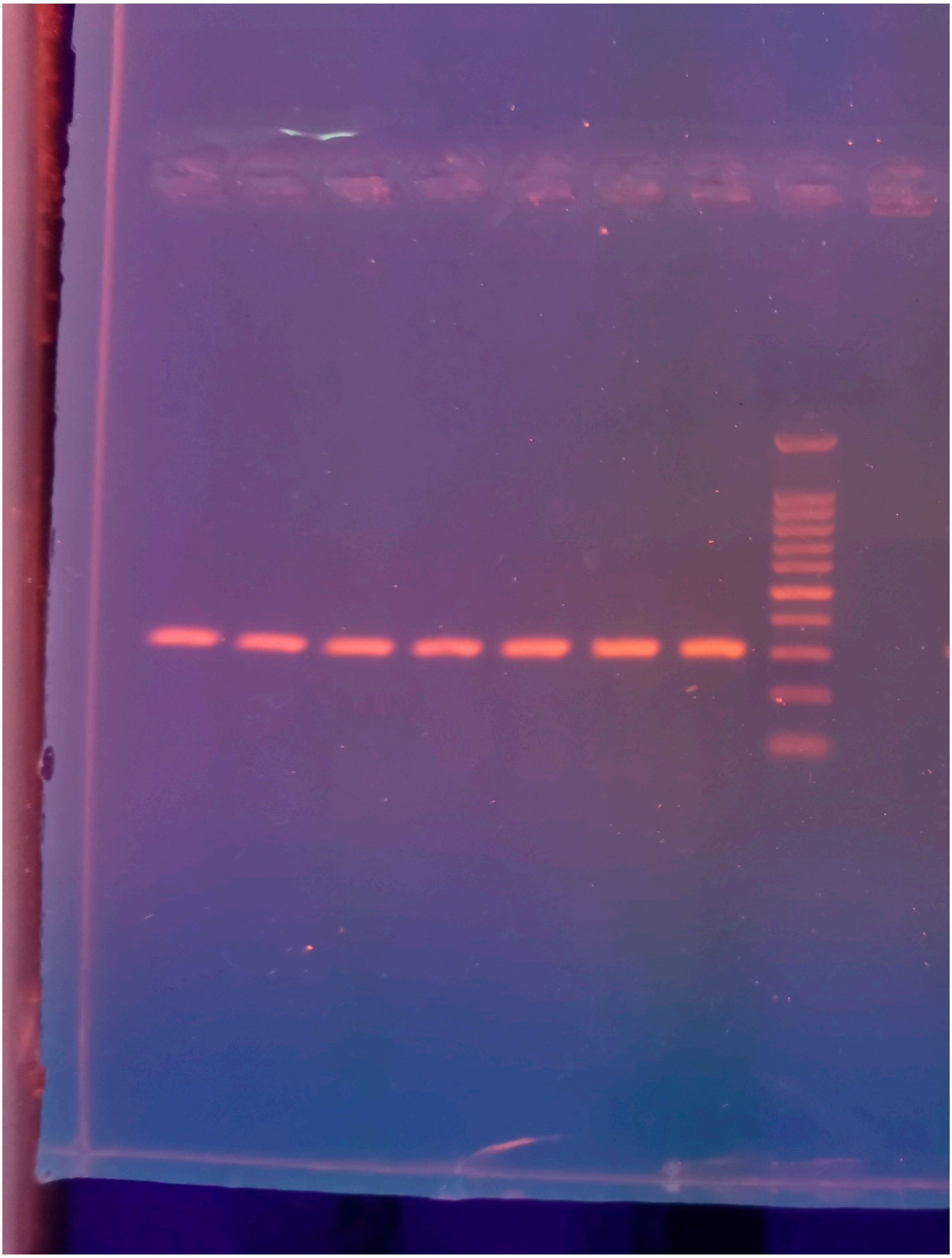

Figure S12: Agarose profile for the detection of resistance gene *mcr*-1 (309 bp)
