# Supplementary material for: Effectiveness of Chitosan and Its Nanoparticles Against ampC- and ESBL-Producing Pan-Drug-Resistant Proteus mirabilis in Egyptian Livestock
Source: Pathogens. 2025 Nov 18;14(11):1176. doi: 10.3390/pathogens14111176 (PMC12655785; doi:10.3390/pathogens14111176)

Size Distribution Report by Volume

v2.2

Malvern

Sample Details

**Sample Name:**	chitosan nano 2
**SOP Name:**	mansettings.nano
**General Notes:**	

**File Name:**	dr ibtsam faiek.dts	**Dispersant Name:**	Water
**Record Number:**	8	**Dispersant RI:**	1.330
**Material RI:**	1.59	**Viscosity (cP):**	0.8872
**Material Absorbtion:**	0.010	**Measurement Date and Time:**	مايو، ٢٠٢٥ ٠٢:٥٨:٣٢ ص 14

System

**Temperature (°C):**	25.0	**Duration Used (s):**	50
**Count Rate (kcps):**	103.2	**Measurement Position (mm):**	4.65
**Cell Description:**	Low volume disposable sizing ...	**Attenuator:**	11

Results

			Size (d.nm):	% Volume:	St Dev (d.n...
**Z-Average (d.nm):**	194.8	**Peak 1:**	140.1	0.3	52.53
**PdI:**	0.973	**Peak 2:**	1500	0.6	536.3
**Intercept:**	0.901	**Peak 3:**	8.663	98.0	1.752

Result quality : **Refer to quality report**

Size Distribution by Volume

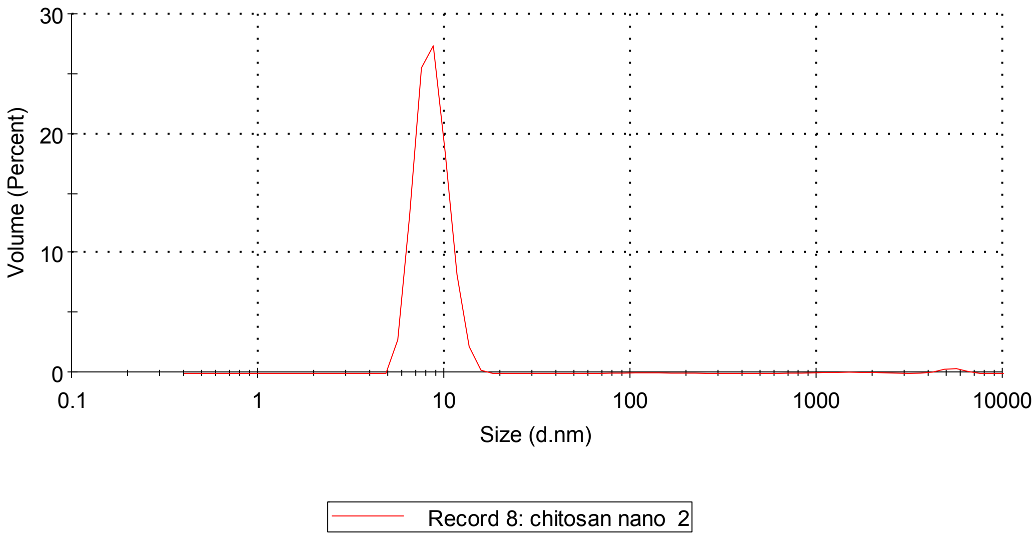

Supplement: Supplementary file 1 [file pathogens-14-01176-s001.zip › Supplementary file S2, Figure S13.pdf]
